# Candidate pathogenicity factor/effector proteins of ‘*Candidatus* Phytoplasma solani’ modulate plant carbohydrate metabolism, accelerate the ascorbate–glutathione cycle, and induce autophagosomes

**DOI:** 10.3389/fpls.2023.1232367

**Published:** 2023-08-18

**Authors:** Marina Dermastia, Špela Tomaž, Rebeka Strah, Tjaša Lukan, Anna Coll, Barbara Dušak, Barbara Anžič, Timotej Čepin, Stefanie Wienkoop, Aleš Kladnik, Maja Zagorščak, Monika Riedle-Bauer, Christina Schönhuber, Wolfram Weckwerth, Kristina Gruden, Thomas Roitsch, Maruša Pompe Novak, Günter Brader

**Affiliations:** ^1^ Department of Biotechnology and Systems Biology, National Institute of Biology, Ljubljana, Slovenia; ^2^ Jožef Stefan International Postgraduate School, Ljubljana, Slovenia; ^3^ Department of Plant and Environmental Sciences, University of Copenhagen, Taastrup, Denmark; ^4^ Department of Functional and Evolutionary Ecology, Faculty of Life Sciences, University of Vienna, Vienna, Austria; ^5^ Department of Biology, Biotechnical Faculty, University of Ljubljana, Ljubljana, Slovenia; ^6^ Federal College and Research Institute for Viticulture and Pomology Klosterneuburg, Klosterneuburg, Austria; ^7^ Bioresources Unit, Health & Environment Department, Austrian Institute of Technology, Tulln, Austria; ^8^ Vienna Metabolomics Center (VIME), University of Vienna, Vienna, Austria; ^9^ Faculty of Viticulture and Enology, University of Nova Gorica, Vipava, Slovenia

**Keywords:** autophagosome, ‘*Candidatus* Phytoplasma solani’, effector, glycolysis, pathogenicity factor, StAMP

## Abstract

The pathogenicity of intracellular plant pathogenic bacteria is associated with the action of pathogenicity factors/effectors, but their physiological roles for most phytoplasma species, including ‘*Candidiatus* Phytoplasma solani’ are unknown. Six putative pathogenicity factors/effectors from six different strains of ‘*Ca*. P. solani’ were selected by bioinformatic analysis. The way in which they manipulate the host cellular machinery was elucidated by analyzing *Nicotiana benthamiana* leaves after *Agrobacterium*-mediated transient transformation with the pathogenicity factor/effector constructs using confocal microscopy, pull-down, and co-immunoprecipitation, and enzyme assays. Candidate pathogenicity factors/effectors were shown to modulate plant carbohydrate metabolism and the ascorbate–glutathione cycle and to induce autophagosomes. PoStoSP06, PoStoSP13, and PoStoSP28 were localized in the nucleus and cytosol. The most active effector in the processes studied was PoStoSP06. PoStoSP18 was associated with an increase in phosphoglucomutase activity, whereas PoStoSP28, previously annotated as an antigenic membrane protein StAMP, specifically interacted with phosphoglucomutase. PoStoSP04 induced only the ascorbate–glutathione cycle along with other pathogenicity factors/effectors. Candidate pathogenicity factors/effectors were involved in reprogramming host carbohydrate metabolism in favor of phytoplasma own growth and infection. They were specifically associated with three distinct metabolic pathways leading to fructose-6-phosphate as an input substrate for glycolysis. The possible significance of autophagosome induction by PoStoSP28 is discussed.

## 1 Introduction

Phytoplasma is a diverse genus of plant pathogenic bacteria in the class Mollicutes that causes several economically important insect-transmitted diseases (Bertaccini and Lee, 2018). Although information on their biology and pathogenicity is still very rudimentary, the life of these plant pathogens is gradually being unraveled with the help of appropriate bioinformatic analyzes and molecular biology approaches.

Phytoplasma exclusively inhabits the sieve cells of the phloem and lacks many genes involved in metabolic pathways that are important for free-living cells (Namba, 2019). In acquiring nutrients from their plant host, phytoplasma competes with hosts for the same nutrient substrates. Under conditions of phytoplasma infection, the metabolic networks of the invading pathogen and its host should be closely linked, and even a minor change in metabolism could significantly affect the outcome of the phytoplasma–host interaction. Therefore, phytoplasma is thought to modulate host plant cell metabolism to provide nutrients, energy, and metabolites for successful replication and infection in plants (Prezelj et al., 2016a; Dermastia, 2019; Dermastia et al., 2019).

Phytoplasma genomes lack homologs of the type III secretion system (Kube et al., 2012), which forms hollow tubes through which effectors from the bacterial cytosol pass directly into the host cell cytosol and are essential for the virulence of most Gram-negative bacterial pathogens (Abramovitch et al., 2006). Instead, phytoplasma uses a functional Sec-dependent pathway for the export of proteins across the cytoplasmic membrane to the bacterial periplasm and outer membrane (Green and Mecsas, 2016). Phytoplasma also uses the Sec-dependent system for secretion of pathogenicity factors or effectors directly into the cytoplasm of plant host phloem sieve cells (Bai et al., 2009; Hoshi et al., 2009; MacLean et al., 2014; Huang et al., 2021). Phytoplasma candidate effector proteins can be identified by searching for their cleavable signal peptides associated with a functional Sec-dependent pathway (MacLean et al., 2011). Once inside the host cell, effectors often target key proteins to hijack the host cellular machinery and remodel the signaling cascade (Tomkins et al., 2018). Some known phytoplasma effectors target developmental and immunity-related host transcription factors (Marrero et al., 2020). Most of the phytoplasma effectors studied in detail are derived from ‘*Candidatus* Phytoplasma asteris’ such as the secreted AY-WB proteins (SAPs) from the witches’ broom (AY-WB) strain (Bai et al., 2009) and tengu-su inducer (TENGU) from the onion yellows (OY) strain (Hoshi et al., 2009). The secreted AY-WB protein SAP 11 (SAP11) is involved in the development of witches’ broom symptoms by interacting with the transcription factors TEOSINTE BRANCHED 1, CYCLOIDEA, and proliferating cell nuclear antigen factor 1 (PCF1), whereas the development of leaf-like flowers is related to the degradation of MADS-box transcription factors by SAP54 (Tomkins et al., 2018). SAP05 can hijack the plant ubiquitin receptor regulatory particle non-ATPase 10 (RPN10) independently of substrate ubiquitination, allowing phytoplasma to alter plant architecture and reproduction (Huang et al., 2021). The action of TENGU is related to auxin and jasmonic acid signaling by repressing the expression of auxin response factor 6 and 8 genes, resulting in dwarfism and floral sterility. Several homologs of these effectors have been discovered in other phytoplasma genomes (Lu et al., 2014; Siewert et al., 2014; Anabestani et al., 2017; Janik et al., 2017; Pecher et al., 2019; Zhou et al., 2021; Bai et al., 2022; Bai et al., 2023).

The molecular mechanisms of other putative secreted pathogenicity factors/effectors by which phytoplasma manipulate their hosts are not known, making the understanding of the general mechanisms of phytoplasma interaction with host plants elusive. However, a recent study suggests that phytoplasma has a unique infection strategy and that their effectors have unusual targets (Marrero et al., 2020). In addition to effectors released in plant and insect host cells, phytoplasma membrane–associated proteins are also important for potential recognition or interaction with the host. For example, the major antigenic membrane protein (Amp) of ‘*Ca.* P. asteris’ OY in insects co-localizes with microfilaments of intestinal tract muscles and can form complexes with actin and myosin heavy and light chains and ATPase subunits. It has been suggested that these interactions may be insect species specific (Suzuki et al., 2006; Galetto et al., 2011), but their role, if any, in the plant cell is unknown.

‘*Ca.* P. solani’ from ribosomal group 16SrXII-A (Quaglino et al., 2013) is associated with the most widespread phytoplasma grapevine (*Vitis vinifera*) disease in Europe, namely, Bois noir (Dermastia et al., 2017), and with stolbur disease of plants belonging to family Solanaceae, including potato (*Solanum tuberosum*), tomato (*Solanum lycopersicum*), and tobacco (*Nicotiana* sp.). Solanaceous disease is already a growing problem in Serbia (Mitrović and Duduk, 2011; Mitrović et al., 2016), Romania (Ember et al., 2011) and Turkey (Çağlar et al., 2010) and has occurred sporadically in Central Europe including Germany (Preiss et al., 2008). Recently, severe outbreaks have been reported, especially in potatoes in eastern Austria (personal reports of G. Brader and M. Riedle-Bauer).

Studies on the interactions between host grapevines and ‘*Ca.* P. solani’ regarding transcription, regulation, proteins, and metabolism show that infection affects biotic stress signaling, hormonal balance, photosynthesis, oxidative stress processes, and secondary metabolism (Hren et al., 2009; Balakishiyeva et al., 2018; Rotter et al., 2018; Škrlj et al., 2021; Dermastia et al., 2021). All these studies also indicate that carbohydrate metabolism is severely impaired in infected grapevines, as evidenced by the altered activity of the corresponding enzymes and/or genes encoding them. In addition, a combined analysis of high-throughput RNA sequencing (RNA-Seq) and small RNA sequencing (sRNA-Seq) data from cv. ‘Zweigelt’ infected with ‘*Ca*. P. solani’ at the beginning and at the end of the growing season showed that the early growing season is very dynamic in asymptomatic grapevines at the transcriptional level, whereas the regulation at the small RNA level is more pronounced later in the season when symptoms develop in infected grapevines. Most differentially expressed small RNAs were associated with biotic stress (Dermastia et al., 2021).

Recent sequencing and assembly of the ‘*Ca.* P. solani’ strain SA-1 genome identified 38 putative secreted protein/effector genes (Seruga Music et al., 2019). Twenty of these genes are located within phytoplasma potential mobile units. These regions exhibit variations in gene order intermixed with genes of unknown function and a lack of similarity to other phytoplasma genes, suggesting that they are prone to rearrangements and acquisition of new sequences through recombination. Five of the putative effectors were unique to the sequenced strain and two of them resembled proteins secreted by AY-WB (Seruga Music et al., 2019). However, the roles of the identified putative effectors have not been elucidated.

Here, we report the identification of six potential pathogenicity factors/effectors in six different ‘*Ca*. P. solani’ strains. In addition, we amplified these pathogenicity factors/effectors from a selected strain originated from the infected grapevine cv. ‘Zweigelt’ and demonstrated their cellular and metabolic targets contributing to the pathogenicity of this phytoplasma.

## 2 Materials and methods

### 2.1 Bioinformatic analysis

Potential effector proteins of ‘*Ca*. P. solani’ were screened in the genome of strain SA-1 (Seruga Music et al., 2019) (genome accession number: NZ_MPBG00000000) with a prediction of signal peptide cleavage sites by SignalP 4.1 (Nielsen, 2017), which discriminates signal peptides from transmembrane regions, and by LipoP 1.0, which discriminates between lipoprotein signal peptides, other signal peptides, and N-terminal membrane helices. Although LipoP 1.0 has been trained only on sequences from Gram-negative bacteria, it also performs well on sequences from Gram-positive bacteria (Rahman et al., 2008), including Mollicute proteins (Bai et al., 2009). Transmembrane helices in proteins were predicted using TMHMM 2.0 (Krogh et al., 2001). Genes encoding potential effectors or membrane exposed proteins identified in this way were additionally determined by PCR (primers in **Supplemental Table S1**, except for the previously published PoStoSP28 = StAMP) for different genotypes of ‘*Ca*. P. solani’ found during surveys in Austria. These include the two nettle associated genotypes CPsM4_At1 [nomenclature and strain origins published in Aryan et al. (2014); SEE-ERANET marker gene identities of tuf, stamp, and vmp1 are tuf-b2, stamp-6, and V18, respectively; for overview, see **Tables 1**, **2**] and CPsM4_At4 (tuf-a, stamp-46, and V3) and four additional bindweed associated genotypes in addition to SA-1. These are CPsM4_At6 (tuf-b1, stamp-4, and V4), CPsM4_At7 (tuf-b1, stamp-4, and V14), CPsM4_At10 (tuf-b1, stamp-9, and V17), and CPsM4_At12 (tuf-b1, stamp-9, and V4). The DNA of all the strains corresponding to the genotypes originates from insect transmitted *Catharanthus roseus*, with the exception of VvKn_Zw2 (genotype CPsM4_At1) and VvKn4 (genotype CPsM4_At7), which were derived from ‘*Ca.* P. solani’ grapevine cv. ‘Zweigelt’ (Aryan et al., 2014; Mehle et al., 2022) (**Tables 1**, **2**). To visualize the similarity of the protein sequences, the sequences were aligned using Geneious Alignment in Geneious Prime 2020.1.1 (https://www.geneious.com/) (**Supplemental Figure S6**).

**Table 1:** List of candidate effectors of ‘*Candidatus* Phytoplasma solani’.

Name of putative effector	Annotation	Signal cleaving	Number of predicted transmembrane helices in protein	Locus tag in strain SA-1	Length (amino acids)	Prediction of localization
PoStoSP04	Putative effector (‘*Ca.* P. solani’)	Mature chain: 33–258	1	PSSA1_RS01075 (PSSA1_v1c2050)	260	No predicted locations
PoStoSP06	Putative effector (‘*Ca.* P. solani’)	Mature chain: 32–506	1	PSSA1_RS02495 (PSSA1_v1c4830)	506–507	Chloroplast, nucleus
PoStoSP13	Putative effector (‘*Ca.* P. solani’)	Mature chain: 35–107	1	PSSA1_RS03585 (PSSA1_v1c6790)	261–272	No predicted locations
PoStoSP14	Putative effector (‘*Ca.* P. solani’)	Mature chain: 34–629	2	PSSA1_RS03580 (PSSA1_v1c6780) Truncated in SA-1	630–667	Nucleus
PoStoSP18	ABC-type sugar transport system, extracellular solute- binding protein (‘*Ca.* P. solani’)	Mature chain: 32–540	1	PSSA1_RS01645 (PSSA1_v1c3190)	540	No predicted locations
PoStoSP28	Antigenic membrane protein (‘*Ca.* P. solani’); StAMP	Mature chain: 32–157	2	PSSA1_RS01265 (PSSA1_v1c2420)	157–164	No predicted locations

**Table 2:** ‘*Candidatus* Phytoplasma solani strains investigated in this study.

Strain	Genotype	Bindweed/nettle type	Origin	Similarity of nucleotide/AA sequences in % compared to SA-1					
				PoStoSP04	PoStoSP06	PoStoSP013	PoStoSP014	PoStoSP018	PoStoSP028
SA-1	Genome acc. No.: NZ_MPBG01000000	bindweed	Vv*	100/100	100/100	100/100	100/100	100/100	100/100
VvKn_Zw2	CPsM4_At1	nettle	Vv	99/100	98/96	99/99	98/93	99/98	96/90
CrHo13_1183	CPsM4_At4	nettle	Cr ex Ho	99/100	98/96	99/99	98/91	99/98	95/89
CrHo12_601	CPsM4_At6	bindweed	Cr ex Ho	99/100	99/97	99/98	98/93	100/100	100/100
Vv_Kn4	CPsM4_At7	bindweed	Vv	99/100	98/96	99/98	99/96	100/100	100/100
CrHo12_721	CPsM4_At10	bindweed	Cr ex Ho	99/100	98/96	-/-	99/96	99/99	97/96
CrAr12_722_2	CPsM4_At12	bindweed	Cr ex Ar	99/100	98/96	99/99	99/96	99/99	97/96

Vv, *Vitis vinifera*; Cr ex Ho, *Catharanthus roseus* infected by *Hyalesthes obsoletus*; Cr ex Ar, *C. roseus* infected by *Anaceratagallia ribauti*; all collections from Eastern Austria.

### 2.2 Plasmid constructs

Full-length coding sequences of the six putative effector proteins (PoStoSP04, PoStoSP06, PoStoSP13, PoStoSP14, PoStoSP18, and PoStoSP28) from a selected ‘*Ca*. P. solani’ strain VvKn_Zw2 (**Table 2**) and of two grapevine phosphoglucomutases (*Vitvi*16g00891 and *Vitvi*01g00455) were amplified from material infected with the CPsM4_At1 genotype of grapevine cv. ‘Zweigelt’ (Aryan et al., 2014) (**Table 2**) and harvested in an experimental vineyard in Klosterneuburg (Austria) using the Phusion^®^ High-Fidelity PCR Kit (New England Biolabs, USA), and the primer pairs are listed in **Supplemental Table S1**. The correct size products were selected on an agarose gel and purified using the Wizard^®^ SV Gel and PRC Clean-Up System Kit (Promega, USA). Phosphoglucomutase sequences were first cloned into the pJET1.2/blunt vector using the CloneJET PCR Cloning Kit (Thermo Scientific, USA). The amplified sequences were then cloned into the pENTR D-TOPO vector using the pENTR™ Directional TOPO^®^ Cloning Kit (Invitrogen, USA) and SANGER-sequenced (Eurofins Genomics, Germany). The effector sequences were recombined through LR reaction with the Gateway^®^ LR Clonase TM II Enzyme Mix (Invitrogen, USA) into pH7WGY2 Gateway destination vectors containing enhanced yellow fluorescent protein (YFP) (VIB, Belgium). For the co-immunoprecipitation (IP) assay, the phosphoglucomutase sequences were cloned into the pJCV52 Gateway vector (VIB, Belgium) (Karimi et al., 2002) containing the hemagglutinin A (HA) tag.

In addition to the vectors containing effector sequences, three other constructs were used for co-localization studies, namely, histone 2B (H2B) fused to monomeric red fluorescent protein 1 (mRFP1) (Lukan et al., 2018) was used for nucleus localization; a construct containing *Arabidopsis thaliana* remorin 1.3 (REM1.3) fused to mCherry (Marín et al., 2012) was used for plasmalemma localization; and a construct with autophagy-related protein (ATG) (ATG8CL) fused to mRFP (Dagdas et al., 2016) was used for autophagosome localization.

### 2.3 *Agrobacteria*-mediated transformation

Transient transformation of 3- to 4-week-old leaves of *Nicotiana benthamiana* was performed using *Agrobacterium tumefaciens* strain GV3101 carrying the constructs described above. Briefly, cultures were grown overnight, pelleted, washed with lysogeny broth (LB) medium, and re-pelleted. They were then resuspended in an infiltration solution consisting of 10 mM 2-(N-morpholine)ethanesulfonic acid (MES), 10 mM MgCl_2_, and 0.2 mM acetosyringone to A600 of 0.5. For co-expression and co-localization, agrobacteria cultures carrying the appropriate vector constructs were mixed in equal ratios. In all cases, an equal volume of agrobacteria culture carrying the p19 silencing suppressor was added. Leaves of *N. benthamiana* plants were infiltrated with the agrobacteria mix using a syringe. Plants were maintained at 21 ± 2°C in growth chambers, with an illumination of 70 µM m^−2^ s^−1^ (Osram L36W/77 lamp), a photoperiod of 16 h and relative humidity of 70%.

### 2.4 Confocal imaging

Fluorescent protein expression was followed 3 and 4 days after agroinfiltration using two different laser scanning confocal microscopes. The Leica TCS SP5 laser scanning confocal microscope mounted on a Leica DMI 6000 CS inverted microscope (Leica Microsystems, Wetzlar, Germany) with a 63× objective with zoom factor 1 was used for localization studies. Sequential scanning was performed (sequential setting 1: 543-nm laser, emission window of 555–637 nm; sequential setting 2: 488- and 514-nm laser, emission windows of 529–594 nm and 703–800 nm, and transmission). Two to five regions of interest per agroinfiltrated area were scanned unidirectionally with zoom factor 1, line average 2, and scan speed of 400 Hz. The Leica TCS LSI laser scanning confocal microscope mounted on a Leica Z6 APOA microscope (Leica Microsystems) with a 20× objective was used for co-localization with the ATG8 CL marker. The 488- and 532-nm lasers were used for excitation of enhanced YFP, enhanced green fluorescent protein (GFP), and mRFP1, respectively. YFP emission was measured in the window from 525 to 550 nm, GFP emission in the window from 505 to 525 nm, and mRFP1 emission in the window from 600 to 620 nm. For localization, three regions of interest per agroinfiltrated area were scanned unidirectionally with zoom factor 3, line average 3, and scan speed of 600 Hz. The overlay of images acquired in different channels and maximum projections from Z-stacks were performed using Leica LAS AF Lite software (Leica Microsystems, Wetzlar, Germany).

### 2.5 Protein pull-down and co-immunoprecipitation assay

For protein pull-down, YFP-tagged PoStoSP28 (StAMP) was transiently expressed in *N. benthamiana* leaves for 5 days, and fluorescence was confirmed with confocal microscopy (effector sample). Leaves infiltrated only with the p19 silencing suppressor were used as controls (control sample). Total proteins were extracted from ~200 mg of leaf material with IP buffer containing 25 mM Tris-HCl (pH 7.5), 100 mM NaCl, 10 mM dithiotreitol, 0.1 mM phenylmethylsulfonyl fluoride (PMSF), 0.02% NP-40, 10% glycerol, and cOmplete™ ULTRA Tablets, Mini, EDTA-free Protease Inhibitor Cocktail (Roche, Basel, Switzerland). Protein extraction was followed by incubation with GFP-Trap^®^ Magnetic Agarose beads (ChromoTek, Planegg, Germany) at 4°C for 1–2 h. The beads were washed three times with IP buffer without NP-40 and eluted in sodium dodecyl sulfate–polyacrylamide gel electrophoresis (SDS-PAGE) loading buffer containing 100 mM Tris-HCl (pH 6.8), 4% SDS, 0.2% bromophenol blue, 20% glycerol, and 200 mM dithiothreitol. The immunoprecipitated proteins in the effector and control samples were then analyzed by SDS-PAGE, followed by tryptic digestion.

For the co-IP assay, YFP-tagged PoStoSP28 (StAMP) was co-expressed in *N. benthamiana* leaves for 4 days with one of the HA-tagged phosphoglucomutase proteins. As a control, the phosphoglucomutases were co-expressed with GFP encoded by the pB7WGF2 vector (Karimi et al., 2002), whereas each of the interactors was also expressed separately. Fluorescence of YFP-tagged PoStoSP28 (StAMP) or GFP was confirmed by confocal microscopy. Approximately 500 mg of leaf material was used for protein extraction and IP, which was performed as described above, with an additional dilution step in IP buffer without NP-40 after protein extraction (extract to buffer ratio 1:2.5). The immunoprecipitated proteins and protein extracts were analyzed by SDS-PAGE and Western blot, using anti-GFP (diluted 1:3,000, Invitrogen, USA) and anti-HA (diluted 1:1,000, ChromoTek, Planegg, Germany) antibodies.

### 2.6 Protein sequencing

Bands of approximately 1 mm × 10 mm in size were excised from the effector and control sample lanes at approximately the same height from the SDS-PAGE gel stained with PageBlue™ Protein Staining Solution (Thermo Scientific, USA). Protein reduction in the gel, alkylation, and tryptic digestion was performed as described by Olsen et al. (2004). Briefly, gel bands were cut into 1-mm^2^ pieces and de-stained. After protein reduction with dithiothreitol (20 min at 56°C) and alkylation with iodoacetamide (20 min at room temperature in dark), the samples were digested overnight at 37°C with trypsin (Sigma-Aldrich, USA). Gel protein digests were extracted with 30% acetonitrile and 1% trifluoroacetic acid. The samples were desalted using Bond Elut OMIX C18 tips (Agilent, USA) and dried completely.

Gel protein digests were redissolved in 2% acetonitrile and 0.1% formic acid, ultrasonicated for 15 s, and centrifuged (10,000 x g, 5 min, 4°C). A ultra high performance liquid chromatography (uHPLC) system (Dionex Ultimate 3000, Thermo Fischer Scientific) with a flow rate of 300 nl/min was used. The column (Thermo Scientific Easy Spray column) was loaded with the protein digests, and the peptides were eluted with a 90-min gradient from 2% to 90% acetonitrile containing 0.1% formic acid. Mass spectrometry (MS) analysis was performed using a Thermo Scientific Orbitrap Elite mass spectrometer (Thermo Scientific). A data-dependent top-20 method was used for dynamically selecting the most abundant precursor ions from the survey scan (400–1,800 m (mass)/z (charge number of ions) and a resolution of 30,000). The default charge state was set to two-fold charge; unassigned charge states and +1 charge states were rejected. The minimal required signal was set to 1,000, the size of the exclusion mass list was set to 500 (with a duration of 60 s), and the exclusion mass width was set to 4 ppm (parts per million) with one repeated count of 30 s.

Analysis of MS data was performed using MaxQuant (1.6.5.0). Raw files were searched against a FASTA file of the Uniprot-proteome 7360 (AUP000084051 4097, *N. tabacum*, July2019). For tryptic peptides, a maximum of two missing cleavages and a maximum of three modifications per peptide (oxidation M and N-terminal acetylation) were allowed. Precursor mass tolerance was set at 4.5 ppm [Fourier-transform mass spectrometry - (FTMS)] and 0.6 Da [ion trap mass spectrometry - (ITMS)]. To exclude random matches, data were searched against a database of revert sequences in a target-decoy approach. Only high confidence peptides [false discovery rate - (FDR0 < 0.01%)] and proteins with at least two distinct identified peptides fulfilled the identification criteria. The MS proteomics data were submitted to the ProteomeXchange Consortium via the PRIDE partner repository (Perez-Riverol et al., 2019) with dataset identifier PXD015374. Target proteins were selected on the basis peptide identification in the gel bands of the effector sample but not in the control sample. Grapevine orthologues of *N. tabacum* target proteins were identified with protein BLAST, using the VCost.v3 version of the 12X.v2 version of the grapevine genome assembly (Canaguier et al., 2017).

### 2.7 Enzyme activity assays

The activity of 12 enzyme involved in carbohydrate metabolism (i.e., ADP-glucose pyrophosphorylase, phosphoglucomutase, aldolase, phosphofructokinase, phosphoglucoisomerase, hexokinase, fructokinase, glucose-6-phosphate dehydrogenase, uridine diphosphate glucose (UDP-glucose) pyrophosphorylase, cytoplasmic invertase, cell wall invertase, and vacuolar invertase) and nine enzymes associated with the oxidative stress (i.e., ascorbate peroxidase, catalase, dehydroascorbate reductase, glutathione *S*-transferase, glutathione reductase, monodehydroascorbate reductase, peroxidase, apoplastic peroxidase, and superoxide dismutase) were tested. All enzyme activity assays were performed in either grapevine or *N. benthamiana* leaf material. Grapevine material was obtained from grapevine cv. ‘Zweigelt’ naturally infected with ‘*Ca*. P. solani’ genotype CPsM4_At1 (**Table 2**) in a vineyard in Klosterneuburg (Austria). Transiently transformed *N. benthamiana* leaves were infiltrated with a mixture of agrobacteria, harboring either an effector expression cassette and the RNA silencing suppressor p19 (i.e., effector expressing samples) or p19 only (i.e., control samples). Leaves from non-transformed plants were also analyzed (intact control samples). For transformed plants, enzyme activity was determined in the agroinfiltrated leaf and in the leaf above, referred here as the systemic leaf. Leaves were sampled 3 and 14 days after agroinfiltration. Extraction of enzymes from leaf material was performed exactly as previously described (Jammer et al., 2015; Covington Dunn et al., 2016; Fimognari et al., 2020).

Enzyme activity assays were performed in UV-transmissive flat bottom 96-well plates (UV-Star Greiner Bio One, Kremsmünster, Austria). Protein extract volumes ranging from 1 to 20 μL were used for the reactions. The total reaction volume was 160 μL. Reaction mixtures were incubated in a plate reader (Ascent Multiskan, Thermo Fisher Scientific, Waltham, USA) for 40 min at 25°C or 30°C according to the optimized protocol for each enzyme. All assays were performed in triplicate, and no substrate was added to the reaction mixtures in the control assays. The change in absorbance per second was used to calculate enzyme activity in nkat/g protein. The activity of enzymes related to carbohydrate metabolism was determined according to Jammer et al. (2015). Anitoxidant metabolism-related enzyme activity assays were performed according to Fimognari et al., 2020.

**Figure 1:**
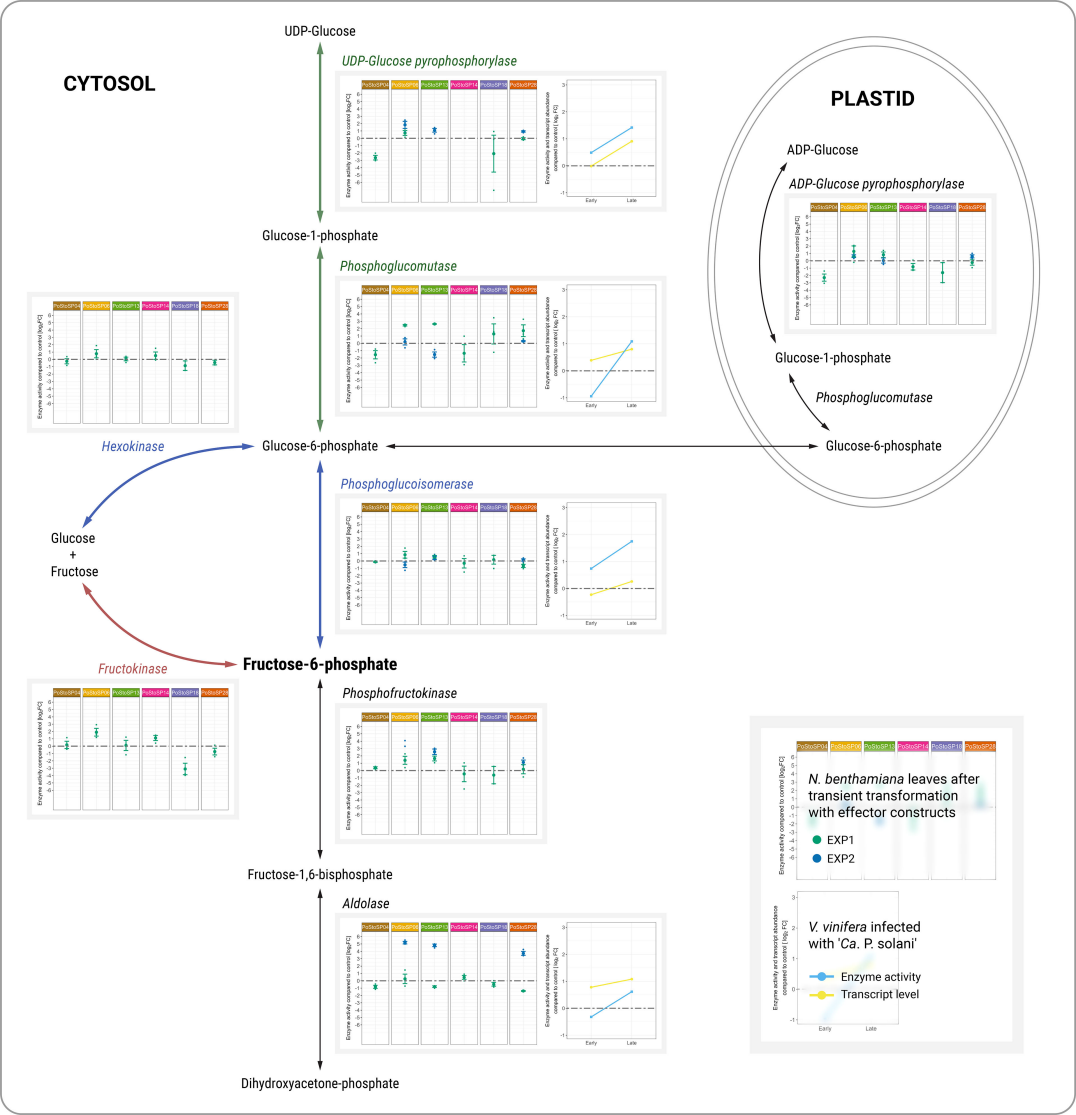
Activities of key enzymes of carbohydrate metabolism associated with sugar phosphorylation pathways. Enzyme activities from two independent experiments (green dot, EXP1; blue dot, EXP2) after transient transformation of *N. benthamiana* with different effector constructs in inoculated leaves 14 days after transformation compared with the control are shown in the left subpanels. Enzyme activities of grapevine along with transcript abundance of samples infected with ‘*Ca*. P. solani’ before (early) and after (late) symptom development compared to control samples are shown in the right subpanels. Transcript levels (Dermastia et al., 2021) correspond to the following genes: *Vitvi04g01633* for UDP-glucose pyrophosphorylase, *Vitvi01g00455* for phosphoglucomutase, *Vitvi18g00504* for phosphoglucoisomerase, and *Vitvi09g01500* for aldolase. Enzyme activity levels are shown as log_2_FC values; dashed gray line indicates no difference compared to the control.

**Figure 2:**
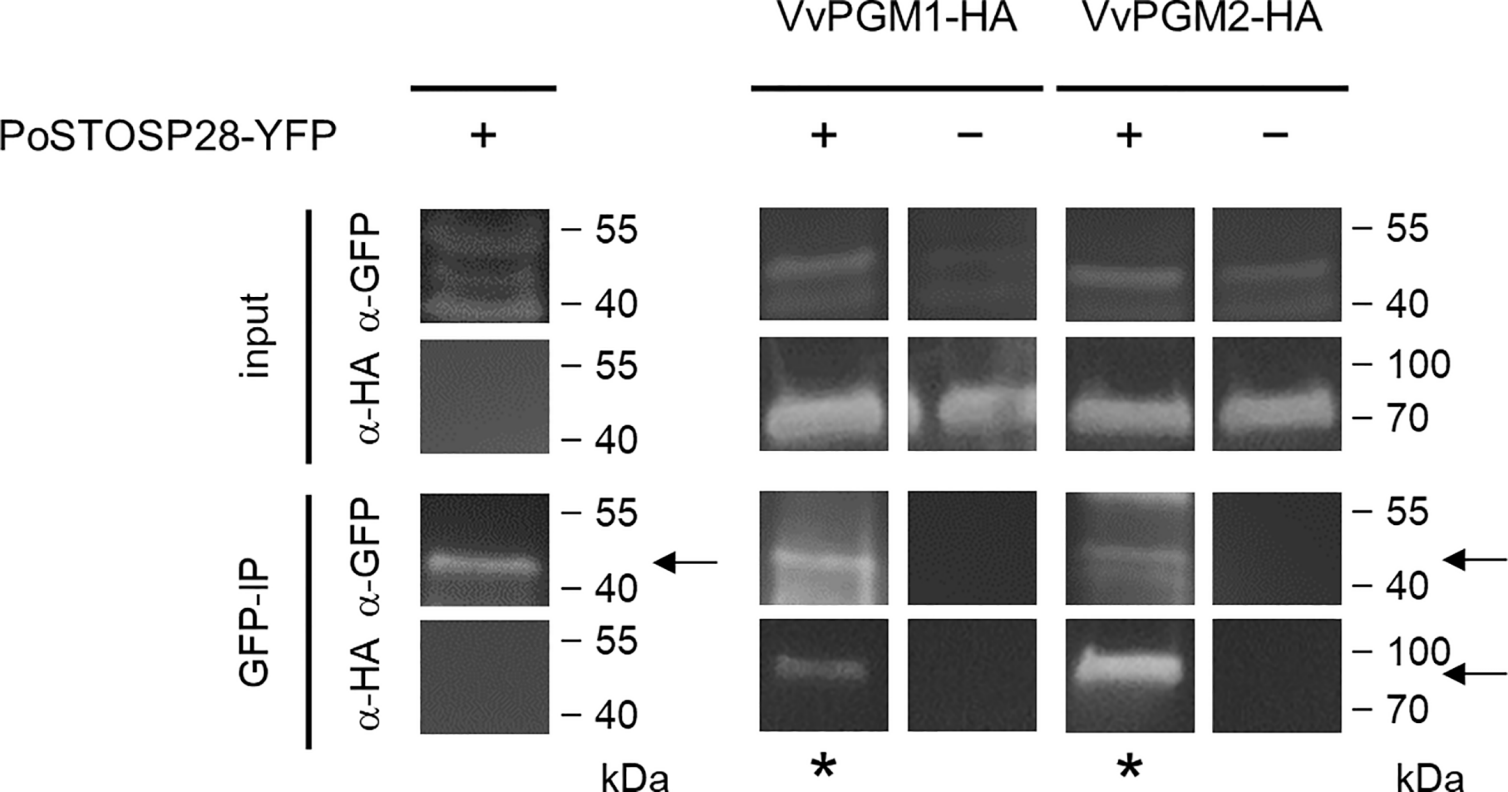
PoStoSP28 interacts with grapevine phosphoglucomutases *in planta*. Co-immunoprecipitation assay results, showing the interaction between PoStoSP28 and VvPGM1 and VvPGM2. The combination of YFP- and HA-tagged proteins expressed in *N. benthamiana* is indicated for each sample (+/−). Positive interactions were determined by detection of immunoprecipitated (GFP-IP) complexes with anti-HA antibodies (asterisks). Detection of YFP-tagged PoStoSP28 with anti-GFP antibodies in GFP-IP samples and detection of proteins with anti-GFP and anti-HA antibodies in leaf protein extracts (input) are shown as controls. Negative controls for GFP are shown in **Supplemental Figure S3**. The arrows indicate the expected bands.

Difference in enzyme activity levels (**Supplemental Table S2**) in agroinfiltrated and systemic leaves, 3 and 14 days after agroinfiltration, between treatment (candidate effector) and a control group (RNA p19 silencing suppressor) was examined using permutation Welch two-sample t-test (**Supplemental Figure S1**). Furthermore, to visualize the ratio of enzyme activity levels of transformed *N. benthamiana* compared with that of the control across two independent experiments, raw values were log_2_-transformed and scaled according to the average of log_2_-transformed values of the corresponding control. Summary statistics was calculated on scaled data. Log_2_ fold-change values, mean, and standard errors of the mean are shown in **Figures 1**, **2**. An equivalent procedure was conducted for ‘*Ca*. P. solani’–infected *V. vinifera* enzyme activity levels. Log_2_ fold-change values of enzyme activity and transcript abundance are shown in **Figures 1**, **2**. Analysis and visualization was conducted using R (Wickham, 2016) and several R packages (Graves et al., 2019. multcompView: Visualizations of Paired Comparisons. R package version 0.1-8; Auguie B. 2017. gridExtra: Miscellaneous Functions for “Grid” Graphics. R package version 2.3; Neuwirth E. 2022. RColorBrewer: ColorBrewer Palettes. R package version 1.1-3; Patil, 2023. broomExtra: Enhancements for “broom” and “easystats” Package Families; Kohl M. 2023. MKinfer: Inferential Statistics_R package version 1.0).

## 3 Results

### 3.1 Prediction of candidate pathogenicity factor/effector proteins

DNA sequences of six Austrian genotypes covering both stinging nettle and bindweed-associated strains of ‘*Ca.* P. solani’ together with a SA-1 strain of ‘*Ca.* P. solani’ from bindweed (Seruga Music et al., 2019) (**Table 2**) revealed the conserved presence of signal peptide cleavage sites that are characteristic of candidate effector proteins, as well as the presence of transmembrane helices (as effector or membrane-bound and plant cell-exposed protein) (**Tables 1**, **2**). Six of these proteins, which corresponded to locus tags PSSA1_RS01075, PSSA1_RS02495 PSSA1_RS03585, PSSA1_RS03580, PSSA1_RS01645, and PSSA1_RS01265 in ‘*Ca*. P. solani’ strain SA-1 (Seruga Music et al., 2019), were found in six different genotypes CPsM4_At1, CPsM4_At4, CPsM4_At6, CPsM4_At7, CPsM4_At10, and CPsM4_At12 and fulfilled the criteria of signal peptide cleavage sites. The presence of transmembrane helices and expression *in planta* detected by RNA sequencing were selected as additional criteria for putative pathogenicity factor/effector proteins PoStoSP04, PoStoSP06, PoStoSP13, PoStoSP14, PoStoSP18, and PoStoSP28 (**Tables 1**, **2**). The sequences are deposited in NCBI under accession numbers OX10001- OX10036, KJ469716, KJ469719, KJ469721, and KJ469722.

### 3.2 PoStoSP18 and PoStoSP28 have known functions

Although the genomic sequences of the putative effectors were revealed within the framework of this study according to the predicted effector criteria, i.e., the presence and location of signal peptide cleavage sites and transmembrane helices, two of them have been previously annotated as functional genes. According to the inferred protein sequence, PoStoSP18 was annotated as coding for a maltose-binding protein (malE) within the ATP-binding cassette (ABC)–type sugar transport system (A0A421NXH1) (**Tables 1**, **2**). PoStoSP28, on the other hand, is 86%–100% identical to an antigenic membrane protein StAMP from ‘*Ca.* P. solani’ (Fabre et al., 2011), (**Tables 1**, **2**).

### 3.3 Attempts to decipher the mechanisms used by ‘*Ca*. P. solani’ to manipulate carbohydrate metabolism: determination of enzyme activity signatures

Assuming that ‘*Ca*. P. solani’ effectors are directly involved in the altered carbohydrate metabolism previously demonstrated for ‘*Ca*. P. solani’ infection, 12 key enzymes of carbohydrate metabolism (i.e., ADP-glucose pyrophosphorylase, phosphoglucomutase, aldolase, phosphofructokinase, phosphoglucoisomerase, hexokinase, fructokinase, glucose-6-phosphate dehydrogenase, UDP-glucose pyrophosphorylase, cytoplasmic invertase, cell wall invertase, and vacuolar invertase) were tested in leaves of *N. benthamiana* after *Agrobacterium*-mediated transient transformation with each of chosen effector constructs originated from the strain VvKn_Zw2 (**Table 2**). Enzyme activity was determined in a leaf agroinfiltrated with the effector construct and in the leaf above (systemic leaf), 3 and 14 days after agroinfiltration (**Supplemental Table S2**, Nicotiana; **Supplemental Figure S1**) by a semi–high-throughput analytical platform (Jammer et al., 2022) that integrates the different levels of regulatory mechanisms. Already 3 days after agroinfiltration, changes in the activities of some analyzed enzymes were detected in some agroinfiltrated and systemic leaves (**Supplemental Table S2**, Nicotiana; **Supplemental Figure S1**). Compared with control leaves, some changes were also detected in systemic leaves 14 days after inoculation (**Supplemental Table S2**, Nicotiana; **Supplemental Figure S1**). However, the differences in enzyme activities were consistent in leaves inoculated with the effector constructs 14 days after inoculation compared with control leaves, which was in accordance with the transcript levels and the activities of the corresponding enzymes in symptomatic leaves of grapevines cv. ‘Zweigelt’ late in the growing season (**Figure 1**; **Supplemental Table S2**, Vitis; **Supplemental Figure S2**).

Increased activity of UDP-glucose pyrophosphorylase was associated with PoStoSP06 and PoStoSP13. PoStoSP18 and PoStoSP28 (StAMP) affected phosphoglucomutase activity, whereas PoStoSP04, PoStoSP06, PoStoSP13, and PoStoSP28 (StAMP), to a certain degree, affected phosphoglucomutase activity phosphofructokinase activity. Aldolase was affected by PoStoSP06, PoStoSP13, and PoStoSP28. In addition, increased activity of hexokinase and fructokinase was associated with PoStoSP06, PoStoSP13, and PoStoSP14 and PoStoSP04, PoStoSP06, PoStoSP13, and PoStoSP14, respectively, compared with the control. A minor effect on ADP-glucose pyrophosphorylase activity was detected in *N. benthamiana* leaves inoculated with the effector constructs of effectors PoStoSP06 and PoStoSP13 (**Figure 1**).

In symptomatic grapevines cv. ‘Zweigelt’ infected with ‘*Ca.* P. solani’ compared with uninfected grapevines, metabolic pathways associated with phosphorylated sugar production were induced both at the transcriptional level (i.e., *Vitvi04g01633* coding for UDP-glucose pyrophosphorylase, *Vitvi01g00455* coding for phosphoglucomutase, *Vitvi18g00504* coding for phosphoglucoisomerase, *Vitvi14g01938* coding for phosphofructokinase, and *Vitvi09g01500* coding for aldolase) (Dermastia et al., 2021) and at the level of activity of the corresponding enzymes (**Figure 1**; **Supplemental Table S2**, Vitis; **Supplemental Figure S2**).

### 3.4 PoStoSP28 directly interacts with grapevine phosphoglucomutase enzyme proteins

We used a pull-down assay to detect potential physical interaction(s) between PoStoSP28 and *N. benthamiana* proteins. We identified potential interactors from 58 protein groups. These proteins were involved in various processes, including primary and secondary metabolism, redox regulation, photosynthesis, protein degradation, and processing (**Supplemental Table S3** and PRIDE database) (Perez-Riverol et al., 2019). On the basis of our previous analysis of grapevines infected with ‘*Ca.* P. solani’ (Hren et al., 2009) and the results from this study, we selected phosphoglucomutase (PGM, UniProtKB identifiers A0A1S3XHG4, A0A1S4AIH6, and A0A1S4A6S4), which was associated with increased enzyme activity in *N. benthamiana* leaves after transient transformation with PoStoSP28 (**Figure 1**), for further analysis. Using an *in planta* co-IP assay of YFP-tagged PoStoSP28 and HA-tagged *N. tabacum* phosphoglucomutase orthologs in grapevine *Vitvi16g00891* (VvPGM1) and *Vitvi01g00455* (VvPGM2) in *N. benthamiana* (**Figure 2**), we confirmed that PoStoSP28 interacts with both grapevine phosphoglucomutases.

### 3.5 Association of ‘*Ca.* P. solani’ infection with oxidative stress

**Figure 3:**
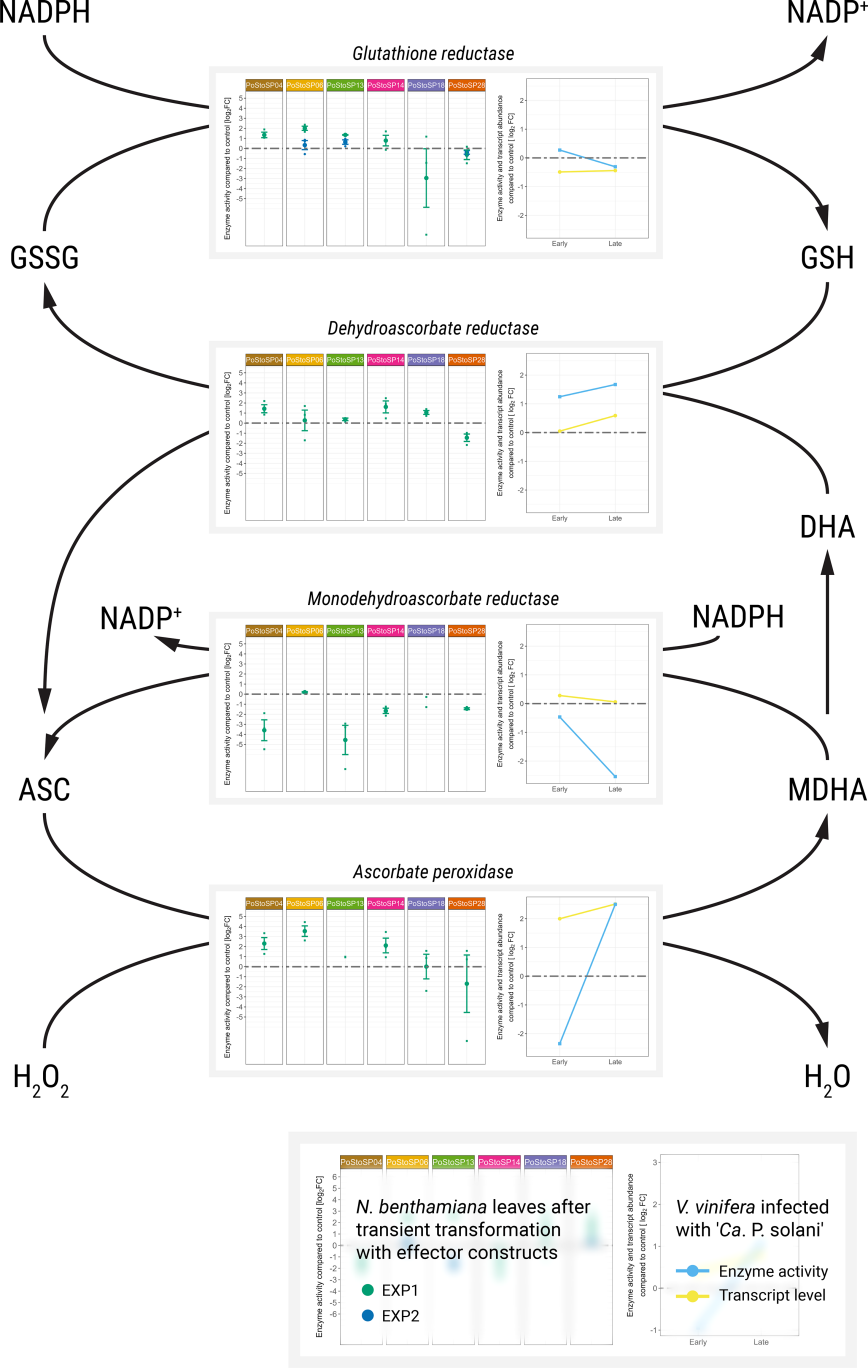
Activities of key enzymes involved in the ascorbate–glutathione cycle. Enzyme activities after transient transformation of *N. benthamiana* with different effector constructs in the agroinfiltrated leaves 14 days after transformation compared with the control leaves are shown in the left subpanels. Enzyme activities of grapevine along with transcript abundance of samples infected with ‘*Ca*. P. solani’ before (early) and after (late) symptom development compared to the control sample are shown in the right subpanels. Transcript levels (Dermastia et al., 2021) correspond to the following genes: *Vitvi04g02166* for ascorbate peroxidase, *Vitvi08g01483* for monodehydroascorbate reductase, *Vitvi13g00241* for dehydroascorbate reductase, and *Vitvi07g00037* for glutathione reductase. Enzyme activity levels are shown as log_2_FC values; dashed gray line indicates no difference compared to the control.

Transient transformation of *N. benthamiana* leaves with candidate effector constructs affects the activity of enzymes involved in oxidative stress (i.e., ascorbate peroxidase, catalase, dehydroascorbate reductase, glutathione reductase, monodehydroascorbate reductase, peroxidase, apoplastic peroxidases, and superoxide dismutase) as early as 3 days after agroinfiltration in both inoculated and systemic leaves (**Supplemental Table S2**, Nicotiana; **Supplemental Figure S1**). Similar to enzymes related to carbohydrate metabolism, more consistent changes in enzyme activity were observed in inoculated leaves compared with that in control leaves 14 days after agroinfiltration (**Figure 3**; **Supplemental Table S2**, Vitis; **Supplemental Figure S2**). At this time, transformation with the candidate effectors PoStoSP04, PoStoSP06, PoStoSP13, and PoStoSP04 was associated with an increase in ascorbate peroxidase activity, which was also strongly induced in grapevine cv. ‘Zweigelt’ infected with ‘*Ca*. P. solani’ late in the growing season (**Figure 3**). Interestingly, the activity of monodehydroascorbate reductase after agroinfiltration with all candidate effectors was lower than that in the control and comparable with the control after transformation with PoStoSP06. In addition, there was a decrease in the transcript of the two grapevine monodehydroascorbate reductase genes *Vitvi08g01483* and Vitvi14g01751 (**Figure 3**) and in the activity of the corresponding enzyme late in the growing season (**Figure 3**). A transcript of *Vitvi13g00241* (**Figure 3**), along with the activity of the associated dehydroascorbate reductase, was increased in grapevine late in the growing season, and this induction was consistent with increased activity in *N. benthamiana* leaves after transformation by all effectors tested, with the exception of PoStoSP28 (**Figure 3**). Enzyme activity for glutathione reductase was higher after transformation with all effector constructs except PoStoSP18 and PoStoSP28 compared with control (**Figure 3**). In symptomatic grapevines late in the growing season, the transcript level of *Vitvi07g00037* encoding glutathione reductase was slightly lower compared with uninfected grapevines (**Figure 3**), but the activity of the corresponding enzyme remained similar (**Figure 3**). In addition, the activity of *N. benthamiana* catalase was slightly increased by all effector constructs tested, similar to the activity in grapevine (**Supplemental Figure S4**).

### 3.6 Subcellular localization of the candidate pathogenicity factor/effector proteins

**Figure 4:**
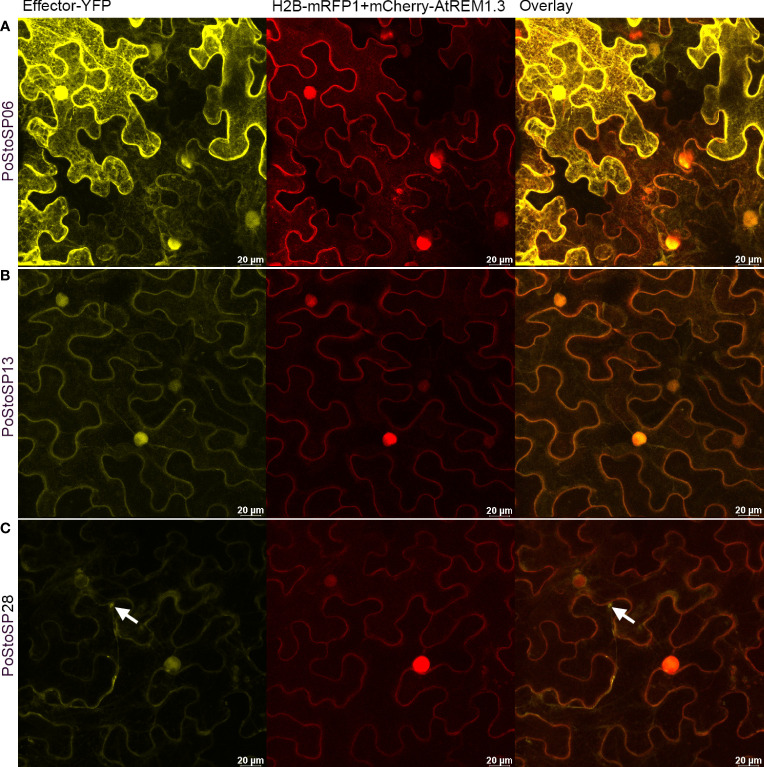
Subcellular localization of PoStoSP06 **(A)**, PoStoSP013 **(B)**, and PoStoSP28 **(C)**. From left to right: effector tagged with YFP (effector-YFP), nuclear marker histone H2B tagged with mRFP (H2B_RFP), and plasmalemma marker *A thaliana* remorin 1.3 tagged with mCherry (mCherry-AtREM1.3), overlay of pathogenicity factor/effector and organelle markers. White arrow indicates autophagosome-like structure. Scale bar, 20 µm.

To determine the subcellular localization of pathogenicity factor**/**effector proteins, particularly those with the greatest impact on enzymes involved in the phosphorylation of sugars (PoStoSP06, PoStoSP013, and PoStoSP28), *Agrobacterium*-mediated transient transformation of *N. benthamiana* with YFP-tagged pathogenicity factor**/**effector sequences was performed. Their expression showed that all the proteins examined were localized in the nucleus and cytosol (**Figure 4**; **Supplemental Figure S5**). Of all the pathogenicity factor**/**effector proteins, PoStoSP28 showed the highest expression in the thread-like structures spanning the cells (**Figure 4**; **Supplemental Figure S5**). Although it was also present in the nucleus, its expression was generally lower compared with the other pathogenicity factor**/**effectors and accompanied by a distinct border at the periphery or just outside the nucleus (**Figure 4**; **Supplemental Figure S5**).

### 3.7 Pathogenicity factor PoStoSP28 induces autophagosomes in *N. benthamiana*

**Figure 5:**
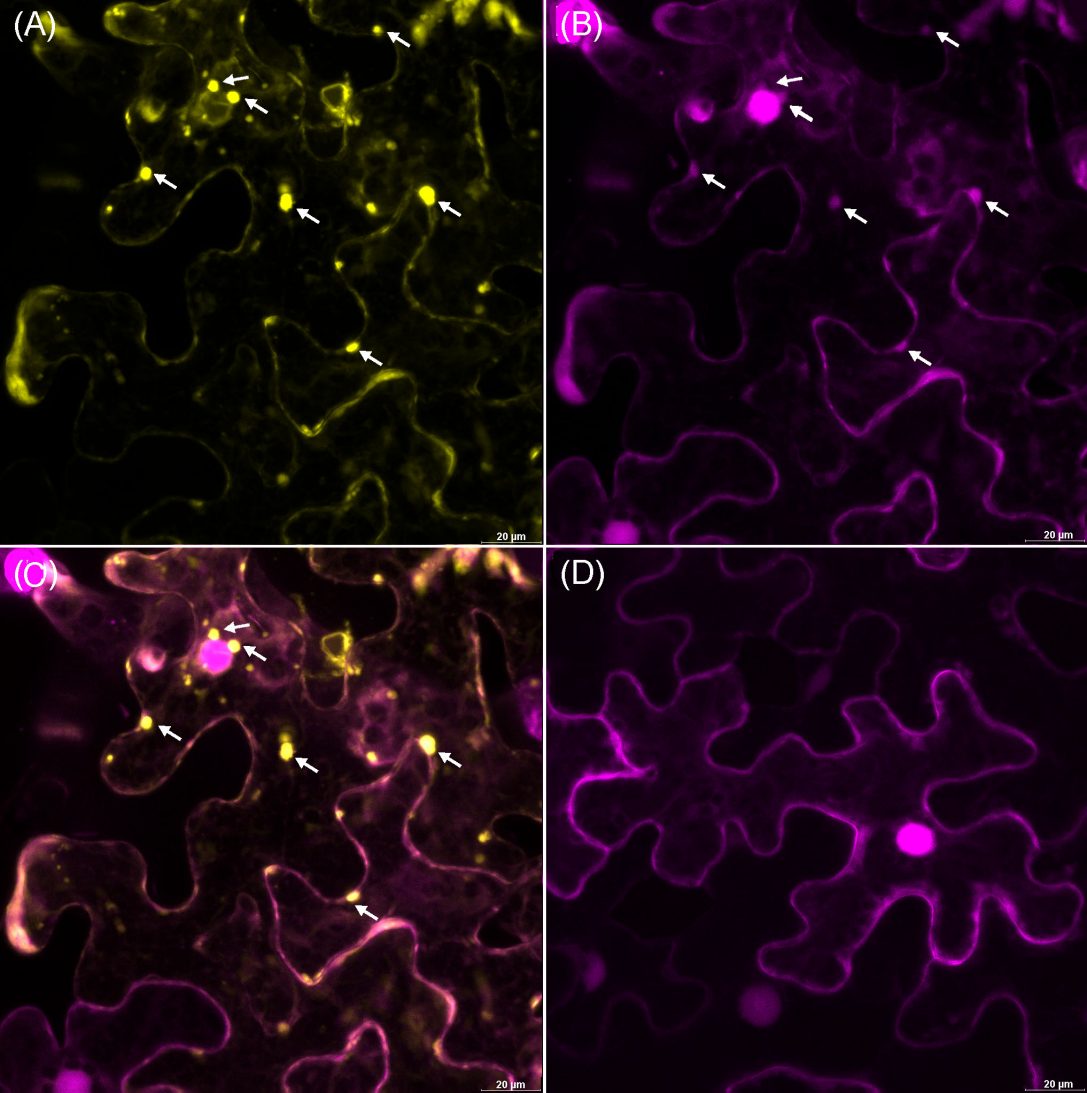
The potential pathogenicity factor PoStoSP28 induces autophagosome formation in inoculated cells. The YFP-tagged effector PoStoSP28 co-expressed with the mRFP1-tagged ATG8 CL autophagosome marker. White arrows indicate co-localization of PoStoSP28 and autophagosome markers. **(A)** YFP fluorescence of YFP-tagged PoStoSP28 (yellow), **(B)** mRFP1-tagged nuclear marker histone H2B and mRFP1-tagged ATG8 CL marker fluorescence (magenta), **(C)** overlay of images, and **(D)** control leaf; cell expressing only mRFP1-tagged ATG8 CL. Scale bar, 20 µm.

Upon closer inspection, some autophagosome-like structures were found in *N. benthamiana* cells expressing the PoStoSP28 effector (**Figure 4**), reminiscent of the structures described as autophagosomes by (Dagdas et al., 2016). To identify these structures, the pathogenicity factor of interest was expressed along with the RFP-tagged ATG8 CL autophagosome marker. The marker showed that the observed structures were co-localized with autophagosomes (**Figure 5**). Moreover, almost no autophagosomes were observed in the control plants expressing only the RFP-tagged ATG8 CL autophagosome marker (**Figure 5D**), suggesting that the pathogenicity factor PoStoSP28 not only is localized in the autophagosome but can also increase the occurrence of autophagosomes.

## 4 Discussion

### 4.1 Candidate pathogenicity factors/effectors show some phytoplasma strain specificity

Candidate effectors from the ‘*Ca*. P. solani’ genotype CPsM4_At6 originating from the grapevine cv. ‘Zweigelt’ were compared with effectors from the ‘*Ca*. P. solani’ strain SA-1, which is originated from infected grapevines and was transferred to and maintained in Madagascar periwinkle (Seruga Music et al., 2019). PoStoSP04 is identical to PSSA1_RS010758, whereas the other candidates have up to 85% identity at the protein level. Interestingly, the most significant differences were found between genotypes associated with stinging nettle (CPsM4_At1 and CPsM4_At4) and those associated with bindweed (CPsM4_At6, CPsM4_At7, CPsM4_At10, and CPsM4_At12). Given a broad plant host range and the polyphagous insect vectors of ‘*Ca*. P. solani’, the presence of a considerable number of epidemiological or strain-specific genes within the species has already been suggested (Seruga Music et al., 2019), indicating a possible differentiation in phenotype expression and host adaptation.

PoStoSP18 and PoStoSP28 are not included in the effector list of the strain SA-1 (Seruga Music et al., 2019), but they can be found in its genome (genome accession number: NZ_MPBG00000000). They also contain signal peptide sequences and are exposed to the plant cell in the ‘*Ca*. P. solani’ genome, with a putative function in phytoplasma-insect interaction (Fabre et al., 2011). The gene *stamp* is highly variable, as shown in this and several other studies on grapevines infected with ‘*Ca.* P. solani’ from different countries (Cvrković et al., 2014; Atanasova et al., 2015; Murolo and Romanazzi, 2015; Delić et al., 2016; Quaglino et al., 2021; Mehle et al., 2022). This variability suggests that StAMP is subject to positive diversifying selection pressure, likely related to its interaction with the insect vector (Fabre et al., 2011). StAMP has a similar structure to the cell-surface phytoplasma antigen membrane proteins (AMPs) of ‘*Ca.* P. asteris’, which form a complex with microfilaments of insect cells only in the viscera and salivary glands of insect vectors (Suzuki et al., 2006; Galetto et al., 2011). A similar structure might indicate a similar function as AMP of ‘*Ca.* P. asteris’ (Kakizawa et al., 2004). Because phytoplasma can survive only in their insect vectors or in the phloem of their host plants, the possible biological role of StAMP as a plant cell exposed pathogenicity factor in plant pathogenesis was investigated in this study along with other identified candidate effectors.

On the other hand, several substrate-binding proteins associated with the ABC transporters and secreted via the Sec-dependent pathway have been characterized in ‘*Ca*. P. asteris’ strain AY-WB (Bai et al., 2009) and are also present in ‘*Ca*. P. solani’strain SA-1 (Seruga Music et al., 2019). In this study, we investigated a putative substrate-binding protein of the ABC-type sugar transport system annotated as MalE (A0A421NXH1) as the candidate effector PoStoSP18. MalE is a maltose-binding protein encoded in phytoplasma genomes, but it can also bind other sugars (Stülke et al., 2009).

Phytoplasma genomes include potential mobile units, putative transposon-like elements that are thought to contribute to the genome instability observed in these bacteria (Bai et al., 2009). They are also present in the ‘*Ca*. P. solani’ strain SA-1 genome (Seruga Music et al., 2019). These potential mobile units and DNA elements often contain genes for candidate virulence protein effectors (Bai et al., 2009). At least 20 of the proteins, annotated as putative effectors in ‘*Ca*. P. solani’ strain SA-1, are located in these regions (Seruga Music et al., 2019). However, a comparison of the candidate effectors studied in this work with the putative effectors from strain SA-1 indicates that only PoStoSP14, which can be detected in fragments in SA-1 on two contigs as PSSA1_RS03580 and PSSA1_RS02680, could be associated with a potential mobile that has been disrupted in this strain.

### 4.2 Modulation of phosphorylated sugars by candidate effectors

There is increasing evidence that infection by ‘*Ca.* P. solani’ affects carbohydrate metabolism in grapevine (Rotter et al., 2018; Škrlj et al., 2021; Dermastia et al., 2021), likely resulting in symptoms such as leaf curling; yellowing or reddening of leaf margins; and hard, brittle texture of leaves (Dermastia et al., 2017). However, it is not always easy to distinguish whether the primary event is the generation of a metabolic sugar signal or a direct effect of a corresponding pathogen signal (Prezelj et al., 2016a). By directly tracking the consequences of transient transformation of *N. benthamiana* with putative phytoplasma effectors on the host plant, this study partially solves a dilemma. Although the molecular mechanism behind the effect of the analyzed effector candidates on plant carbohydrate metabolism remains unclear, the localization of the effector in plant nuclei suggests a possible perturbation of this metabolism at transcriptional level, apart from direct interactions with enzymes, as shown for phosphoglucomutase.

The general upper part of glycolysis is encoded in all known phytoplasma genomes. The genes encoding the upper part of glycolysis (i.e., phosphoglucose isomerase, phosphofructokinase, and aldolase) were found in two strains of ‘*Ca*. P. solani’ originating from tobacco as well as in SA-1 strain originating from grapevine (Mitrović et al., 2014; Seruga Music et al., 2019). However, phytoplasma lack the hexokinase and sugar-specific phosphotransferase systems to mediate the entry of phosphorylated hexose into glycolysis (Kube et al., 2012), which is the first step in the breakdown of glucose to produce energy in the form of ATP. Our metabolome analysis shows that the amount of fructose-6-phosphate and other hexose-6-phosphates in the middle leaf veins, containing phloem sap of grapevines infected with ‘*Ca.*P. solani’ is 2.7- to 2.9-fold higher than in uninfected grapevines (Prezelj et al., 2016b). This result confirms the suggestion that there must be an as yet unclear phytoplasma pathway for importing sugars and generating hexose-6-phosphate to support glycolysis (Tan et al., 2021). For the first time, the results of this study show that phytoplasma candidate pathogenicity factors/effector proteins may be involved in the manipulation of plant metabolism by these processes. Moreover, they suggest that pathogenicity factors/effectors may even mediate more than one metabolic pathway that could supply phytoplasma with phosphorylated hexoses. Although the molecular mechanism by means of which the candidate pathogenicity factors/effectors of ‘*Ca.* P. solani’ alter plant metabolism to increase the activity of specific enzymes in the glycolytic pathway remains unknown, it appears that coordinated action of multiple candidate effectors is involved. This is consistent with the finding of Marrero et al. (2020), namely, that several effectors of ‘*Ca.* P. asteris’ AY-WB have at least two interactions with host proteins and some of them with more than 240 host proteins.

The first possible pathway to supply fructose-6-phosphate to ‘*Ca*. P. solani’ involves activation of the grapevine hexokinase system by the candidate effectors (**Figure 1**, blue pathway) and further conversion of the resulting glucose-6-phosphate by phytoplasma encoded phosphoglucose isomerase (Kube et al., 2012; Seruga Music et al., 2019). Fructose can also be directly converted to fructose-6-phosphate by fructokinase, which was affected by all effector constructs except PoStoSP18 and PoStoSP28 (**Figure 1**, red pathway). Another alternative pathway leading to fructose-6-phosphate involves induction of the enzyme UDP-glucose pyrophosphorylase leading to glucose-1-phosphate, followed by conversion of the resulting glucose-1-phosphate to glucose-6-phosphate by phosphoglucomutase (**Figure 1**, green pathway). Its activity was induced in transformed *N. benthamiana* by PoStoSP18 and PoStoSP28 (**Figure 1**, green pathway), and the product could be a substrate for phosphoglucoisomerase or for ADP-glucose pyrophosphorylase involved in starch biosynthesis (**Figure 1**, black pathway). In addition, the results suggest that PoStoSP28 plays a role in the pathogenicity of phytoplasma in grapevine by interacting with grapevine phosphoglucomutase enzymes.

It is worth noting that, in this analysis, PoStoSP18, as MalE, a maltose-binding protein within the ABC-type sugar transport system (Stülke et al., 2009), was only associated with the increase in phosphoglucomutase activity (**Figure 1**, green pathway). However, a possible role played by PoStoSP18 in ‘*Ca*. P. solani’ pathogenesis requires further clarification.

The conversion of fructose-6-phosphate to fructose-1,6-bisphosphate phosphofructokinase and of fructose-1,6-bisphosphate to dihydroxyacetone phosphate by aldolase was induced by PoStoSP06 and PoStoSP13. The latter was also affected by PoStoSP28 and was significantly higher at the transcriptional level and in enzyme activity in infected grapevine, suggesting a possible regulation of its activity through transcriptional regulation.

### 4.3 Candidate effectors induce an ascorbate–glutathione cycle

Conditions exceeding the sensory or pathogen defense capacities of plant cells disrupt the balance between oxidants and antioxidants in favor of oxidants, resulting in disruption of redox signaling and control and/or molecular damage (Sies et al., 2017). As a result, plants generate large numbers of reactive oxygen species (ROS) in cells. To avoid metabolic disturbances, plants must have systems for ROS elimination. On the other hand, because ROS are a common feature of plant defenses, it should also be advantageous for any pathogen to be able to block the effects of ROS. The ascorbate–glutathione cycle is a major antioxidant system for efficient elimination of ROS and maintenance of cellular homeostasis. Remarkably, the induction of the complete ascorbate–glutathione cycle in infected ‘Zweigelt’ grapevines toward the end of the growing season correlated positively with the induced enzyme activities of ascorbate peroxidase, dehydroascorbate reductase, and glutathione reductase as well as the decrease in monodehydroascorbate reductase activity in *N. benthamiana* leaves after transient transformation with different candidate effectors. It appears that all effector candidates tested contributed to the induced activity of ascorbate peroxide after transient transformation of *N. benthamiana* leaves, with no contribution from PoStoSP18 and PoStoSP28. Interestingly, PoStoSP04, the only candidate effector identical to that found in strain SA-1 (Seruga Music et al., 2019), contributed only to oxidative stress-related enzymes.

### 4.4 Possible involvement of candidate effectors in induced autophagosome formation in ‘*Ca.* P. solani’ pathogenicity

An interesting observation in this study was the co-localization of autophagosomes with the effector candidate PoStoSP28, after transient transformation of *N. benthamiana* with effector constructs. The autophagosome-like structure have also been observed in mesophyll cells of tomato infected with potato purple top phytoplasma, and some of them have fused with misshapen chloroplasts (Wei et al., 2022; Junichi et al., 2023). The formation of these *de novo* double-membrane vesicles is part of an evolutionarily conserved degradation process of macroautophagy, required for the maintenance of cellular homeostasis. Plants use autophagosomes to engulf damaged organelles, non-functional proteins, and pathogens. The autophagosmes then fuse with the plant vacuole, where their contents are degraded (Wun et al., 2020). Plant pathogens have evolved ways to bypass or modulate plant autophagy and use it to their own advantage (Leary et al., 2018). Macroautophagy is divided into several steps involving different ATGs. Some of them initiate the process, whereas ATG9 is required for the final formation of autophagosomes (Feng and Klionsky, 2017). In ‘Zweigelt’ grapevine, several autophagosome-related genes were upregulated in plants infected with ‘*Ca.* P. solani’, especially late in the growth period (Dermastia et al., 2021). The increased transcript levels of Atg18 (i.e., *Vitvi07g00210*, *Vitvi14g00317*, *Vitvi11g00813*, and *Vitvi19g01985*) and *Vitvi03g00492*, which encodes ATG2, were noted. It has been suggested that the ATG2–ATG18 complex binds the preautophagosomal structure to endoplasmic reticulum (ER) to initiate expansion during autophagosome formation (Kotani et al., 2018). In addition, the upregulated Atg9 gene *Vitvi07g00580* may be involved in further autophagosome expansion (Feng and Klionsky, 2017). Transcriptome analysis also revealed upregulation of *Vitvi16g01309* and *Vitvi04g01617* that encode ATG13, which has been shown to bind ATG9 (Suzuki et al., 2015). The mechanism linking these data to autophagosome induction in transformed *N. benthamiana* leaves is not known. There are reports of mammalian systems in which impaired regulation of redox signaling can lead to autophagic activity that causes a range of diseases (Yun et al., 2020). However, because of the low activity of ascorbate–glutathione cycle enzymes in leaves of *N. benhtamiana* transformed with the PoStoSP28 construct, a link between autophagosome formation induced by PoStoSP28 and oxidative stress is less likely.

## 5 Conclusions

This is the first comprehensive study on the possible physiological role of candidate effectors or pathogenicity factors of the plant pathogen ‘*Ca.* P. solani’. A bioinformatic comparison of their sequences in the genome ‘*Ca.* P. solani’ strain SA-1 and phytoplasma isolated from infected grapevines revealed several candidates. One of them was identical, and others showed strain-specific variations. StAMP and MalE, already suspected to play a role in phytoplasma pathogenicity, were included in the list of proteins exposed to the plant cell. Because phytoplasma obtain most nutrients from the plant and therefore compete with the host for similar or identical nutrient substrates, a minor change in metabolism could significantly affect the outcome of pathogen-host interactions in providing nutrients, energy, and metabolites for successful replication and infection in plants. Following previous studies showing altered carbohydrate metabolism in plants upon phytoplasma infection, this study confirms the modulation of this metabolism by potential effectors. Moreover, most of the effectors tested contributed to the enzyme activities of the ascorbate–glutathione cycle. In addition, the effector previously shown to interact with insect vectors as StAMP was associated with the induction of autophagosomes in plant cells. These results confirm that phytoplasma interfere with plant metabolism and pave the way for further elucidation of the molecular mechanisms involved in these interactions.

## Data availability statement

The datasets presented in this study can be found in online repositories. The names of the repository/repositories and accession number(s) can be found in the article/**Supplementary Material**. Project accession: PRJEB42777. mRNA samples accessions: ERS5673290, ERS5673291, ERS5673292, ERS5673293, ERS5673294, ERS5673295, ERS5673296, ERS5673297, ERS5673298, ERS5673299, ERS5673300, ERS5673301, ERS5673302, ERS5673303, ERS5673304, ERS5673305, ERS5673306, ERS5673307, ERS5673308, ERS5673309, ERS5673310, ERS5673311. sRNA samples accessions: ERS5672105, ERS5672104, ERS5672103, ERS5672102, ERS5672101, ERS5672100, ERS5672099, ERS5672098, ERS5672097, ERS5672096, ERS5672095, ERS5672094, ERS5672093, ERS5672092, ERS5672091, ERS5672090.

## Author contributions

MD, GB, MPN, TR, and KG planned and designed the research. MP-N, ŠT, RS, TČ, TL, AC, GB, BD, BA, SW, CS, MR-B, TR, MZ, AK, and MD performed experiments, conducted fieldwork, analyzed and validated data, and visualized results. MD prepared the original draft, and MD, GB, MPN, ŠT, RS, TL, AC, TR, SW, WW, and KG reviewed and edited it. All authors have read and agreed to the published version of the manuscript.
