# Supplementary material for: Candidate pathogenicity factor/effector proteins of ‘Candidatus Phytoplasma solani’ modulate plant carbohydrate metabolism, accelerate the ascorbate–glutathione cycle, and induce autophagosomes
Source: Front Plant Sci. 2023 Aug 18;14:1232367. doi: 10.3389/fpls.2023.1232367 (PMC10471893; doi:10.3389/fpls.2023.1232367)

Supplemental Information

Title: Candidate pathogenicity factor/effector proteins of '*Candidatus* Phytoplasma solani' modulate plant carbohydrate metabolism, accelerate the ascorbate-glutathione cycle and induce autophagosomes

Authors: Marina Dermastia*, Špela Tomaž, Rebeka Strah, Tjaša Lukan, Anna Coll, Barbara Dušak, Barbara Anžič, Timotej Čepin, Stefanie Wienkoop, Aleš Kladnik, Maja Zagorščak, Monika Riedle-Bauer, Christina Schönhuber, Wolfram Weckwerth, Kristina Gruden, Thomas Roitsch, Maruša Pompe Novak, Günter Brader

* Correspondence: marina.dermastia@nib.si

Supplemental Figure S1. Activities of key enzymes (1) involved in carbohydrate metabolism associated with associated with sugar phosphorylation pathways and (2) involved in ascorbate-glutathione cycle. Enzyme activities after transient transformation of *N. benthamiana* with different effector constructs in agroinfiltrated leaves and in the leaf above, referred as the systemic leaf, 3 and 14 days after transformation compared to control were examined using permutation Welch two sample t-test. The results of two independent experiments (EXP1, EXP2) are shown.

ADP-glucose pyrophosphorylase

PoStoSP04 vs p19 (EXP1)

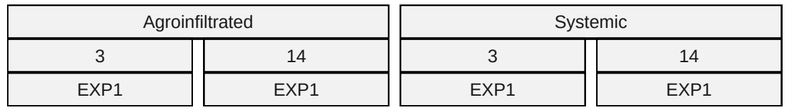

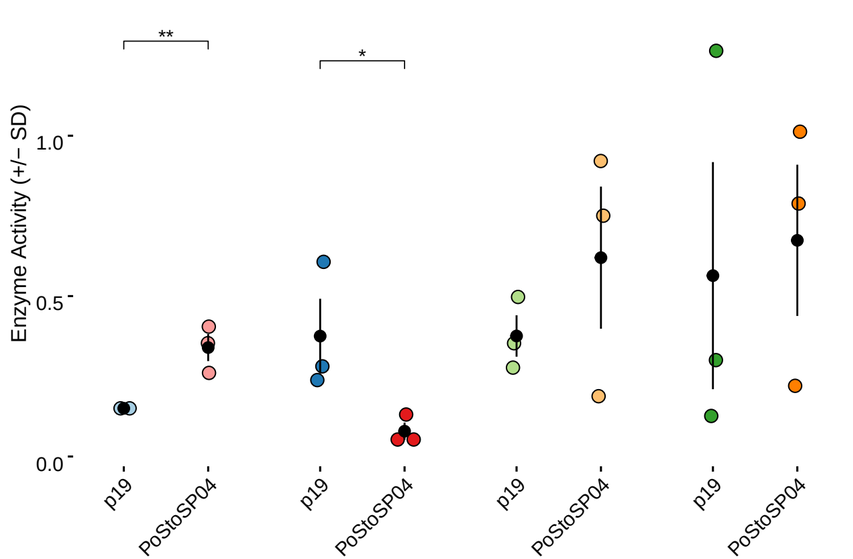

PoStoSP06 vs p19 (EXP1, EXP2)

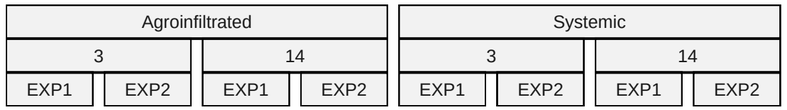

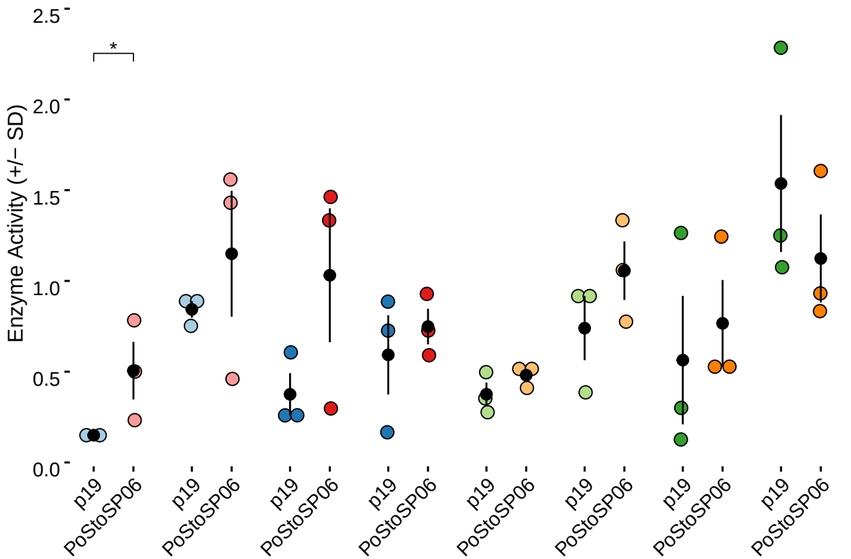

PoStoSP13 vs p19 (EXP1, EXP2)

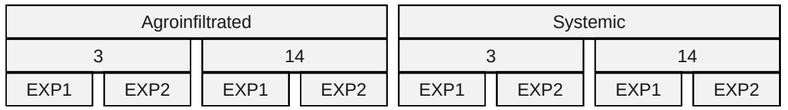

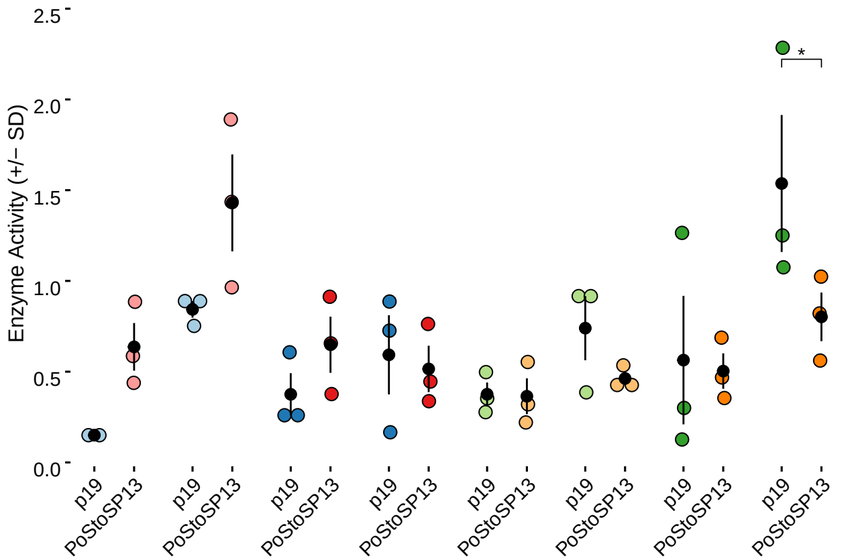

PoStoSP14 vs p19 (EXP1)

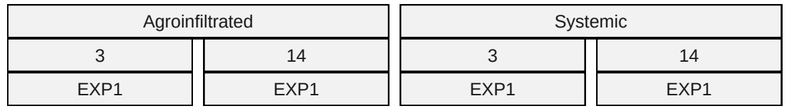

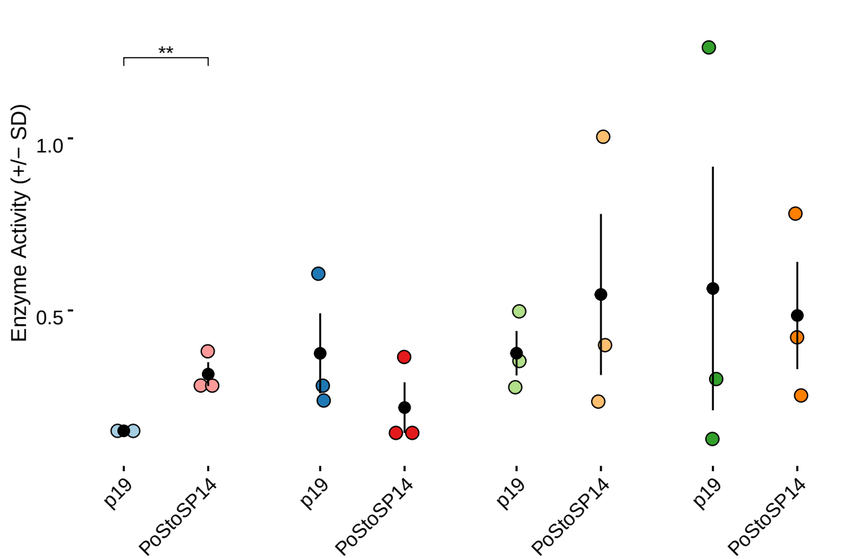

PoStoSP28 vs p19 (EXP1, EXP2)

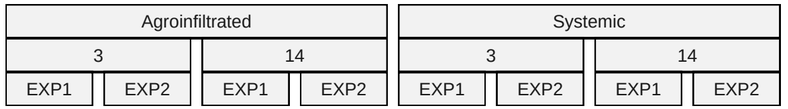

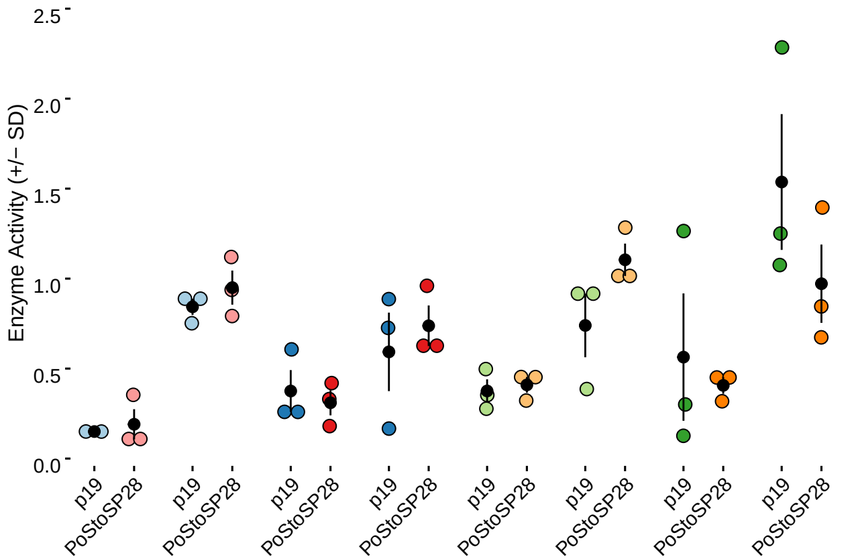

Aldolase

PoStoSP04 vs p19 (EXP1)

Agroinfiltrated		Systemic	
3	14	3	14
EXP1	EXP1	EXP1	EXP1

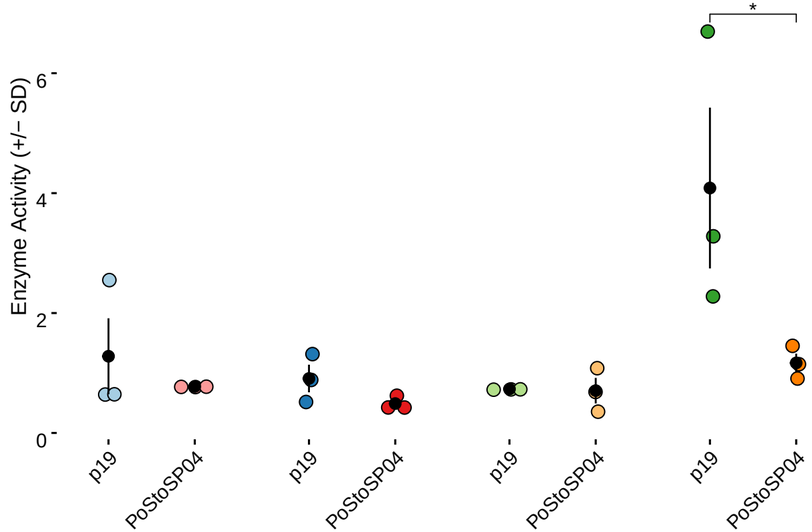

PoStoSP06 vs p19 (EXP1, EXP2)

Agroinfiltrated				Systemic			
3		14		3		14	
EXP1	EXP2	EXP1	EXP2	EXP1	EXP2	EXP1	EXP2

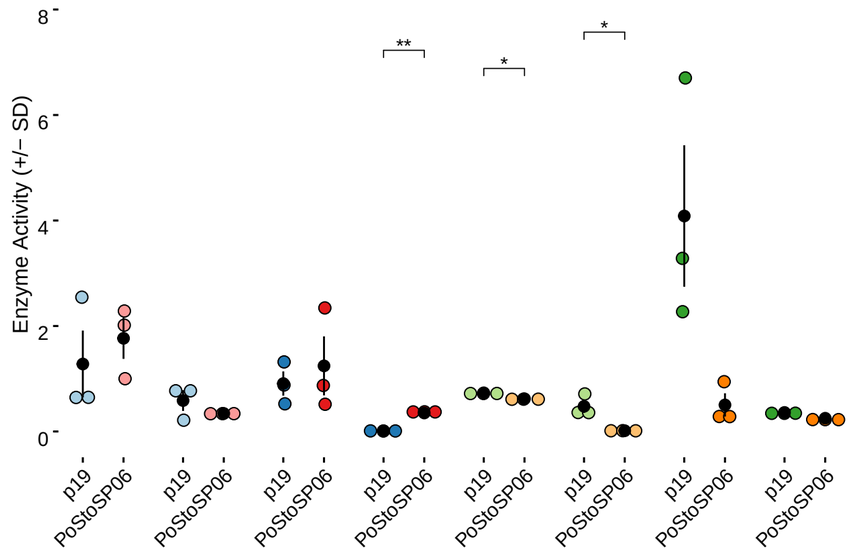

PoStoSP13 vs p19 (EXP1, EXP2)

Agroinfiltrated				Systemic			
3		14		3		14	
EXP1	EXP2	EXP1	EXP2	EXP1	EXP2	EXP1	EXP2

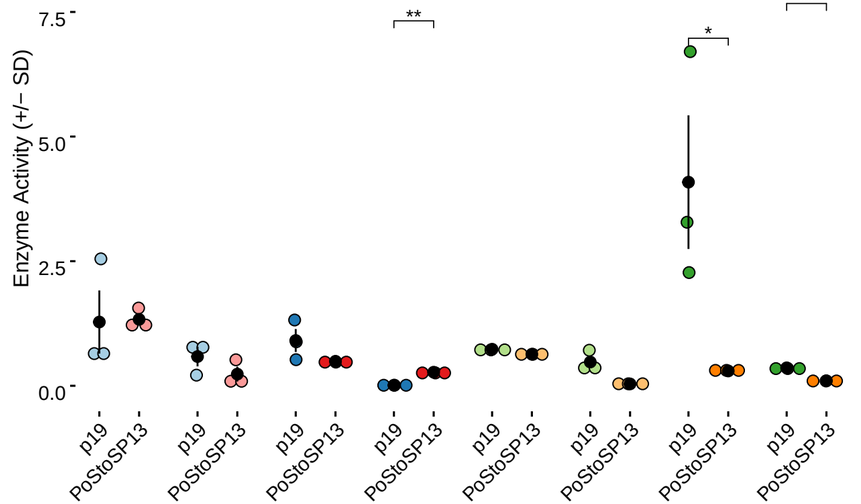

PoStoSP14 vs p19 (EXP1)

Agroinfiltrated		Systemic	
3	14	3	14
EXP1	EXP1	EXP1	EXP1

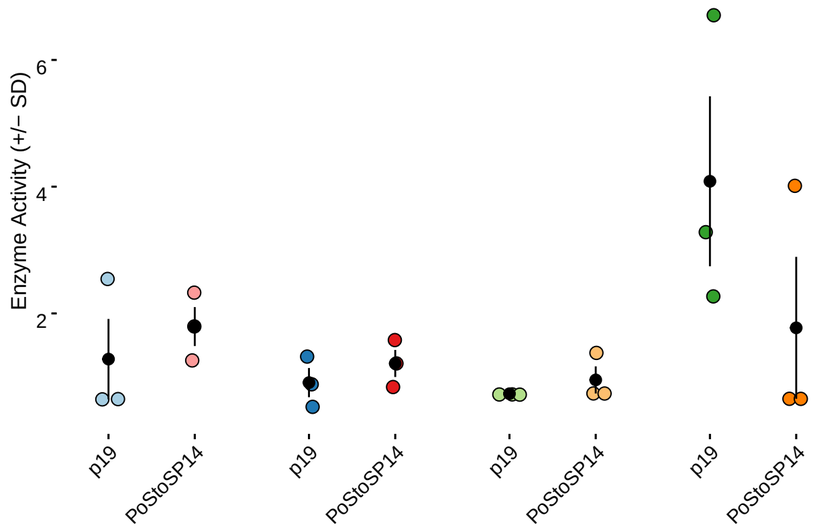

PoStoSP18 vs p19 (EXP1)

Agroinfiltrated		Systemic	
3	14	3	14
EXP1	EXP1	EXP1	EXP1

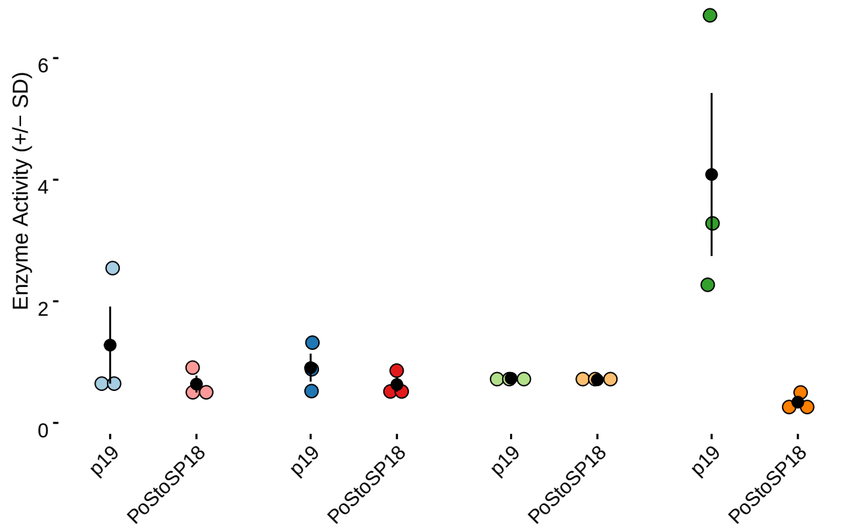

PoStoSP28 vs p19 (EXP1, EXP2)

Agroinfiltrated				Systemic			
3		14		3		14	
EXP1	EXP2	EXP1	EXP2	EXP1	EXP2	EXP1	EXP2

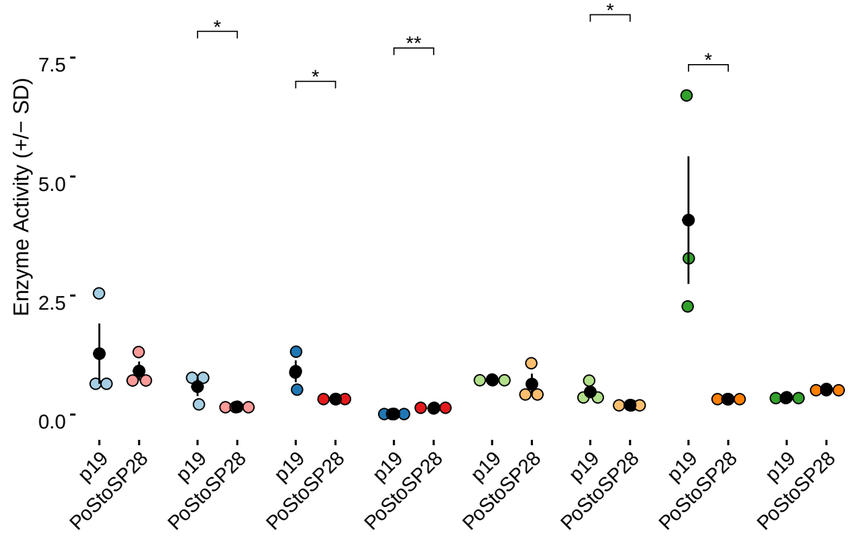

Fructokinase

PoStoSP04 vs p19 (EXP1)

Agroinfiltrated		Systemic	
3	14	3	14
EXP1	EXP1	EXP1	EXP1

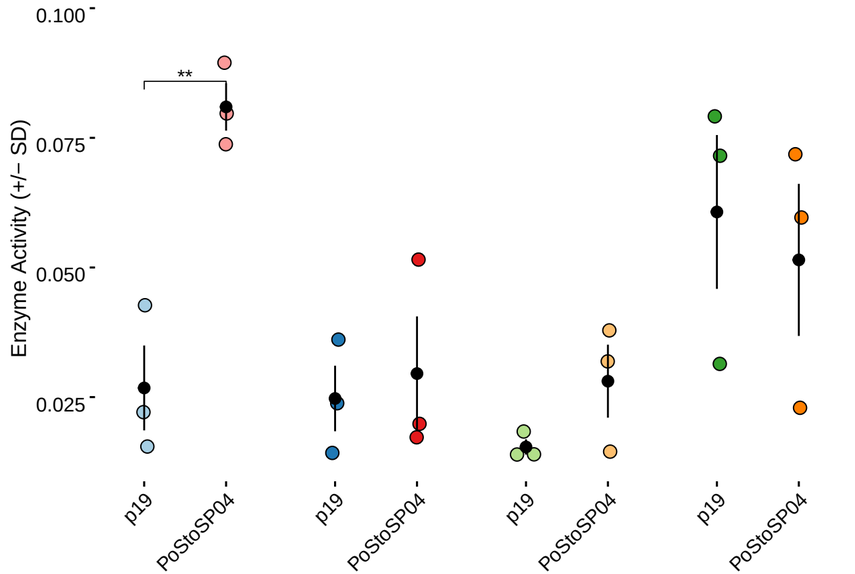

PoStoSP06 vs p19 (EXP1)

Agroinfiltrated		Systemic	
3	14	3	14
EXP1	EXP1	EXP1	EXP1

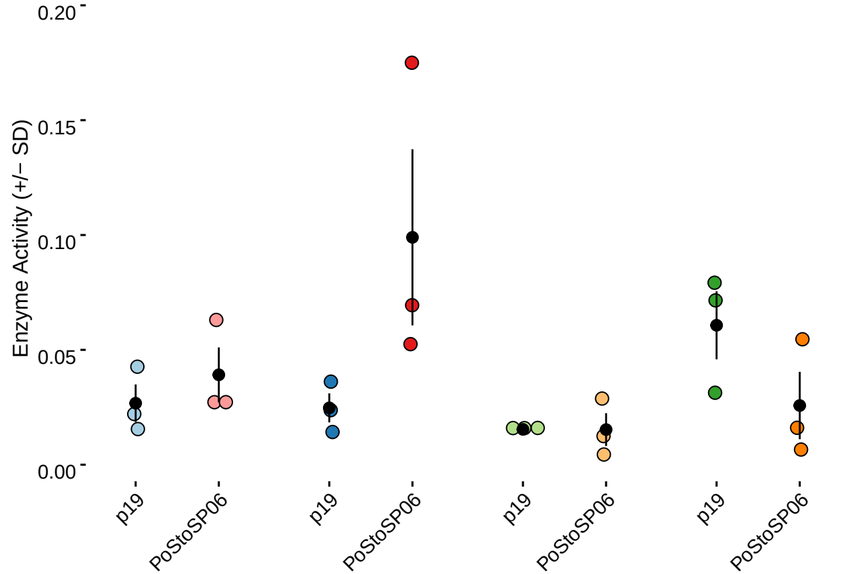

PoStoSP13 vs p19 (EXP1)

Agroinfiltrated		Systemic	
3	14	3	14
EXP1	EXP1	EXP1	EXP1

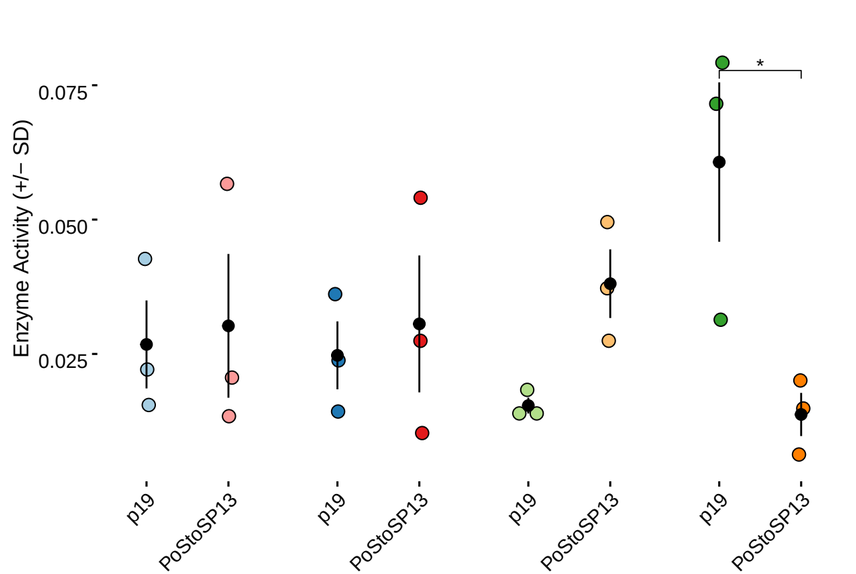

PoStoSP14 vs p19 (EXP1)

Agroinfiltrated		Systemic	
3	14	3	14
EXP1	EXP1	EXP1	EXP1

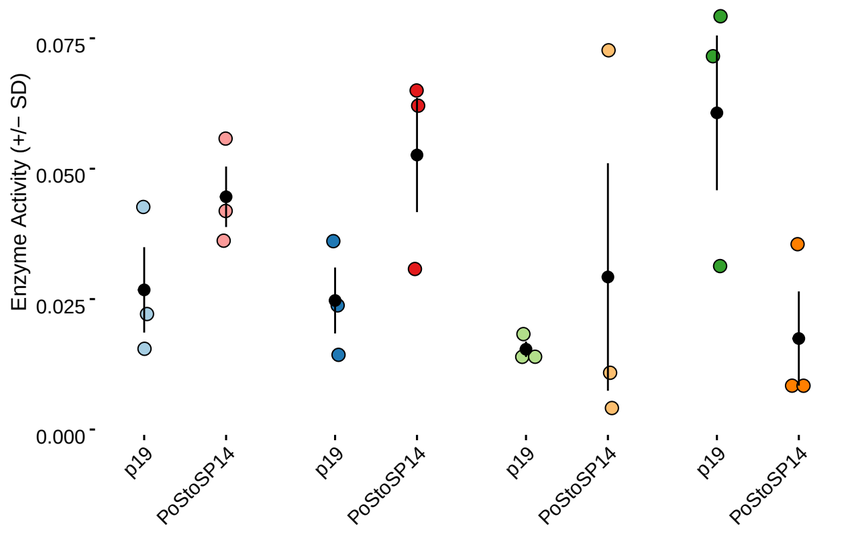

PoStoSP18 vs p19 (EXP1)

Agroinfiltrated		Systemic	
3	14	3	14
EXP1	EXP1	EXP1	EXP1

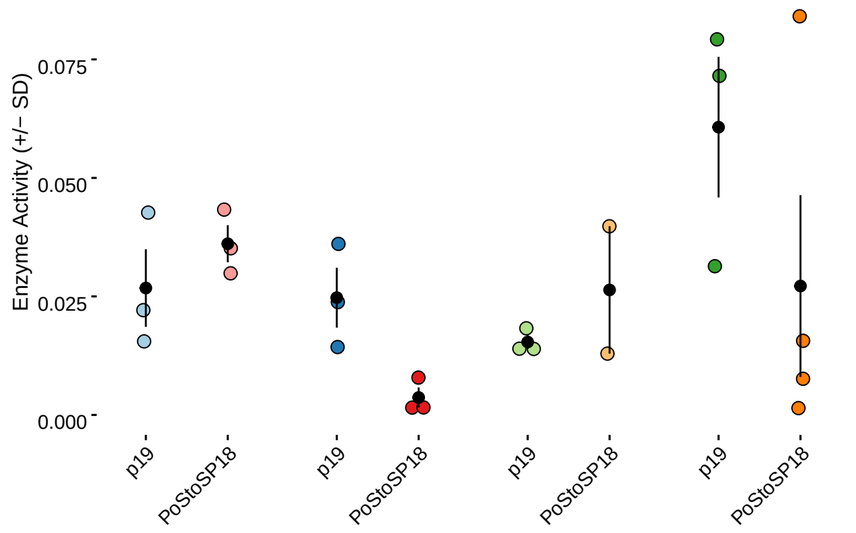

PoStoSP28 vs p19 (EXP1)

Agroinfiltrated		Systemic	
3	14	3	14
EXP1	EXP1	EXP1	EXP1

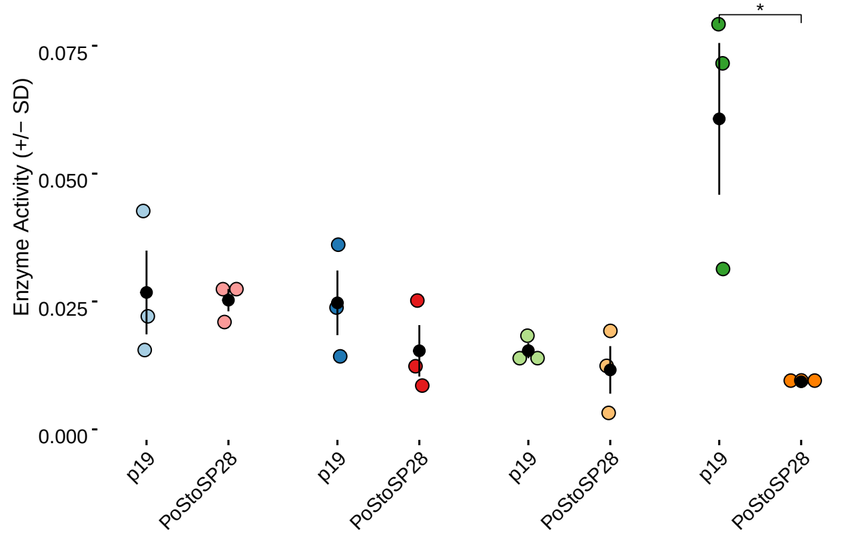

Gglucose-6-phosphate dehydrogenase

PoStoSP04 vs p19 (EXP1)

Agroinfiltrated		Systemic	
3	14	3	14
EXP1	EXP1	EXP1	EXP1

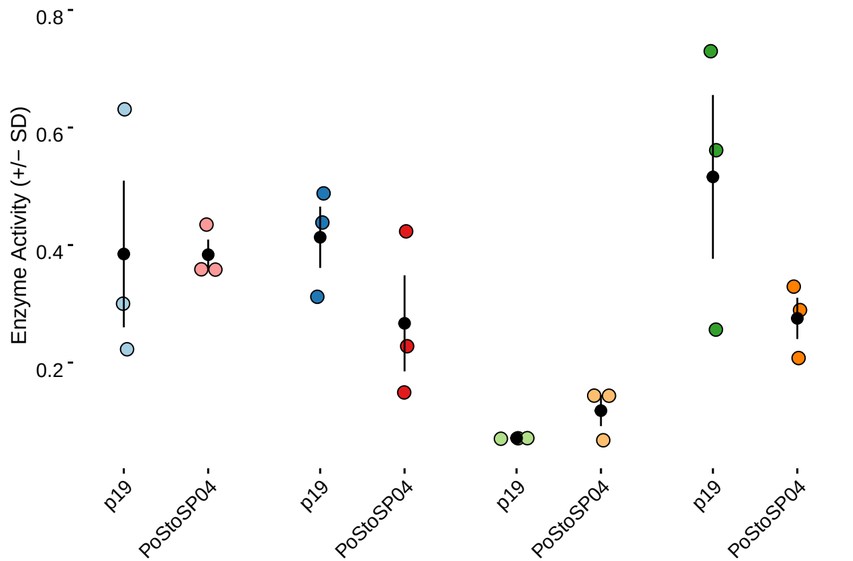

PoStoSP06 vs p19 (EXP1, EXP2)

Agroinfiltrated				Systemic			
3		14		3		14	
EXP1	EXP2	EXP1	EXP2	EXP1	EXP2	EXP1	EXP2

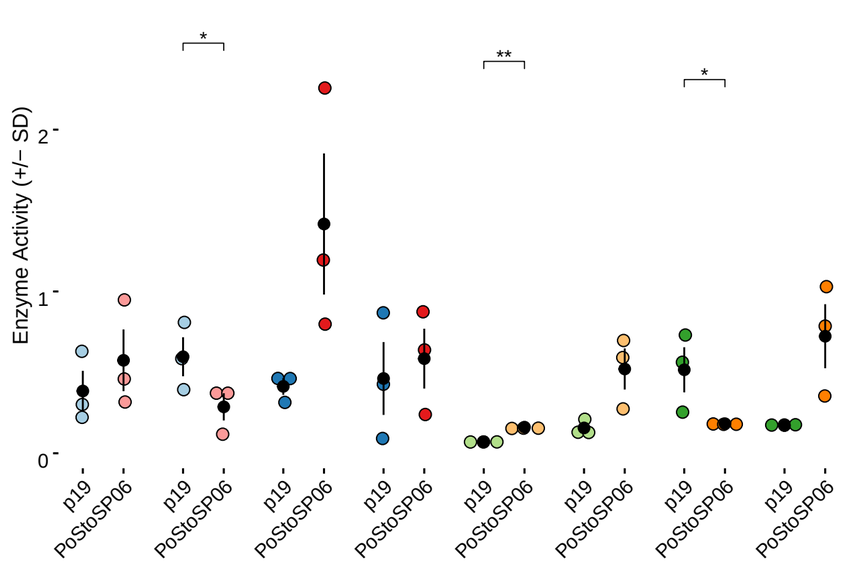

PoStoSP13 vs p19 (EXP1, EXP2)

Agroinfiltrated				Systemic			
3		14		3		14	
EXP1	EXP2	EXP1	EXP2	EXP1	EXP2	EXP1	EXP2

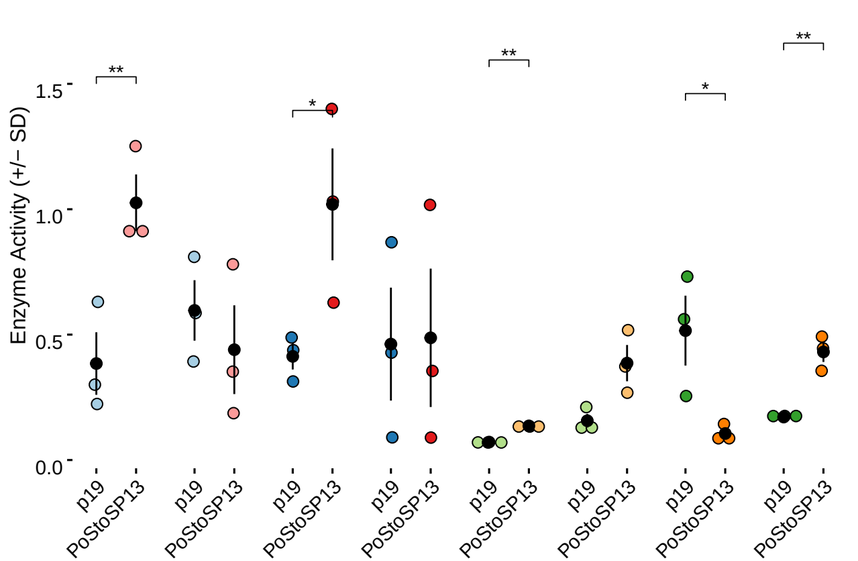

PoStoSP14 vs p19 (EXP1)

Agroinfiltrated		Systemic	
3	14	3	14
EXP1	EXP1	EXP1	EXP1

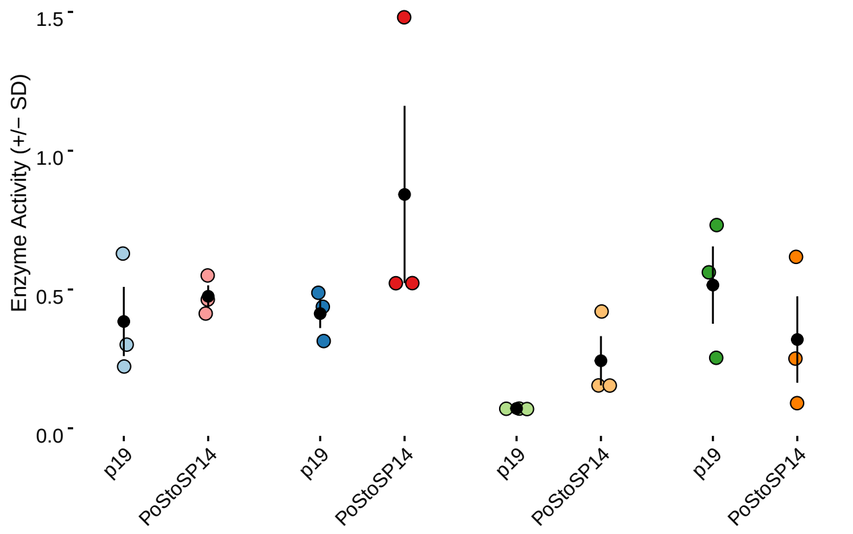

PoStoSP18 vs p19 (EXP1)

Agroinfiltrated		Systemic	
3	14	3	14
EXP1	EXP1	EXP1	EXP1

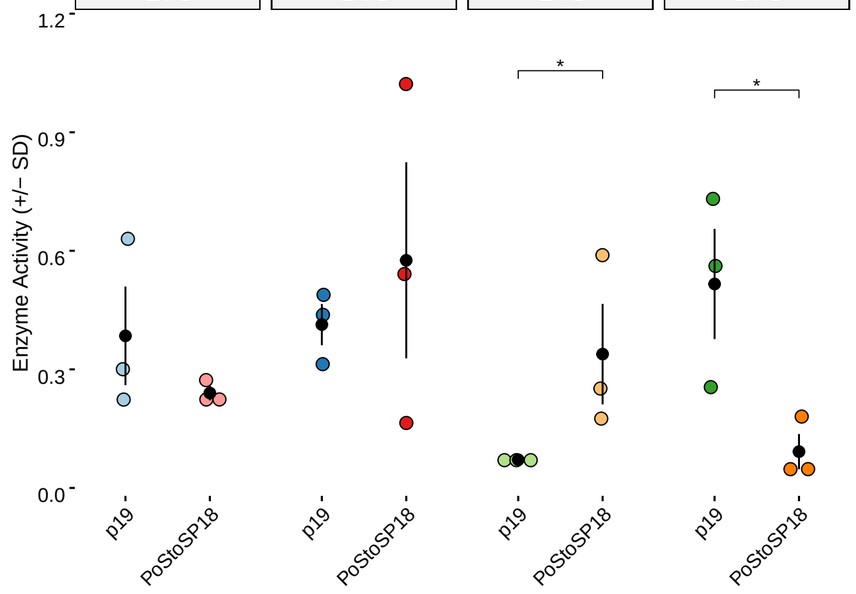

PoStoSP28 vs p19 (EXP1, EXP2)

Agroinfiltrated				Systemic			
3		14		3		14	
EXP1	EXP2	EXP1	EXP2	EXP1	EXP2	EXP1	EXP2

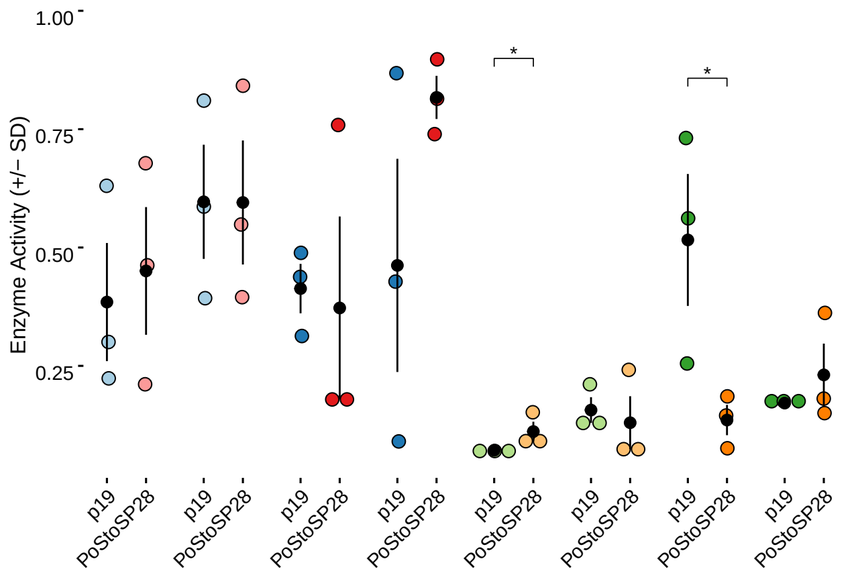

Hexokinase

PoStoSP04 vs p19 (EXP1)

Agroinfiltrated		Systemic	
3	14	3	14
EXP1	EXP1	EXP1	EXP1

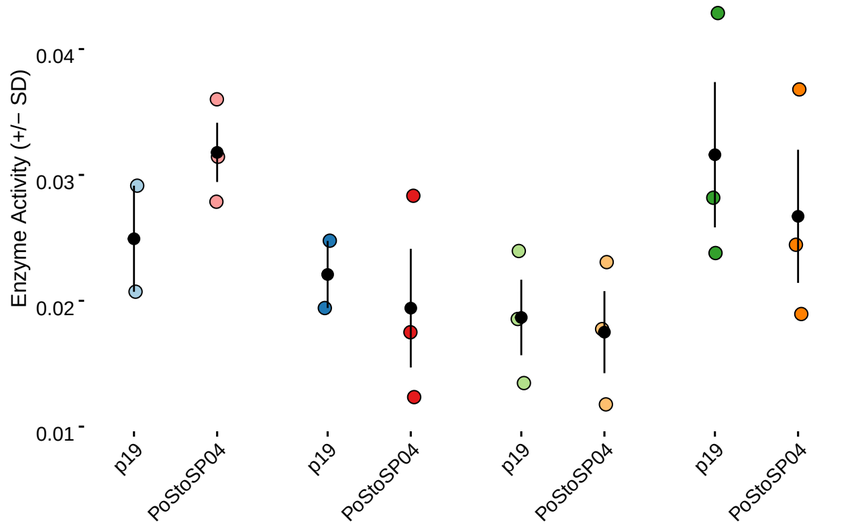

PoStoSP06 vs p19 (EXP1)

Agroinfiltrated		Systemic	
3	14	3	14
EXP1	EXP1	EXP1	EXP1

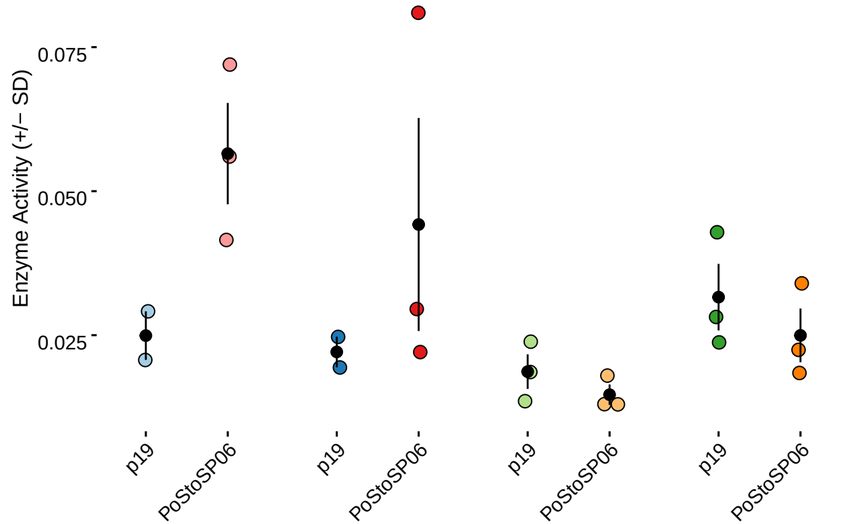

PoStoSP13 vs p19 (EXP1)

Agroinfiltrated		Systemic	
3	14	3	14
EXP1	EXP1	EXP1	EXP1

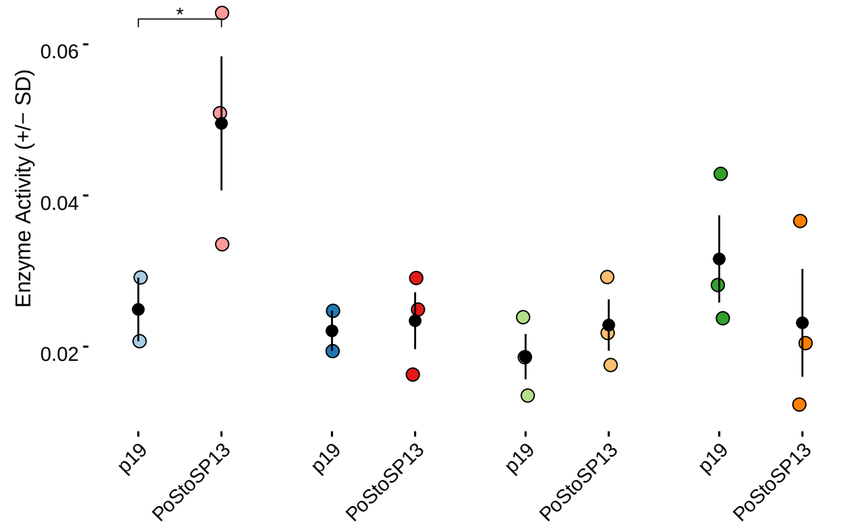

PoStoSP14 vs p19 (EXP1)

Agroinfiltrated		Systemic	
3	14	3	14
EXP1	EXP1	EXP1	EXP1

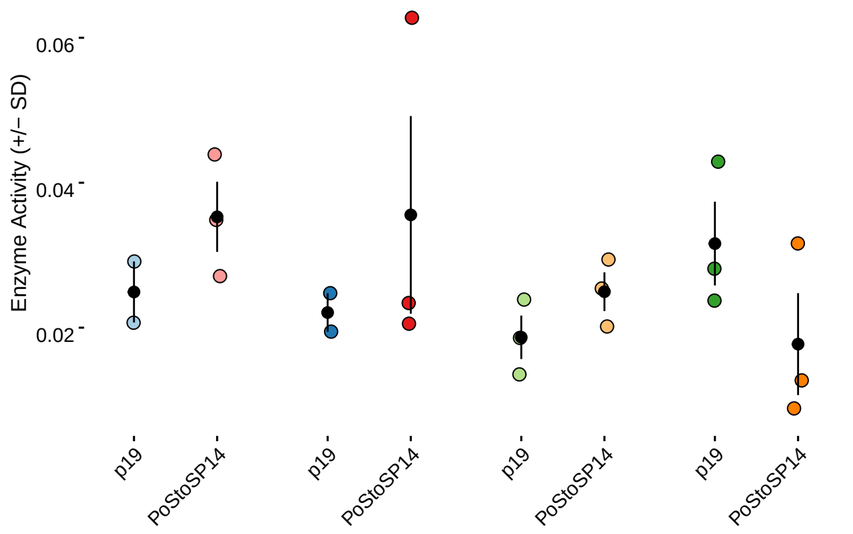

Cell wall invertase

PoStoSP04 vs p19 (EXP1)

Agroinfiltrated		Systemic	
3	14	3	14
EXP1	EXP1	EXP1	EXP1

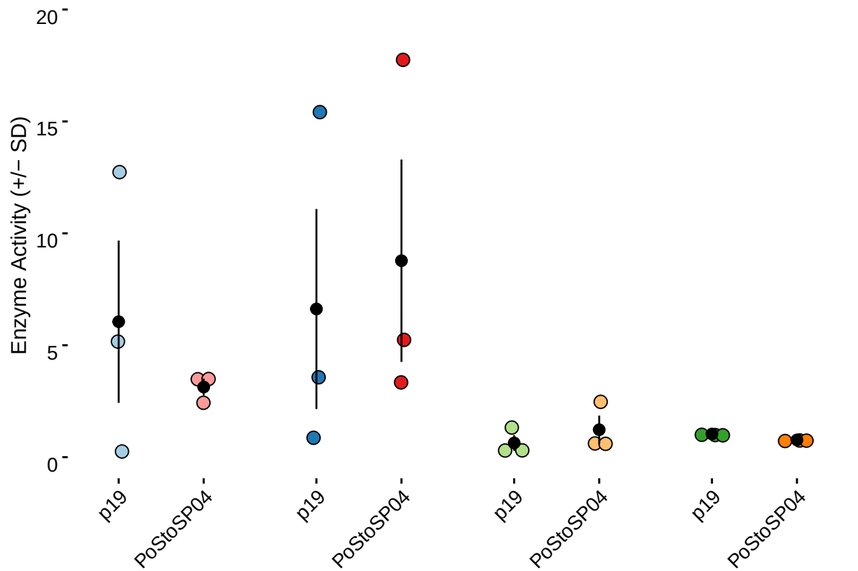

PoStoSP06 vs p19 (EXP1, EXP2)

Agroinfiltrated				Systemic			
3		14		3		14	
EXP1	EXP2	EXP1	EXP2	EXP1	EXP2	EXP1	EXP2

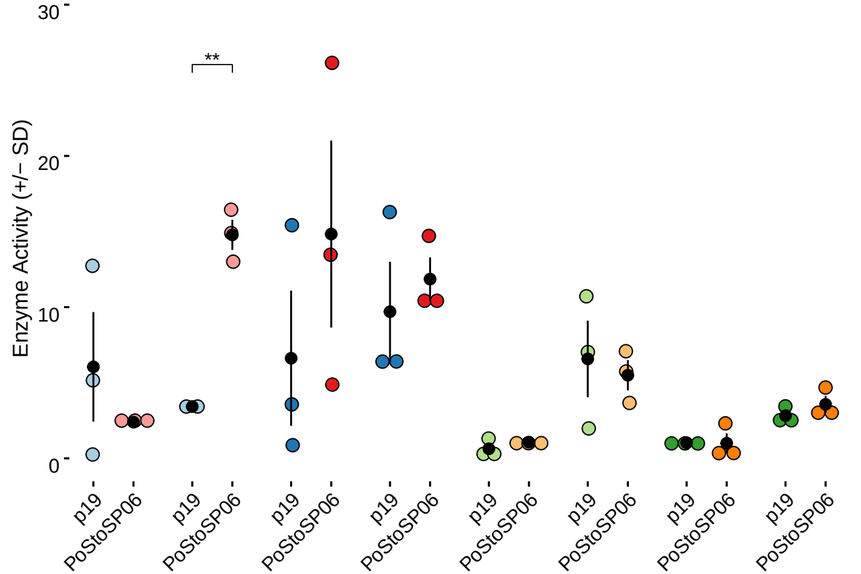

PoStoSP13 vs p19 (EXP1)

Agroinfiltrated		Systemic	
3	14	3	14
EXP1	EXP1	EXP1	EXP1

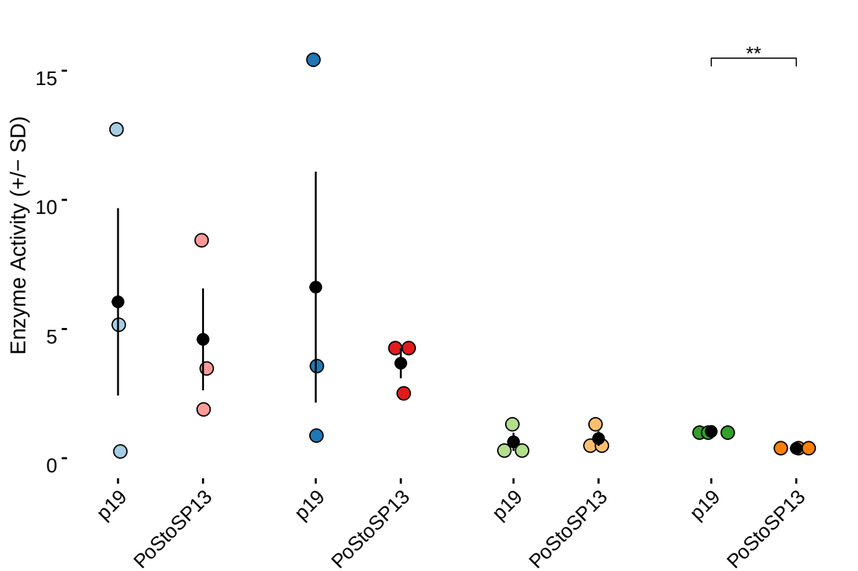

PoStoSP14 vs p19 (EXP1)

Agroinfiltrated		Systemic	
3	14	3	14
EXP1	EXP1	EXP1	EXP1

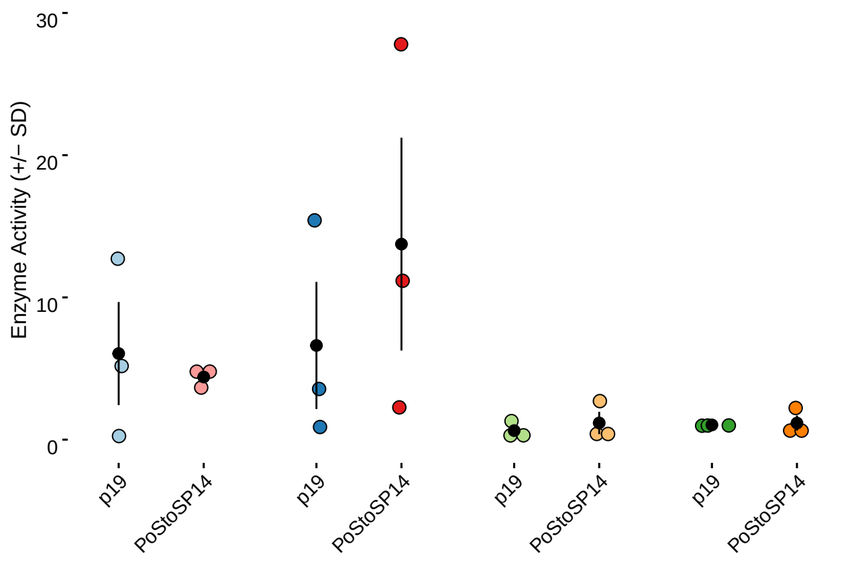

PoStoSP18 vs p19 (EXP1)

Agroinfiltrated		Systemic	
3	14	3	14
EXP1	EXP1	EXP1	EXP1

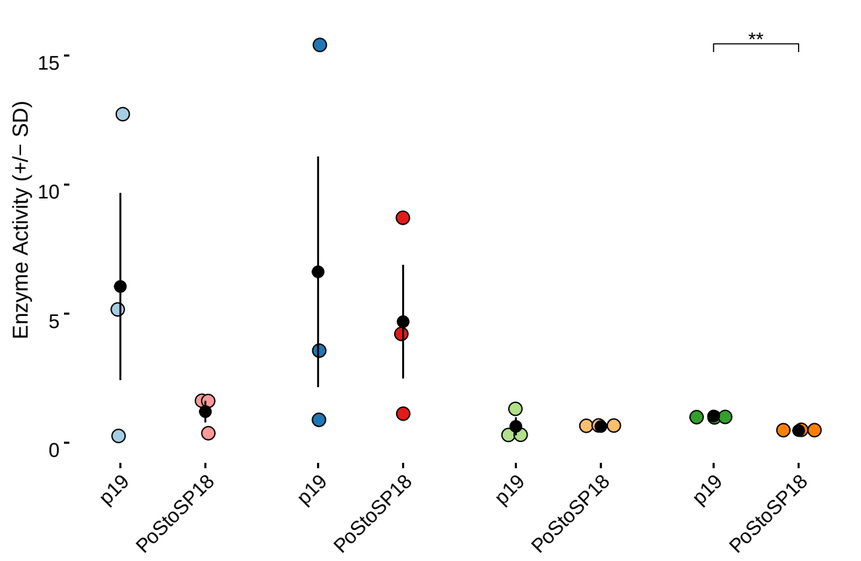

PoStoSP28 vs p19 (EXP1, EXP2)

Agroinfiltrated				Systemic			
3		14		3		14	
EXP1	EXP2	EXP1	EXP2	EXP1	EXP2	EXP1	EXP2

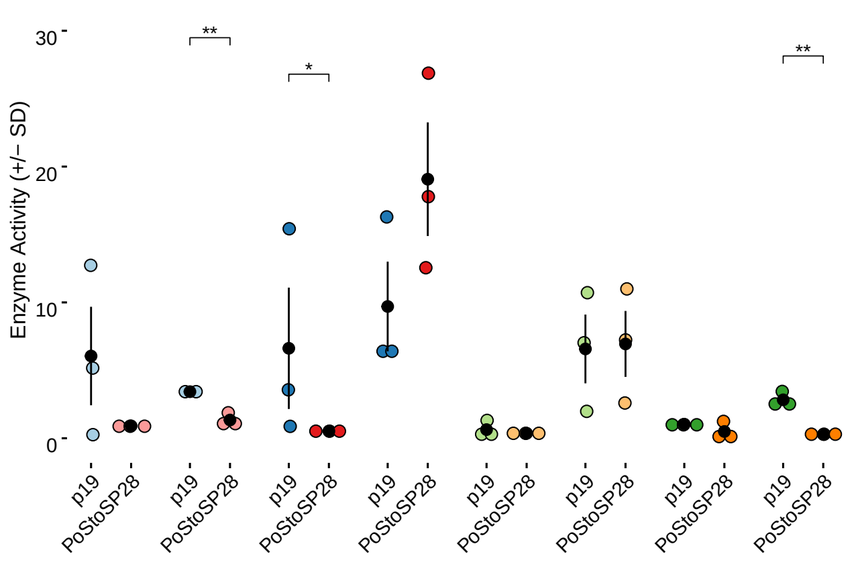

Cytoplasmic invertase

PoStoSP06 vs p19 (EXP1)

Agroinfiltrated		Systemic	
3	14	3	14
EXP1	EXP1	EXP1	EXP1

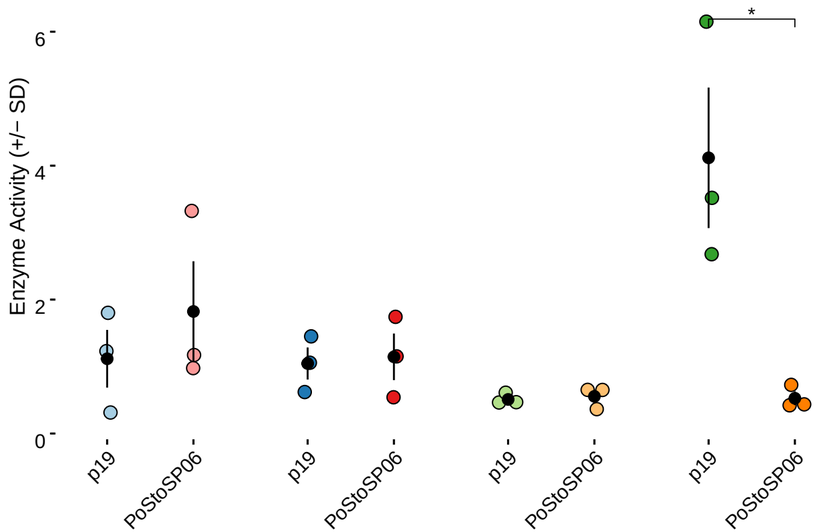

PoStoSP13 vs p19 (EXP1, EXP2)

Agroinfiltrated				Systemic			
3		14		3		14	
EXP1	EXP2	EXP1	EXP2	EXP1	EXP2	EXP1	EXP2

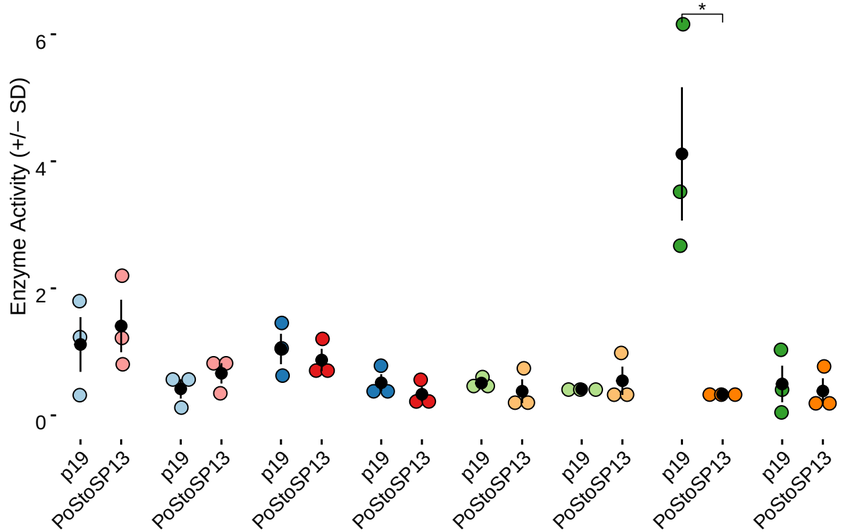

PoStoSP14 vs p19 (EXP1)

Agroinfiltrated		Systemic	
3	14	3	14
EXP1	EXP1	EXP1	EXP1

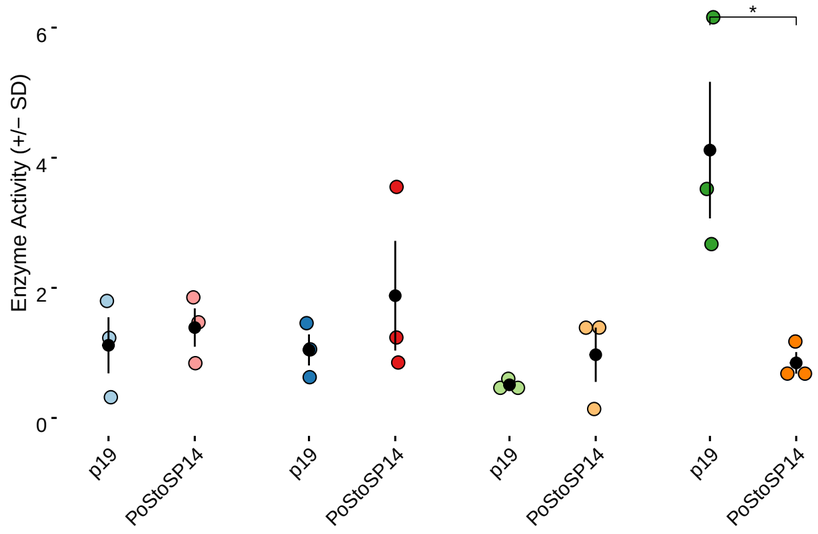

PoStoSP28 vs p19 (EXP1)

Agroinfiltrated		Systemic	
3	14	3	14
EXP1	EXP1	EXP1	EXP1

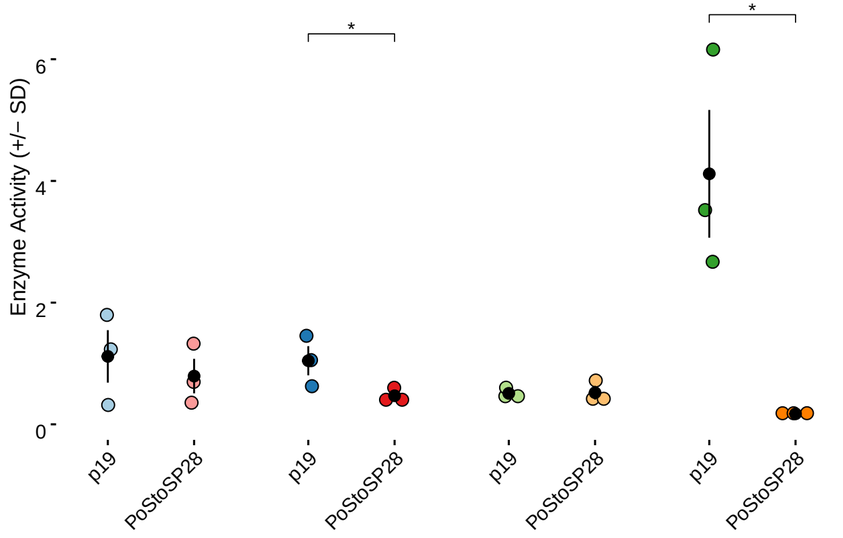

Vacuolar invertase

PoStoSP04 vs p19 (EXP1)

Agroinfiltrated		Systemic	
3	14	3	14
EXP1	EXP1	EXP1	EXP1

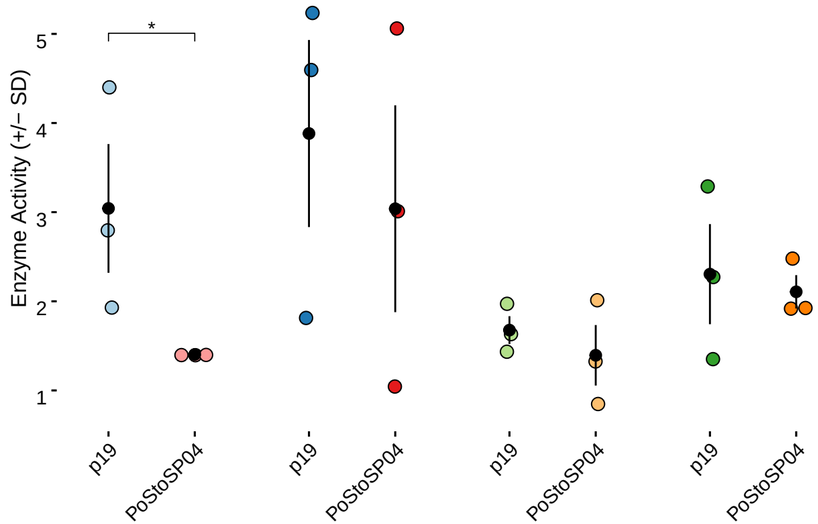

PoStoSP06 vs p19 (EXP1, EXP2)

Agroinfiltrated				Systemic			
3		14		3		14	
EXP1	EXP2	EXP1	EXP2	EXP1	EXP2	EXP1	EXP2

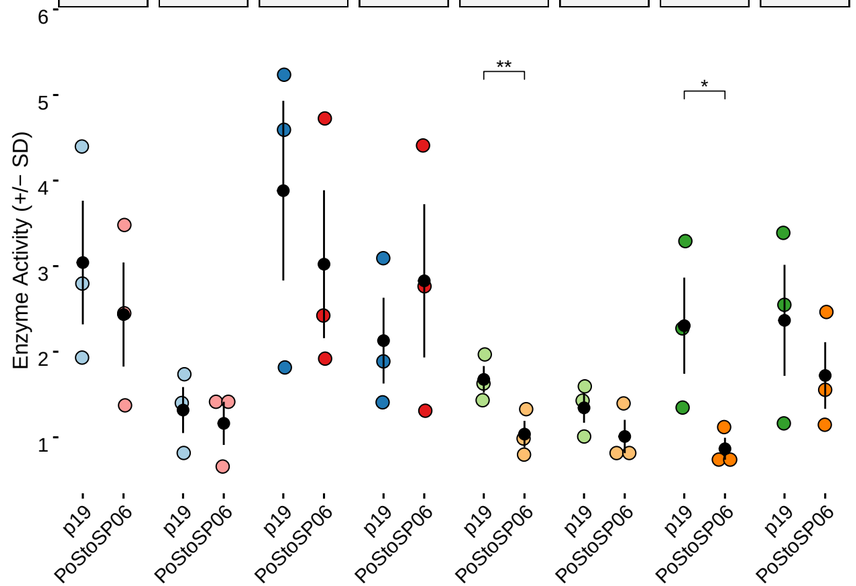

PoStoSP13 vs p19 (EXP1, EXP2)

Agroinfiltrated				Systemic			
3		14		3		14	
EXP1	EXP2	EXP1	EXP2	EXP1	EXP2	EXP1	EXP2

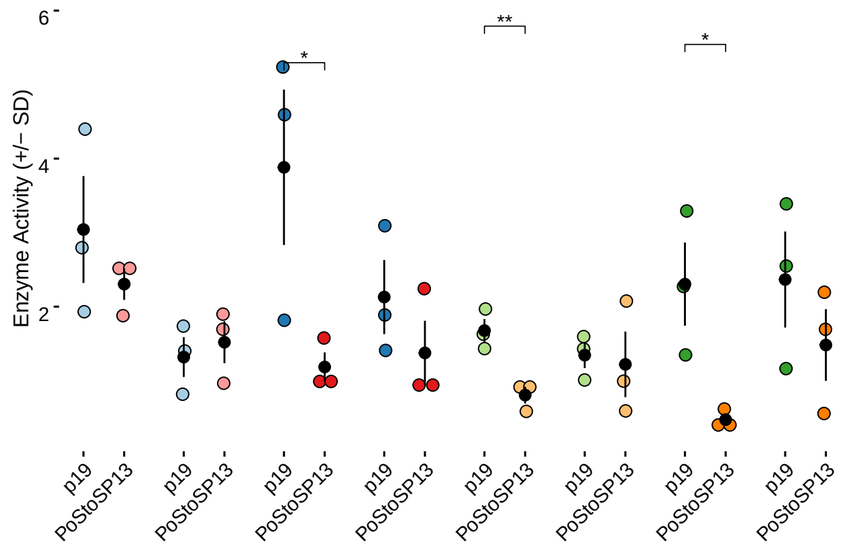

PoStoSP14 vs p19 (EXP1)

Agroinfiltrated		Systemic	
3	14	3	14
EXP1	EXP1	EXP1	EXP1

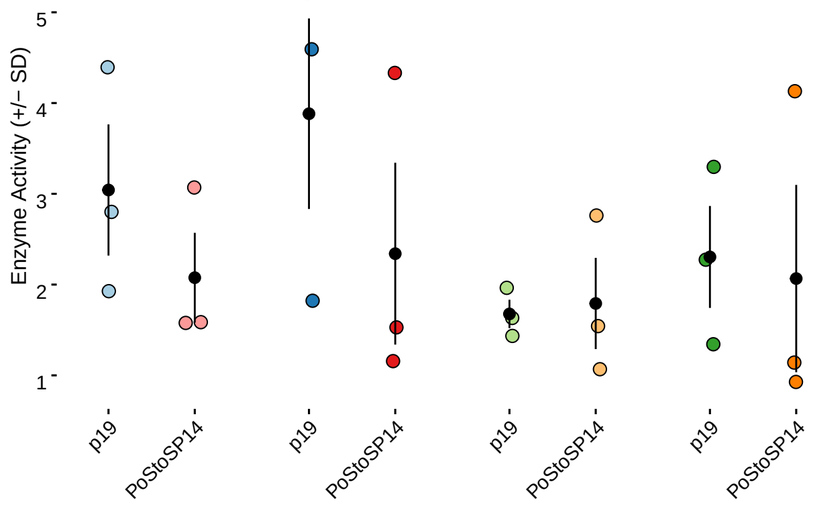

PoStoSP18 vs p19 (EXP1)

Agroinfiltrated		Systemic	
3	14	3	14
EXP1	EXP1	EXP1	EXP1

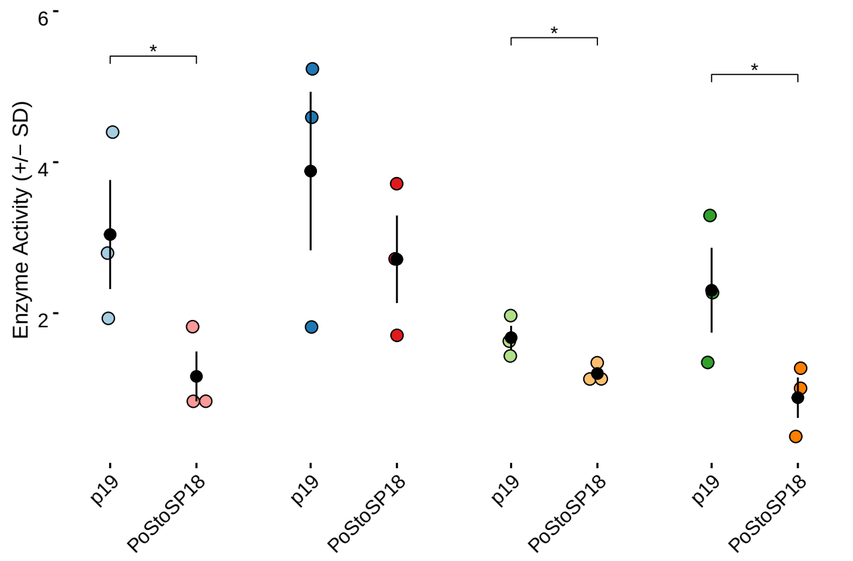

PoStoSP28 vs p19 (EXP1)

Agroinfiltrated		Systemic	
3	14	3	14
EXP1	EXP1	EXP1	EXP1

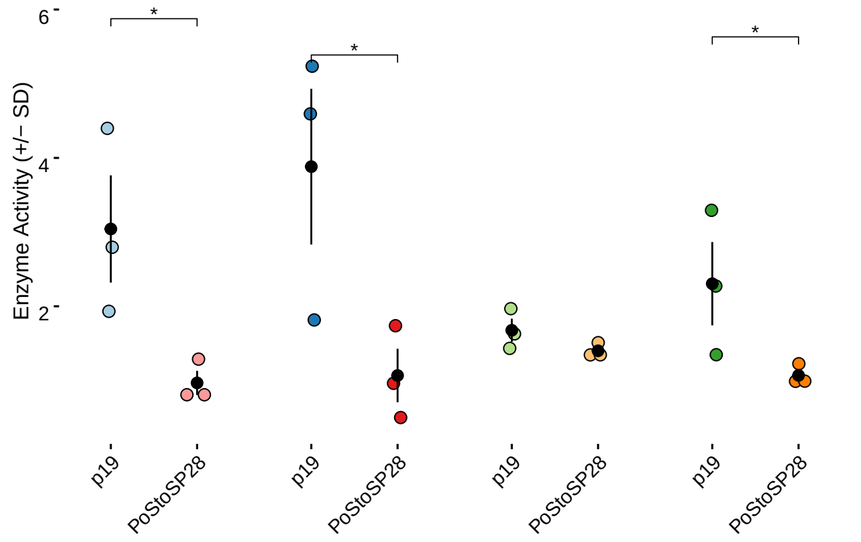

Phosphofructokinase

PoStoSP04 vs p19 (EXP1)

Agroinfiltrated		Systemic	
3	14	3	14
EXP1	EXP1	EXP1	EXP1

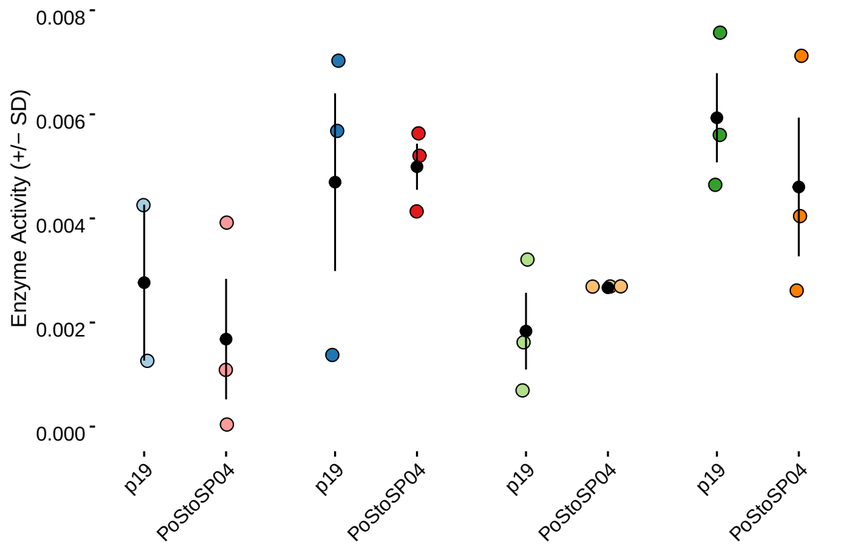

PoStoSP06 vs p19 (EXP1)

Agroinfiltrated		Systemic	
3	14	3	14
EXP1	EXP1	EXP1	EXP1

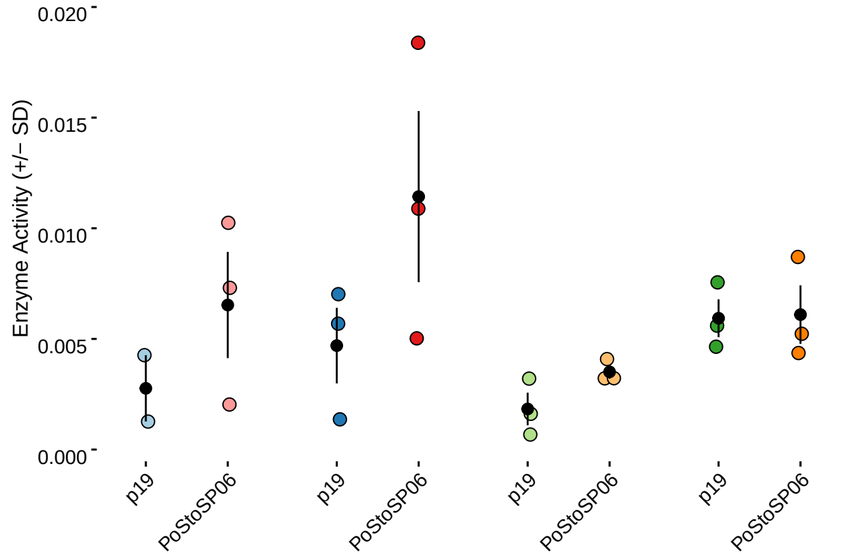

PoStoSP13 vs p19 (EXP1)

Agroinfiltrated		Systemic	
3	14	3	14
EXP1	EXP1	EXP1	EXP1

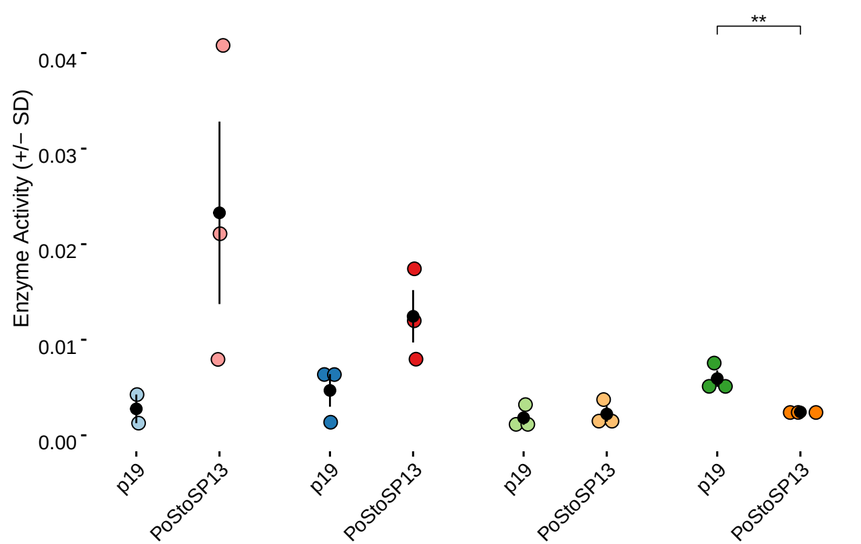

PoStoSP14 vs p19 (EXP1)

Agroinfiltrated		Systemic	
3	14	3	14
EXP1	EXP1	EXP1	EXP1

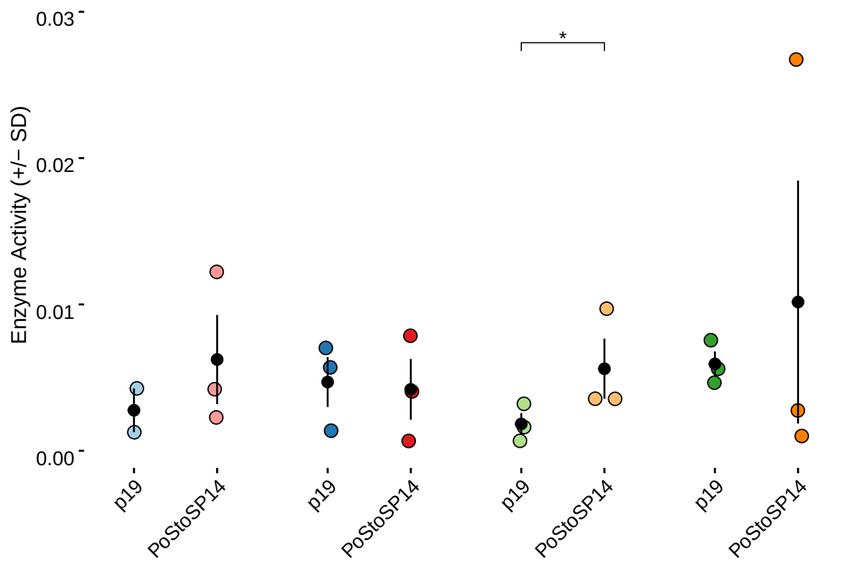

PoStoSP28 vs p19 (EXP1)

Agroinfiltrated		Systemic	
3	14	3	14
EXP1	EXP1	EXP1	EXP1

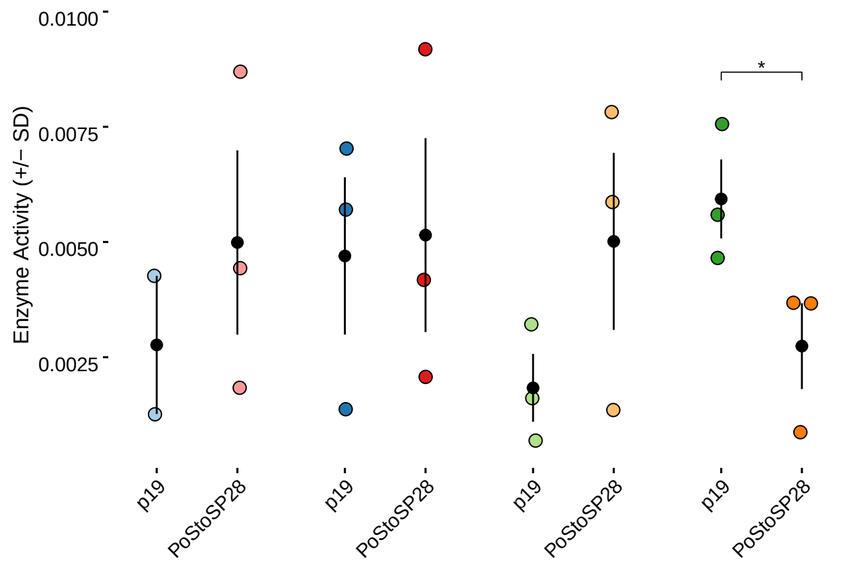

Phosphoglucoisomerase

PoStoSP04 vs p19 (EXP1)

Agroinfiltrated		Systemic	
3	14	3	14
EXP1	EXP1	EXP1	EXP1

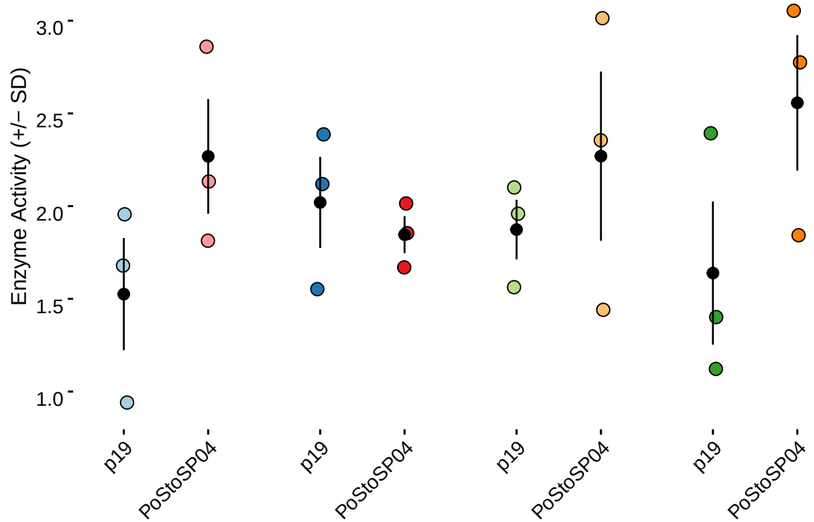

PoStoSP06 vs p19 (EXP1, EXP2)

Agroinfiltrated				Systemic			
3		14		3		14	
EXP1	EXP2	EXP1	EXP2	EXP1	EXP2	EXP1	EXP2

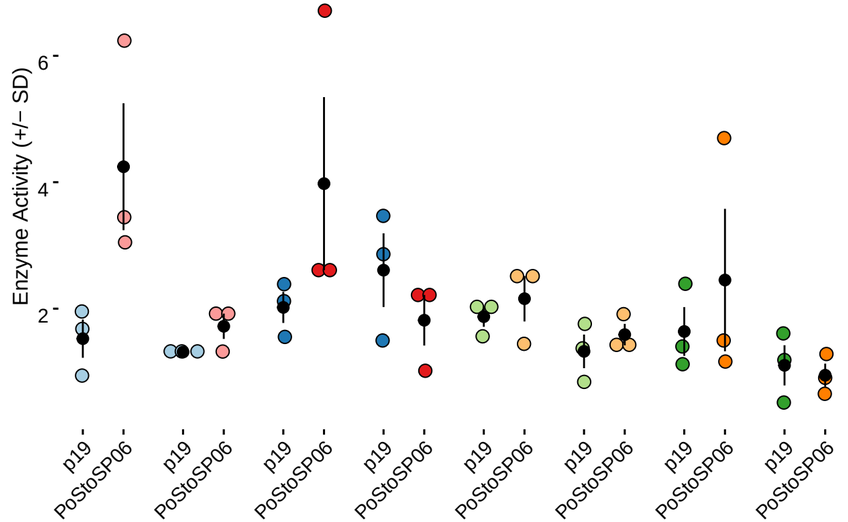

PoStoSP13 vs p19 (EXP2)

Agroinfiltrated		Systemic	
3	14	3	14
EXP2	EXP2	EXP2	EXP2

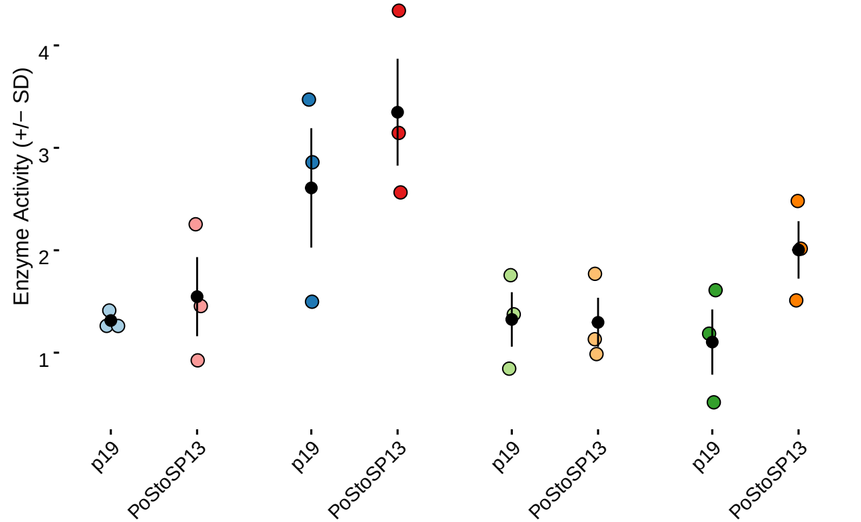

PoStoSP14 vs p19 (EXP1)

Agroinfiltrated		Systemic	
3	14	3	14
EXP1	EXP1	EXP1	EXP1

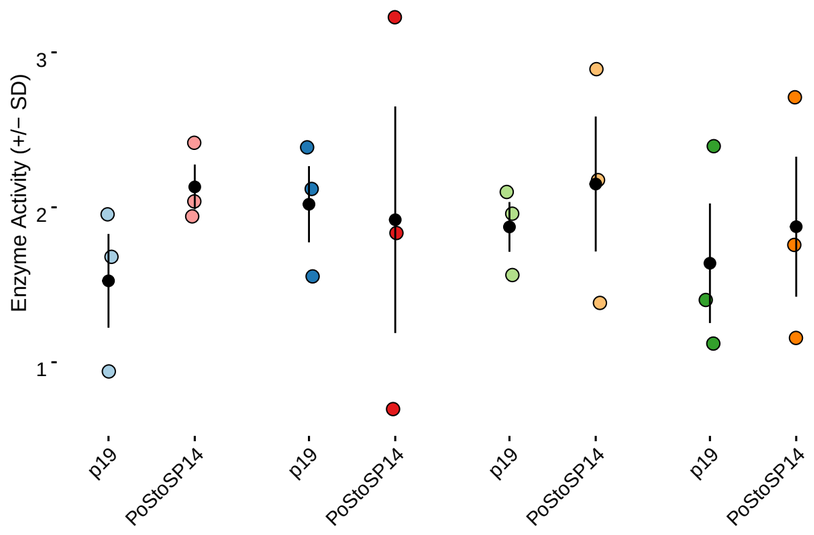

PoStoSP18 vs p19 (EXP1)

Agroinfiltrated		Systemic	
3	14	3	14
EXP1	EXP1	EXP1	EXP1

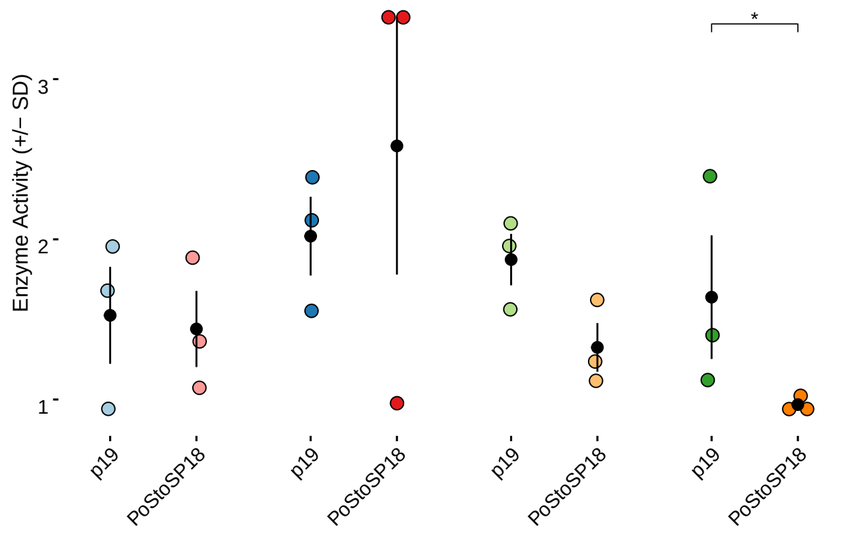

PoStoSP28 vs p19 (EXP1, EXP2)

Agroinfiltrated				Systemic			
3		14		3		14	
EXP1	EXP2	EXP1	EXP2	EXP1	EXP2	EXP1	EXP2

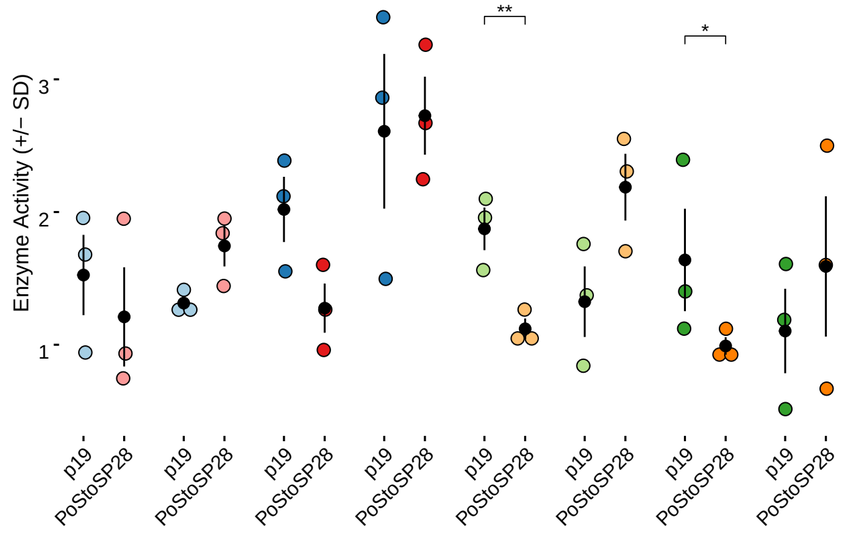

Phosphoglucomutase

PoStoSP04 vs p19 (EXP1)

Agroinfiltrated		Systemic	
3	14	3	14
EXP1	EXP1	EXP1	EXP1

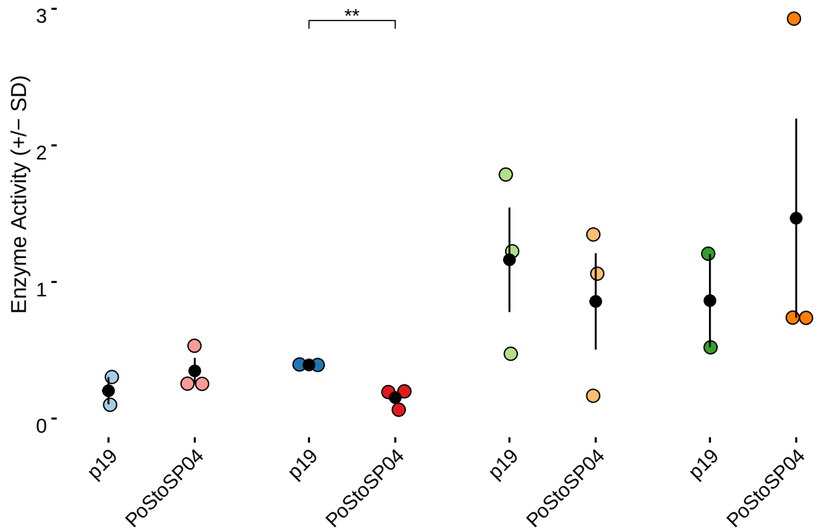

PoStoSP06 vs p19 (EXP1, EXP2)

Agroinfiltrated				Systemic			
3		14		3		14	
EXP1	EXP2	EXP1	EXP2	EXP1	EXP2	EXP1	EXP2

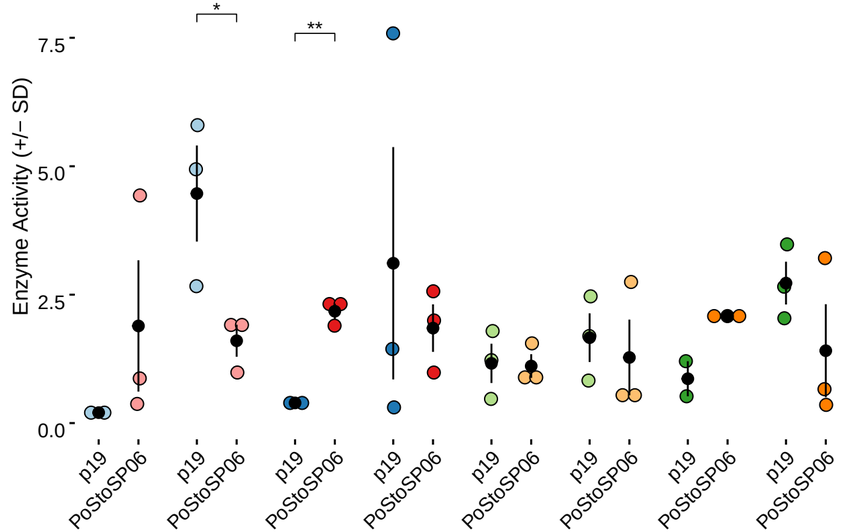

PoStoSP13 vs p19 (EXP1, EXP2)

Agroinfiltrated				Systemic			
3		14		3		14	
EXP1	EXP2	EXP1	EXP2	EXP1	EXP2	EXP1	EXP2

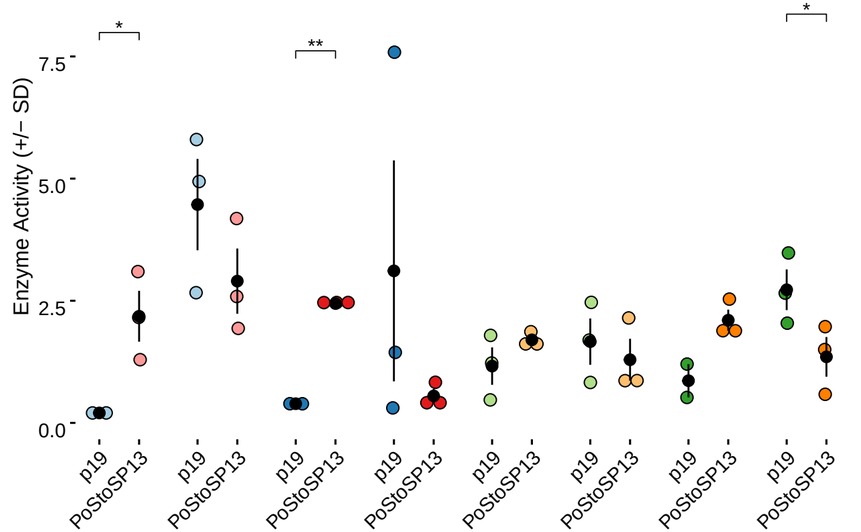

PoStoSP14 vs p19 (EXP1)

Agroinfiltrated		Systemic	
3	14	3	14
EXP1	EXP1	EXP1	EXP1

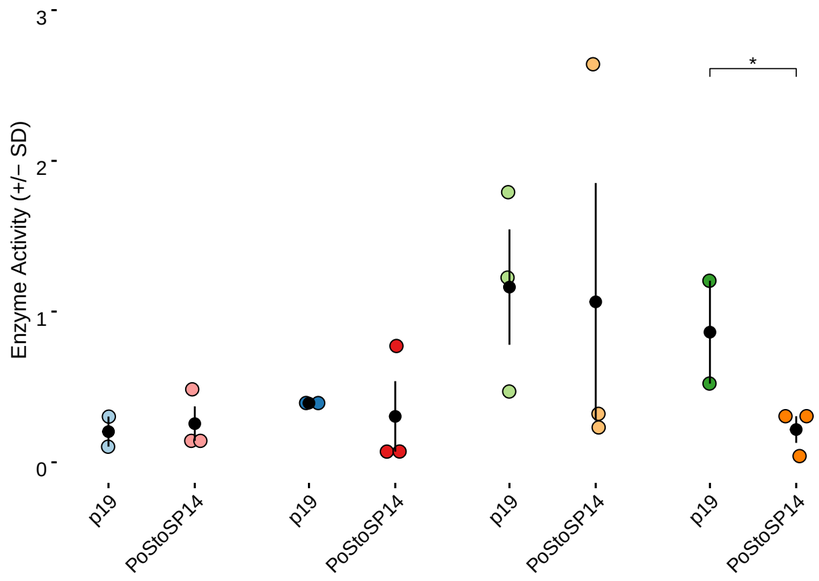

PoStoSP18 vs p19 (EXP1)

Agroinfiltrated		Systemic	
3	14	3	14
EXP1	EXP1	EXP1	EXP1

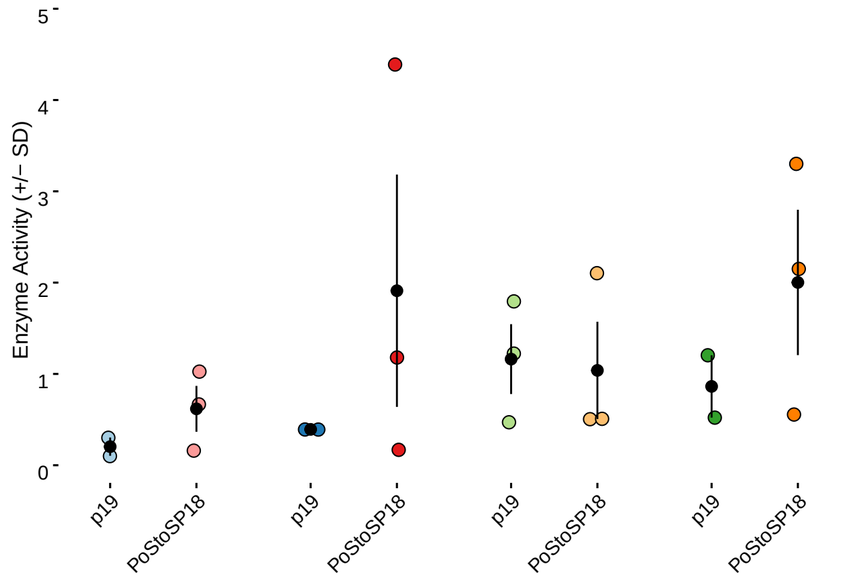

PoStoSP28 vs p19 (EXP1, EXP2)

Agroinfiltrated				Systemic			
3		14		3		14	
EXP1	EXP2	EXP1	EXP2	EXP1	EXP2	EXP1	EXP2

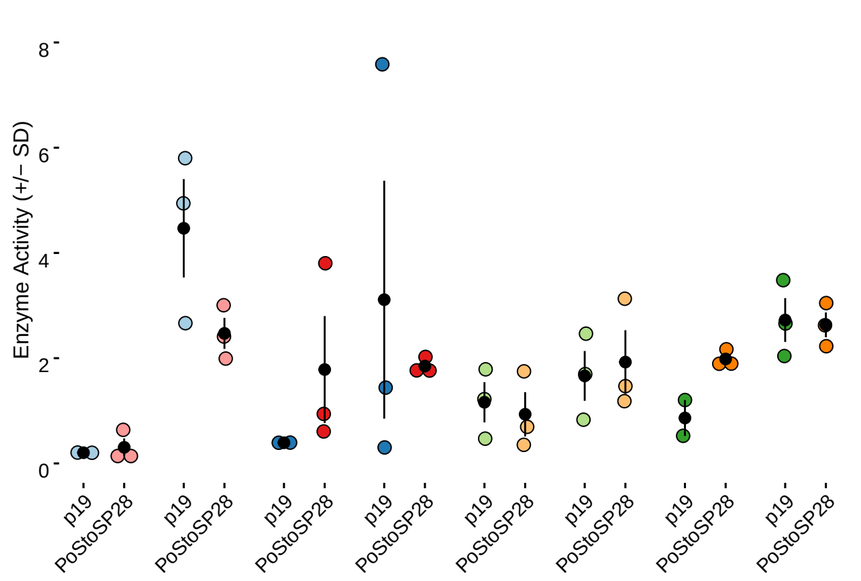

UDP-glucose pyrophosphorylase

PoStoSP04 vs p19 (EXP1)

Agroinfiltrated		Systemic	
3	14	3	14
EXP1	EXP1	EXP1	EXP1

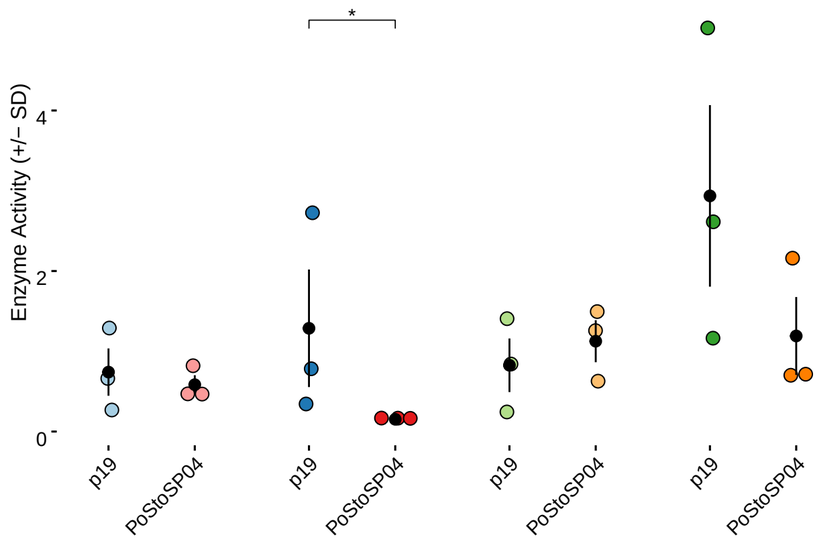

PoStoSP06 vs p19 (EXP2)

Agroinfiltrated		Systemic	
3	14	3	14
EXP2	EXP2	EXP2	EXP2

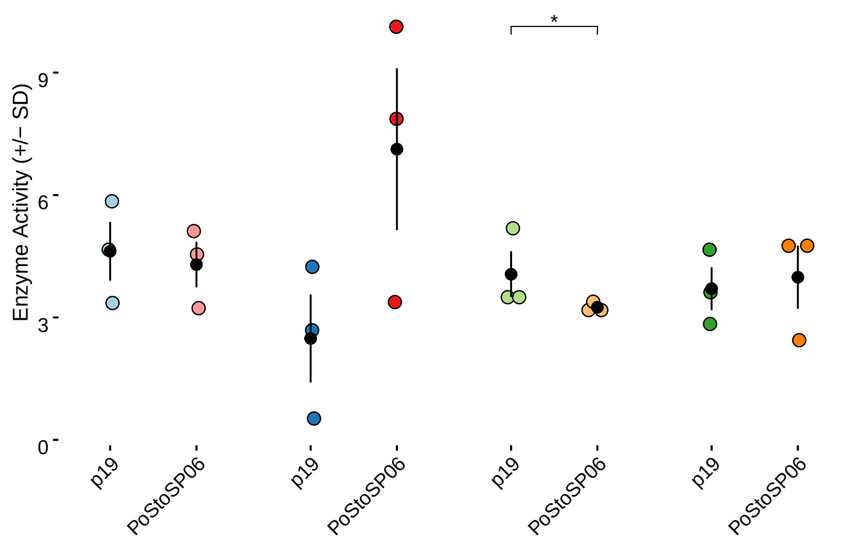

PoStoSP13 vs p19 (EXP2)

Agroinfiltrated		Systemic	
3	14	3	14
EXP2	EXP2	EXP2	EXP2

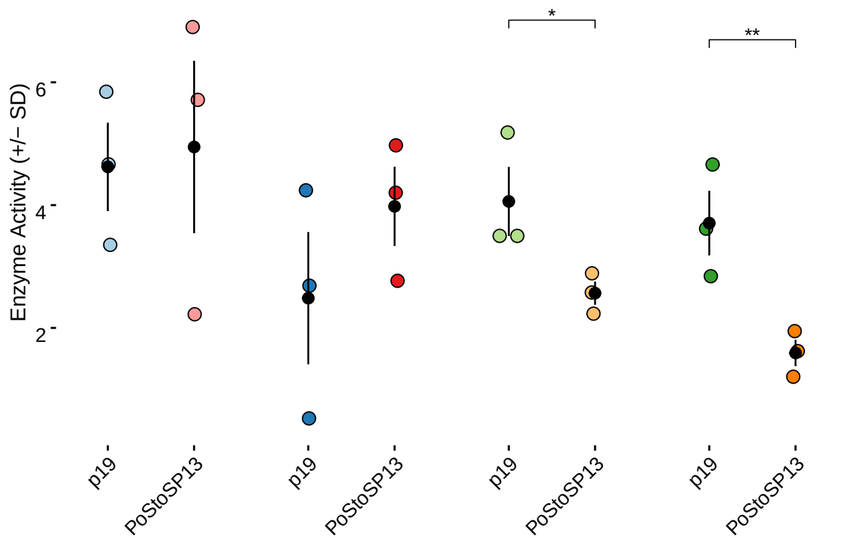

PoStoSP18 vs p19 (EXP1)

Agroinfiltrated		Systemic	
3	14	3	14
EXP1	EXP1	EXP1	EXP1

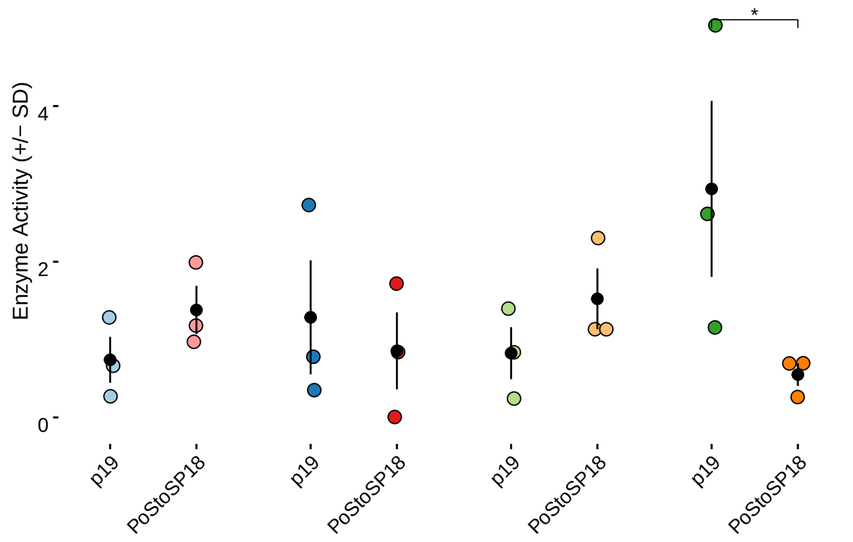

PoStoSP28 vs p19 (EXP1, EXP2)

Agroinfiltrated				Systemic			
3		14		3		14	
EXP1	EXP2	EXP1	EXP2	EXP1	EXP2	EXP1	EXP2

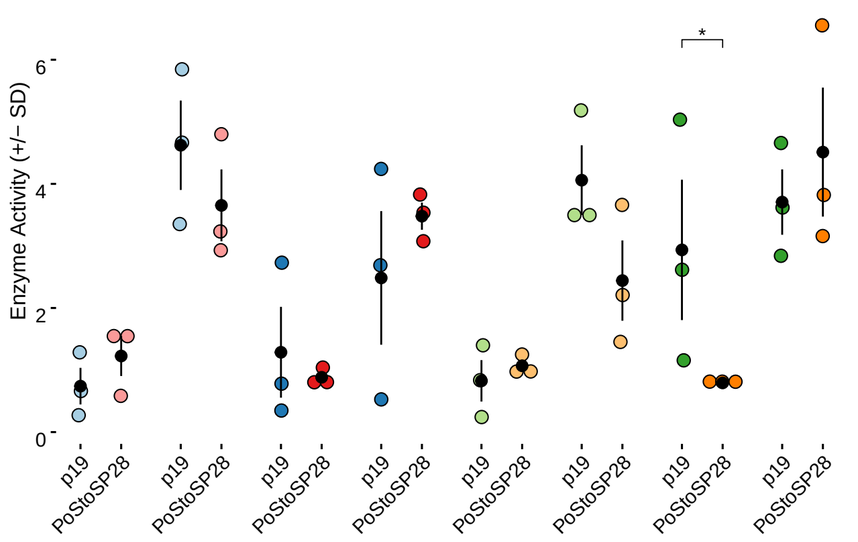

Apoplastic peroxidases

PoStoSP04 vs p19 (EXP1)

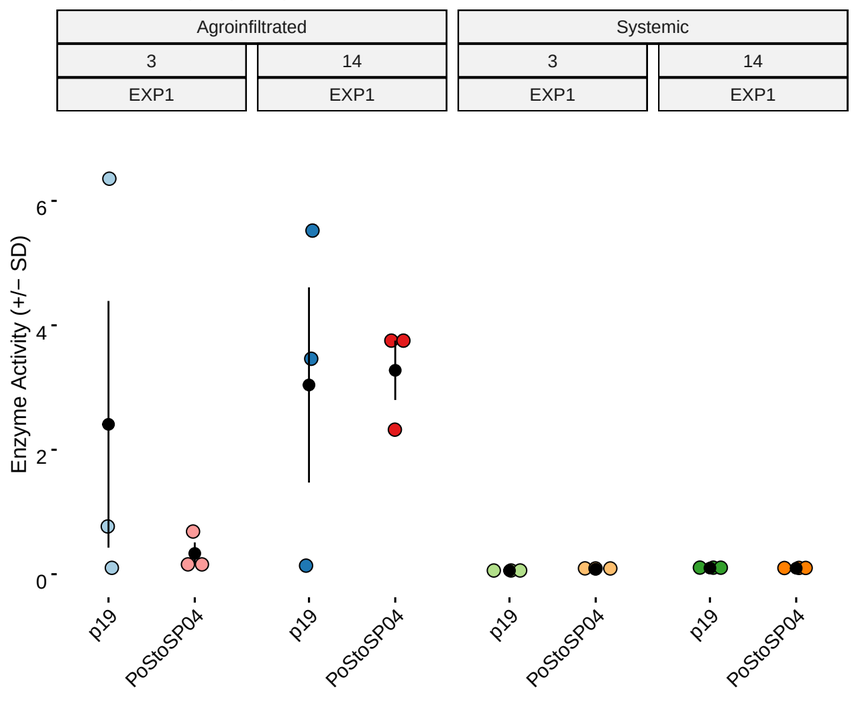

PoStoSP06 vs p19 (EXP1, EXP2)

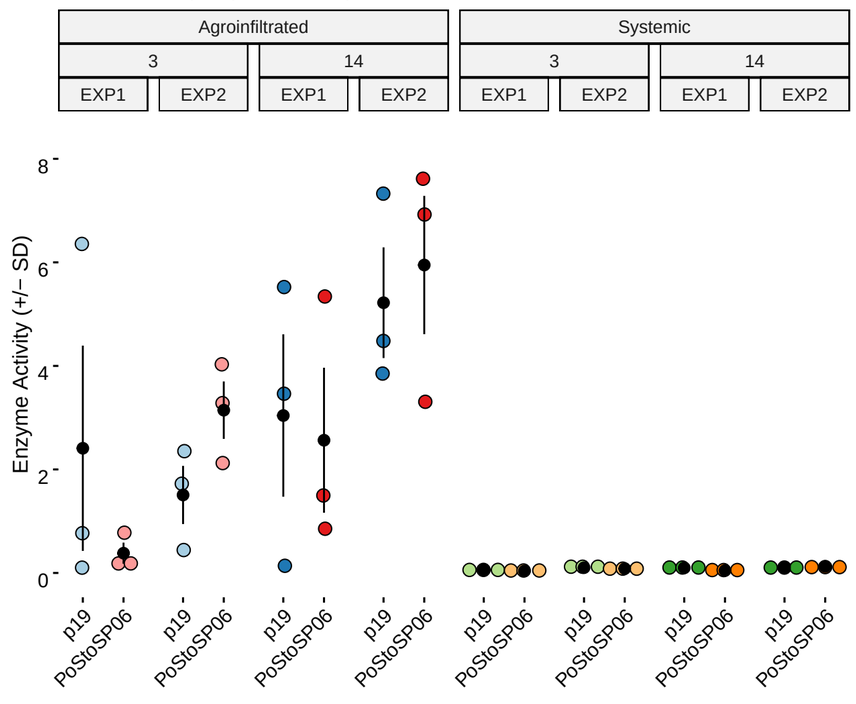

PoStoSP13 vs p19 (EXP1, EXP2)

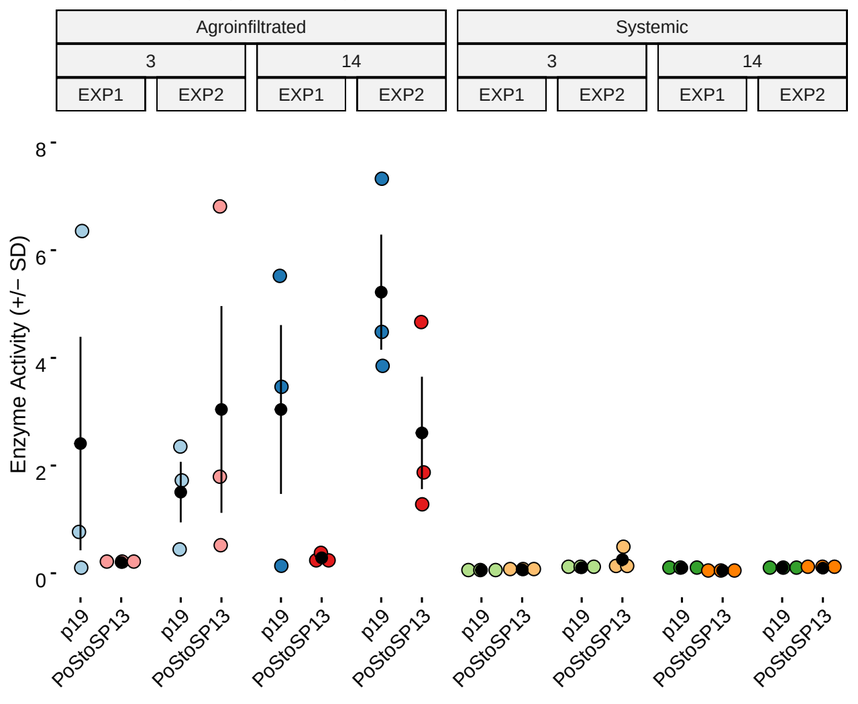

PoStoSP14 vs p19 (EXP1)

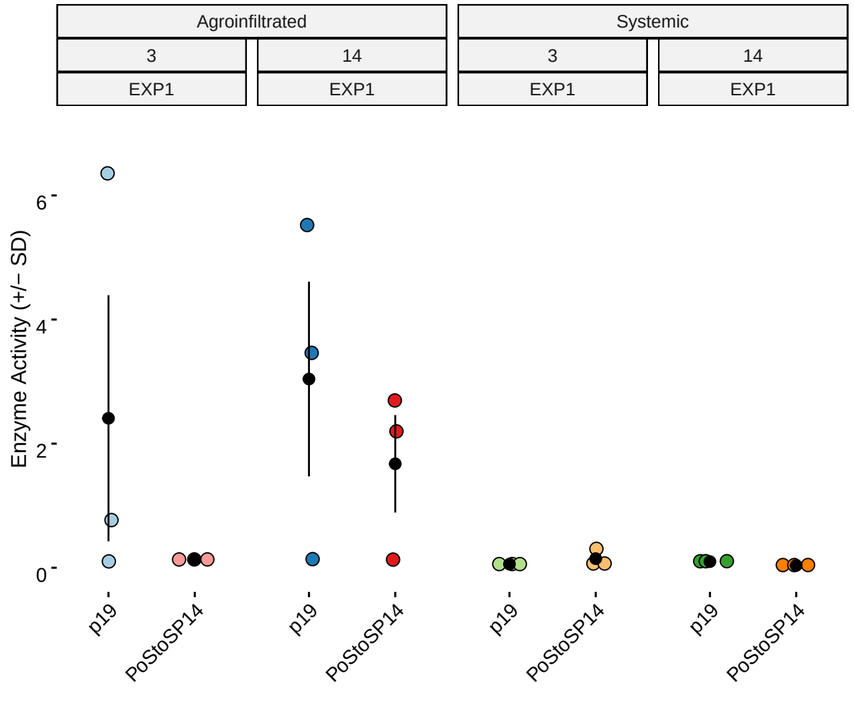

PoStoSP18 vs p19 (EXP1)

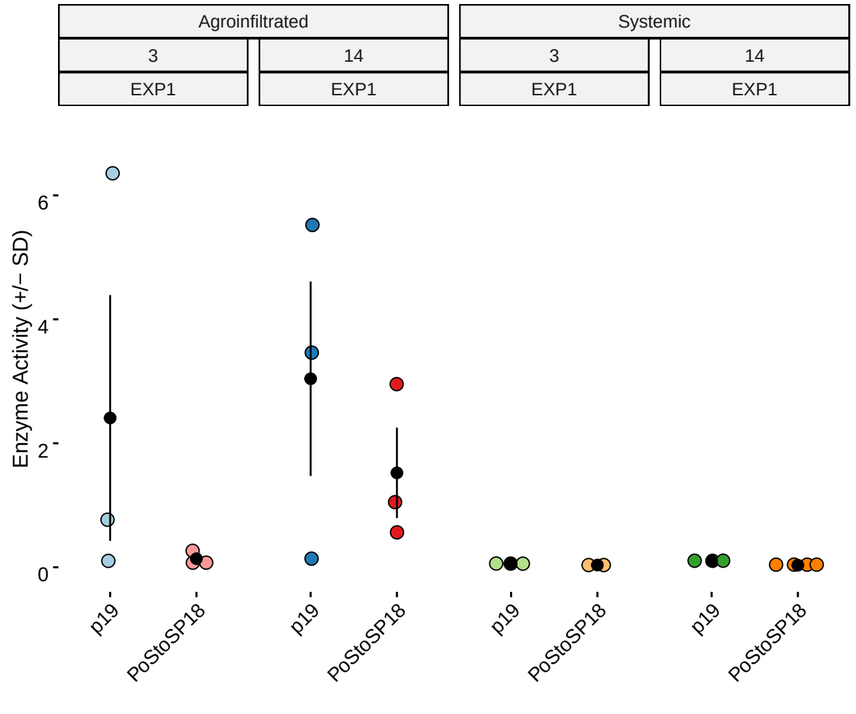

PoStoSP28 vs p19 (EXP1, EXP2)

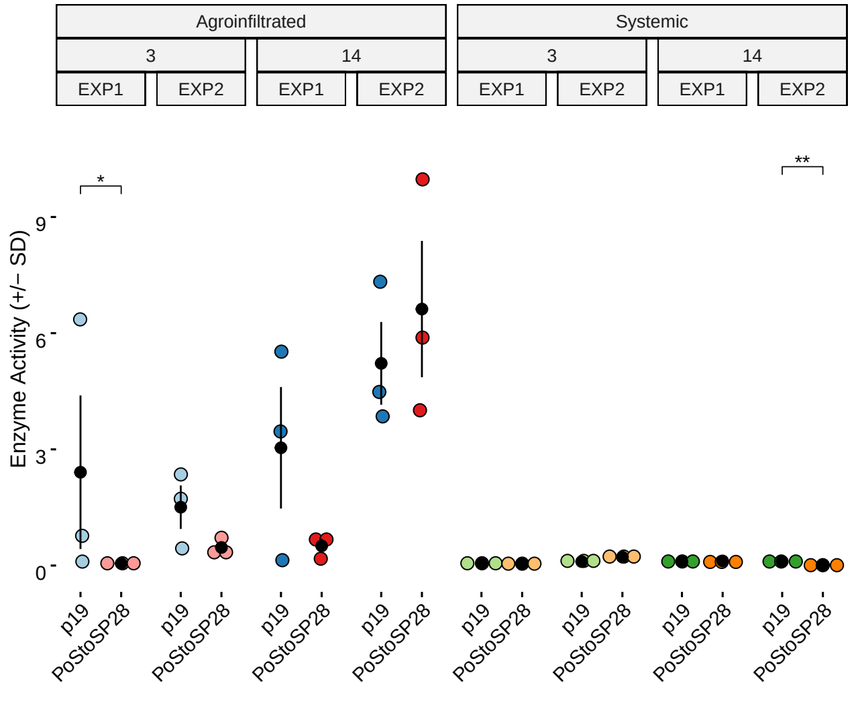

Ascorbate peroxidase

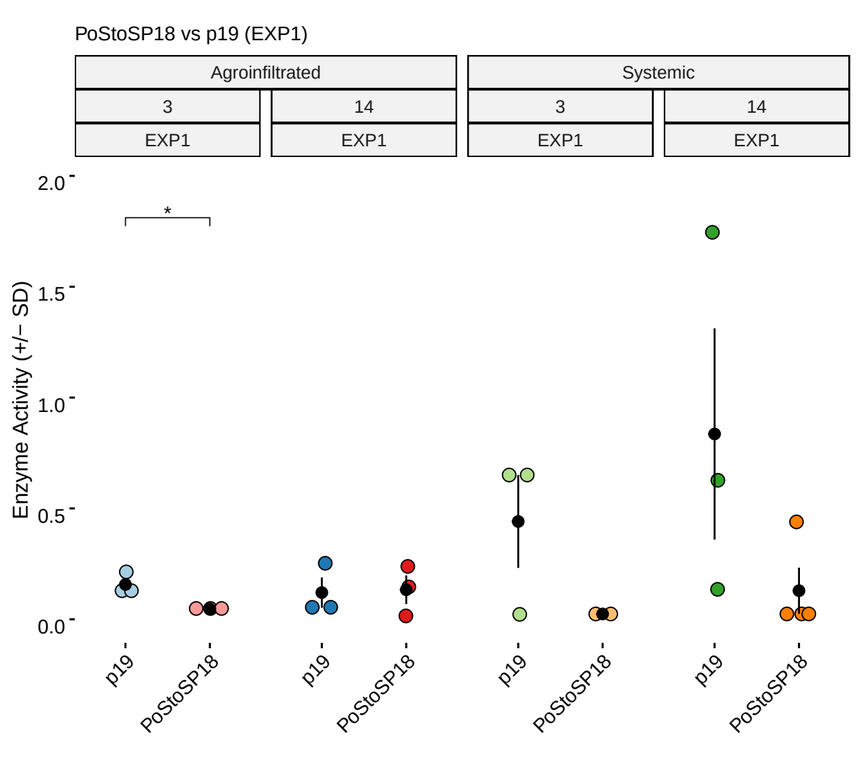

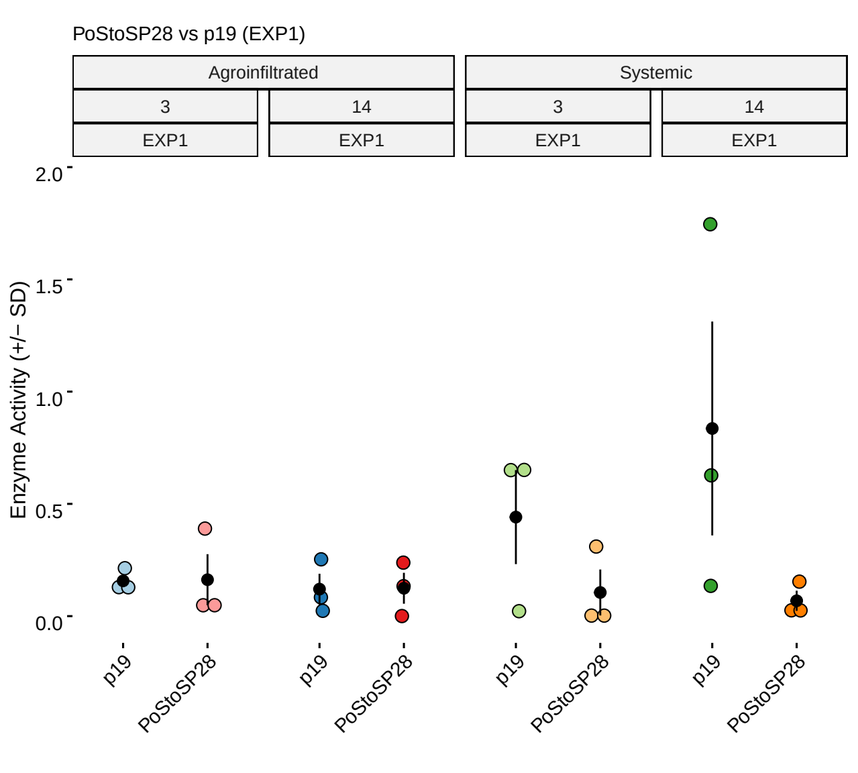

Catalase

PoStoSP04 vs p19 (EXP1)

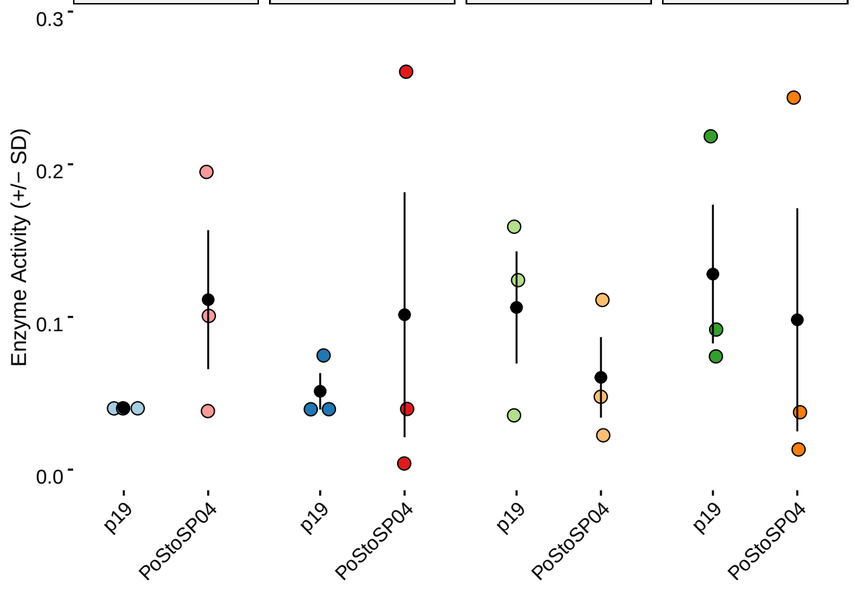

PoStoSP06 vs p19 (EXP1, EXP2)

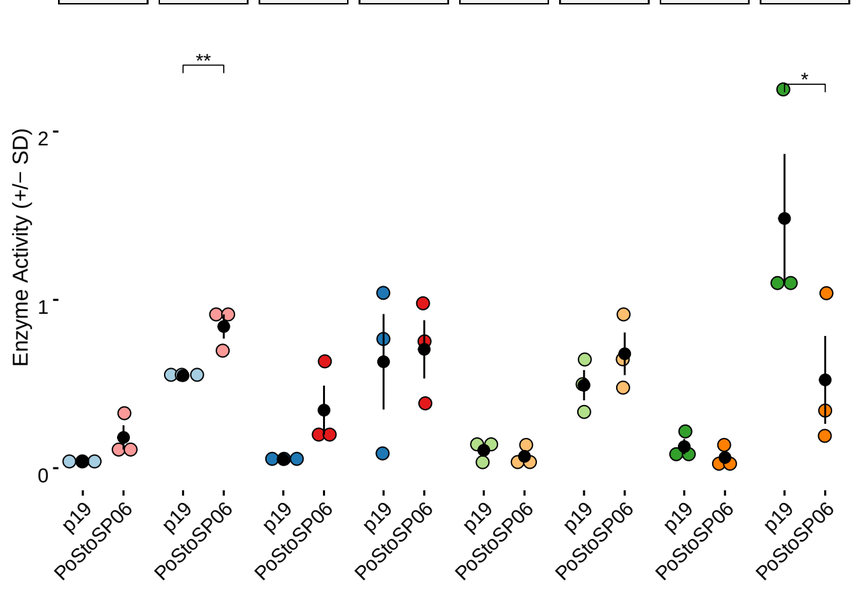

PoStoSP13 vs p19 (EXP1, EXP2)

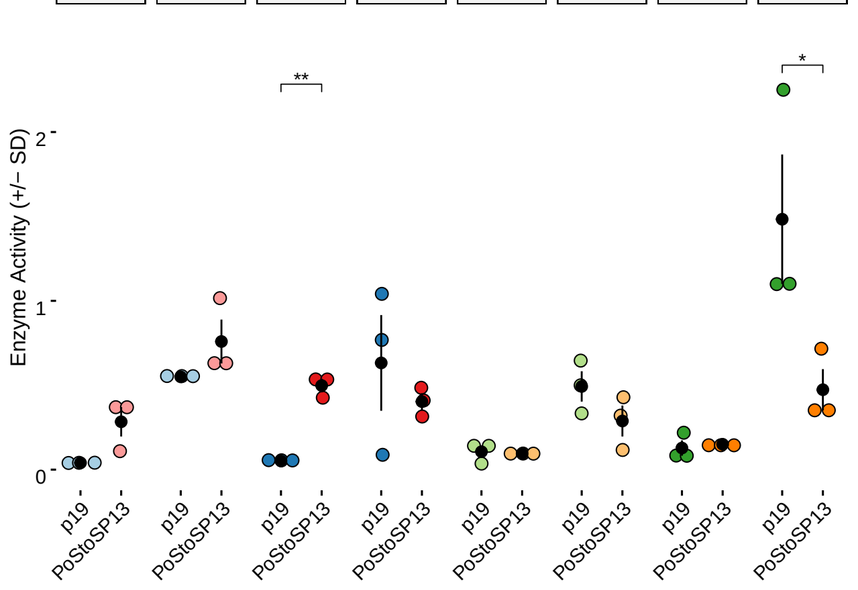

PoStoSP14 vs p19 (EXP1)

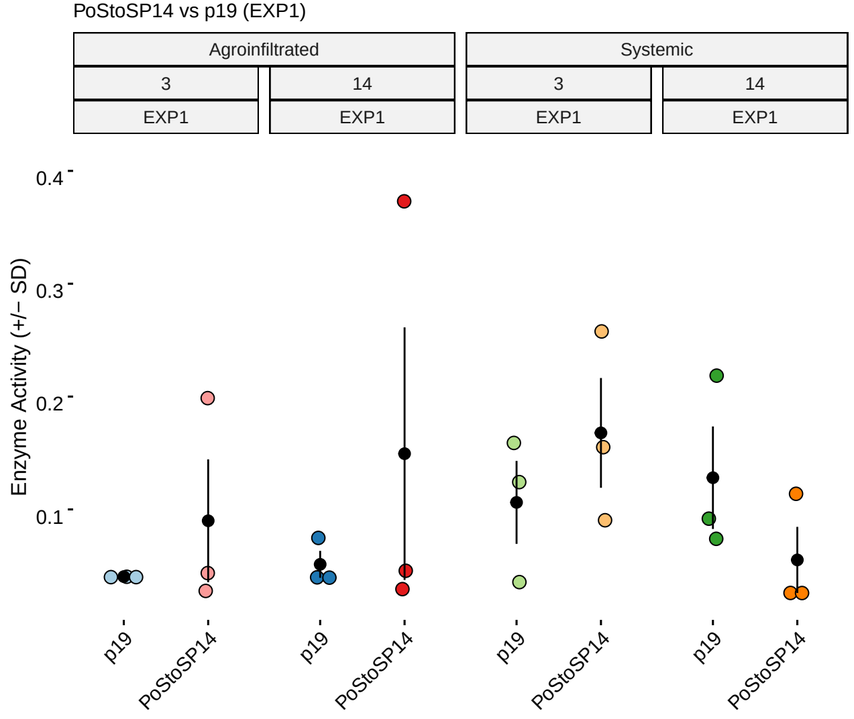

PoStoSP18 vs p19 (EXP1)

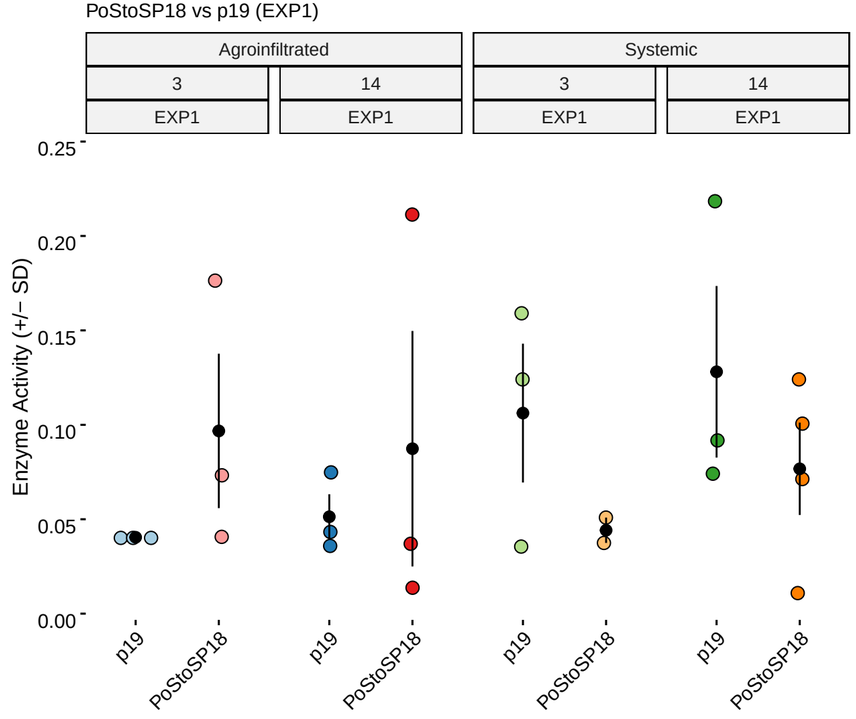

PoStoSP28 vs p19 (EXP1, EXP2)

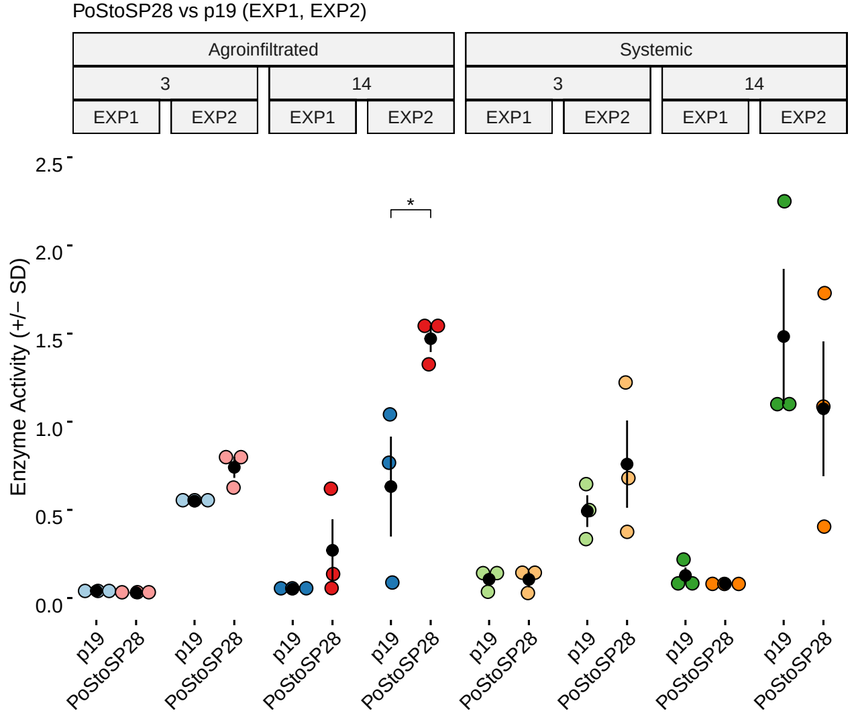

Dehydroascorbate reductase

PoStoSP04 vs p19 (EXP1)

Agroinfiltrated		Systemic	
3	14	3	14
EXP1	EXP1	EXP1	EXP1

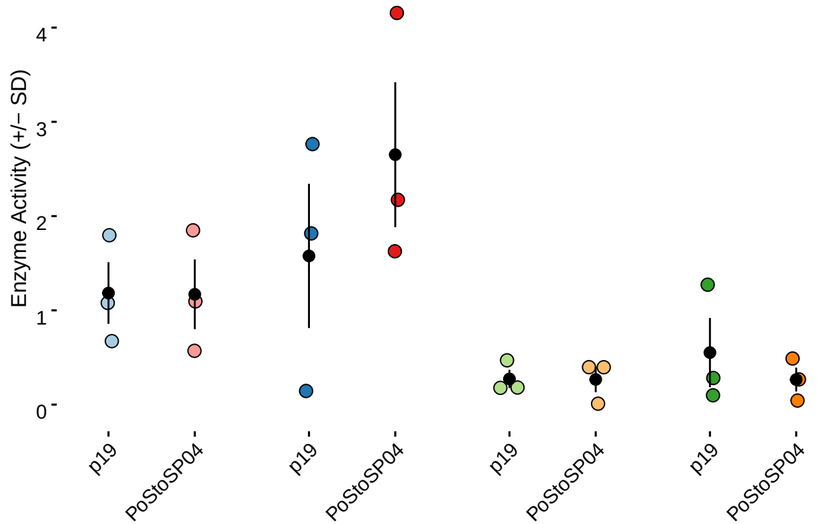

PoStoSP06 vs p19 (EXP1)

Agroinfiltrated		Systemic	
3	14	3	14
EXP1	EXP1	EXP1	EXP1

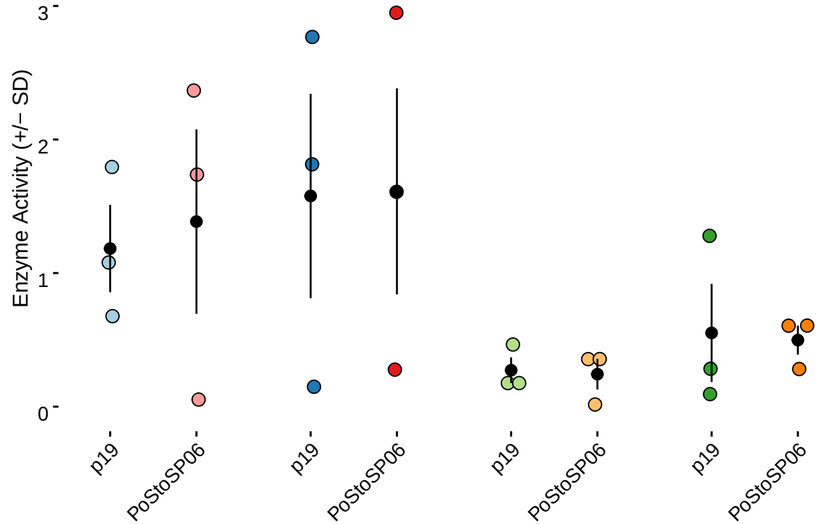

PoStoSP13 vs p19 (EXP1)

Agroinfiltrated		Systemic	
3	14	3	14
EXP1	EXP1	EXP1	EXP1

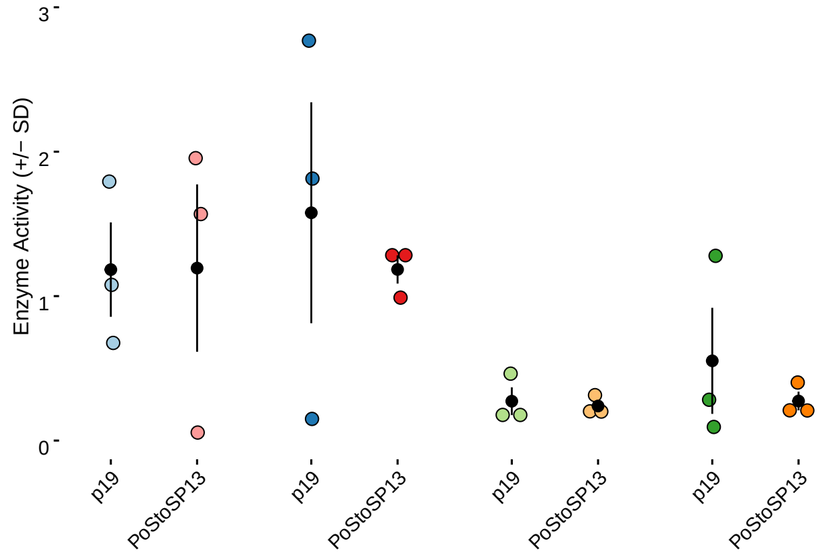

PoStoSP14 vs p19 (EXP1)

Agroinfiltrated		Systemic	
3	14	3	14
EXP1	EXP1	EXP1	EXP1

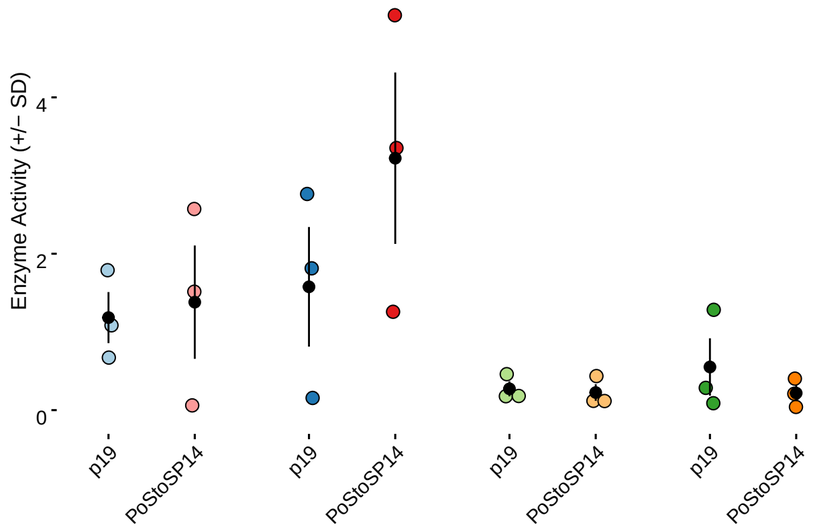

PoStoSP18 vs p19 (EXP1)

Agroinfiltrated		Systemic	
3	14	3	14
EXP1	EXP1	EXP1	EXP1

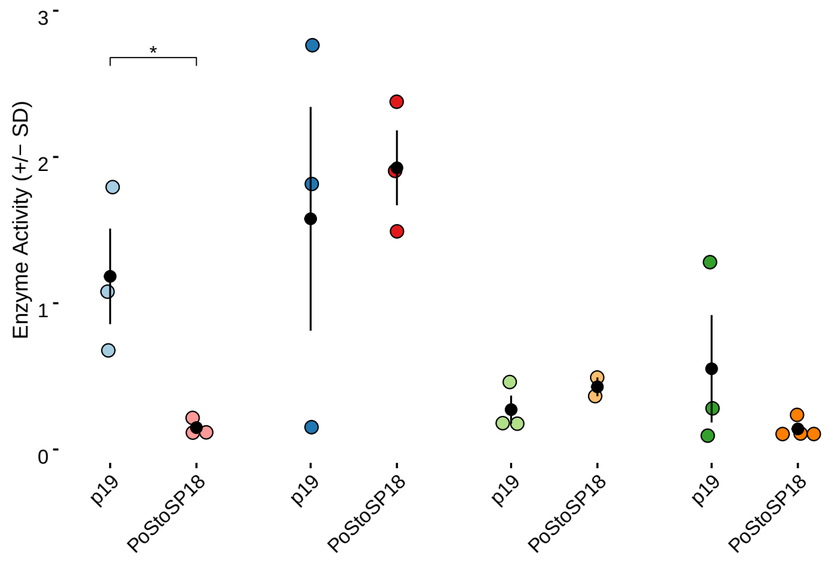

PoStoSP28 vs p19 (EXP1)

Agroinfiltrated		Systemic	
3	14	3	14
EXP1	EXP1	EXP1	EXP1

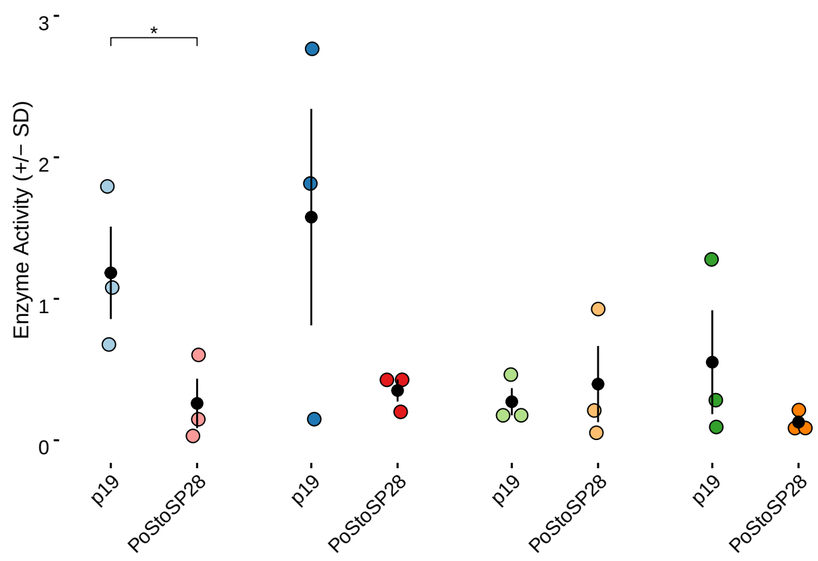

Glutathione reductase

PoStoSP04 vs p19 (EXP1)

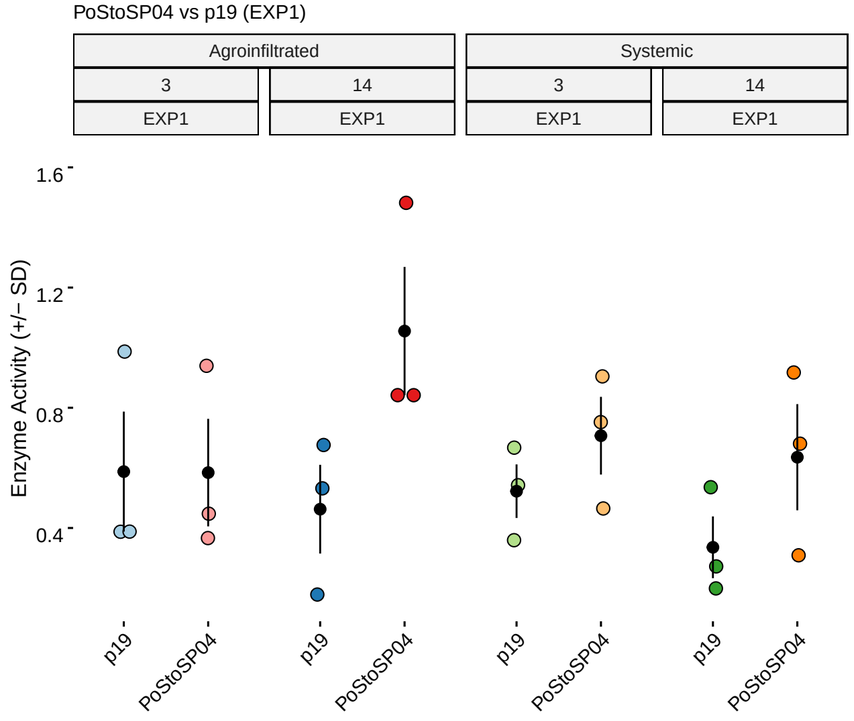

PoStoSP06 vs p19 (EXP1, EXP2)

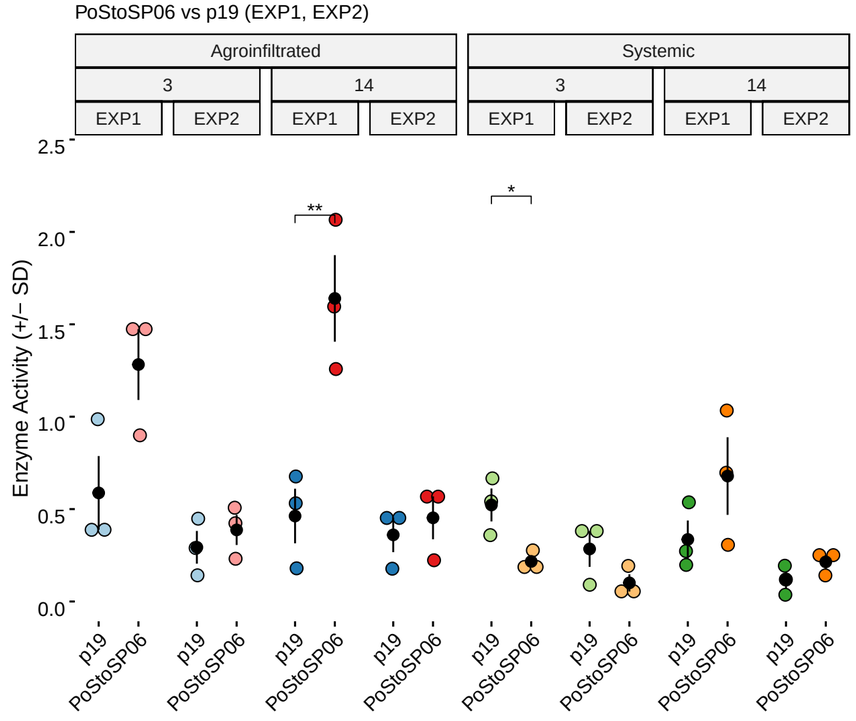

PoStoSP13 vs p19 (EXP1, EXP2)

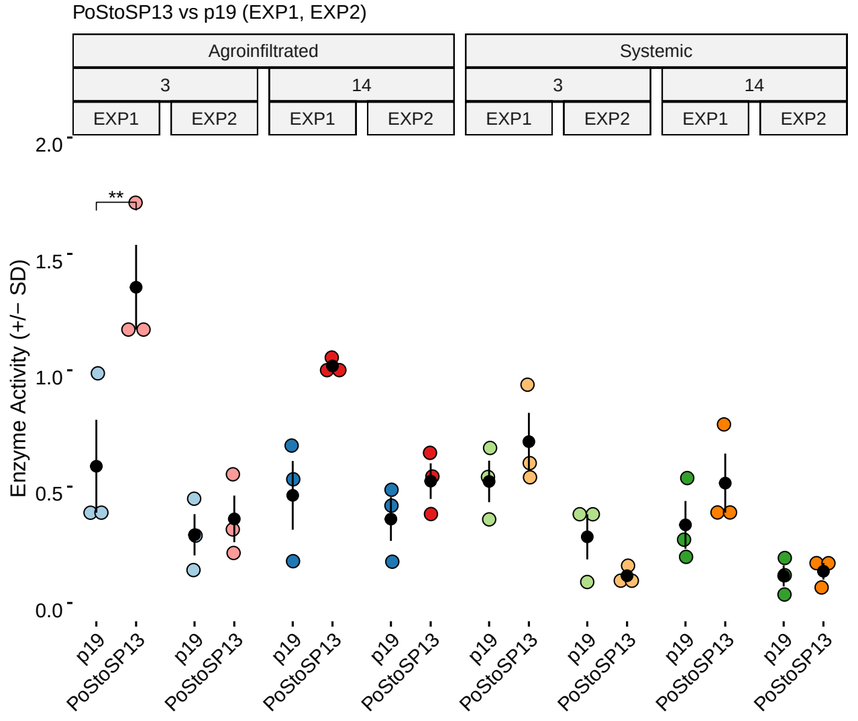

PoStoSP14 vs p19 (EXP1)

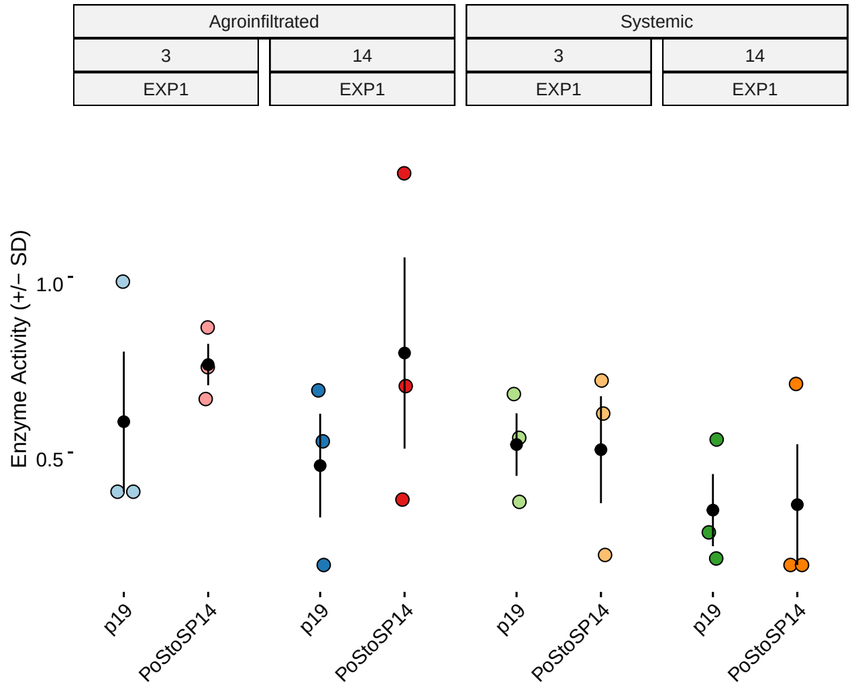

PoStoSP18 vs p19 (EXP1)

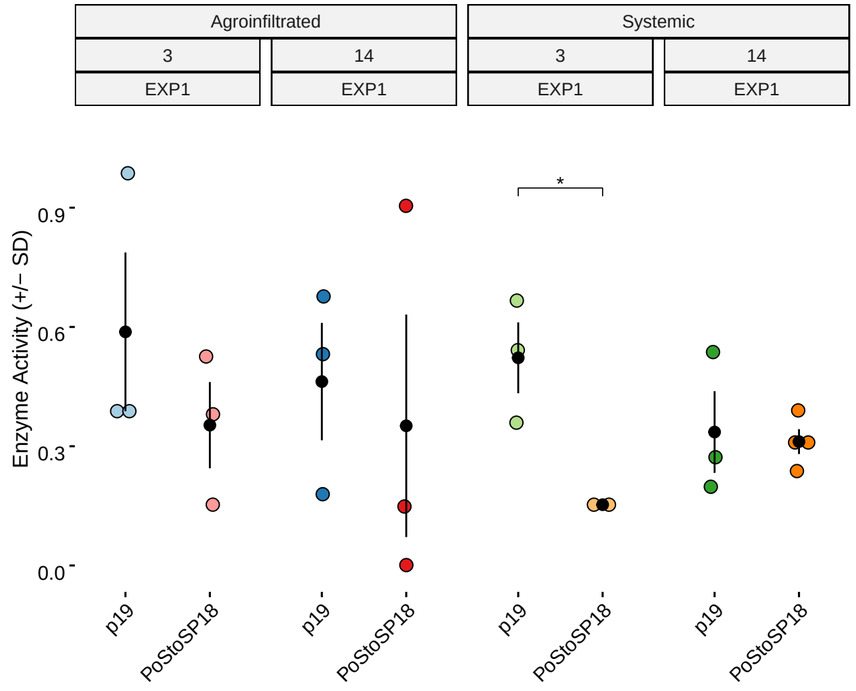

PoStoSP28 vs p19 (EXP1, EXP2)

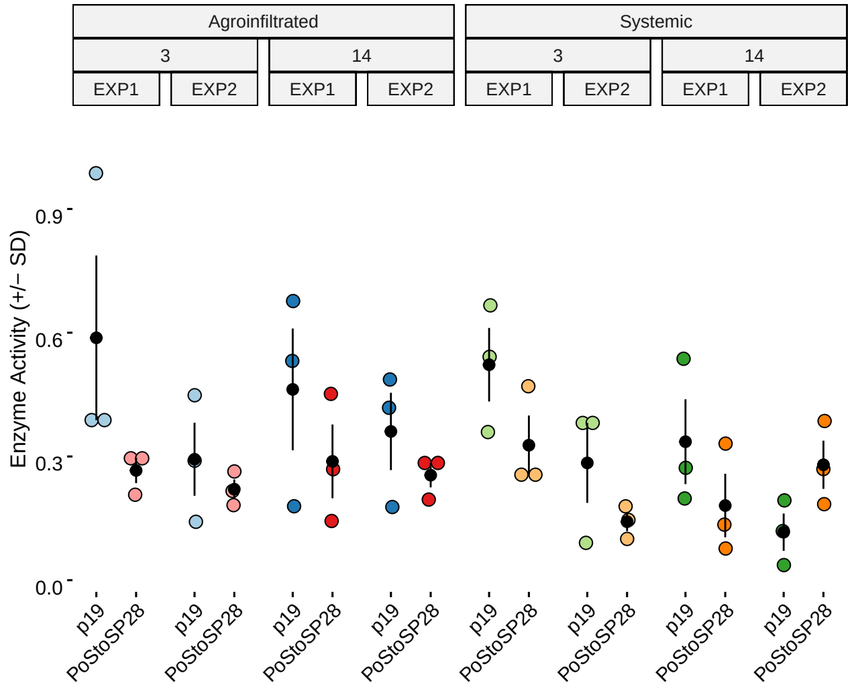

Glutathione S-transferases

PoStoSP06 vs p19 (EXP1)

Agroinfiltrated		Systemic	
3	14	3	14
EXP1	EXP1	EXP1	EXP1

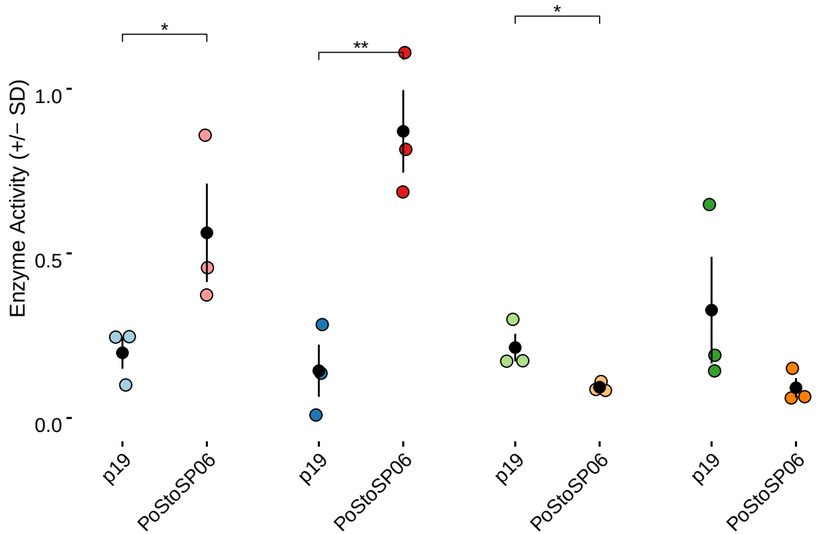

PoStoSP13 vs p19 (EXP1)

Agroinfiltrated		Systemic	
3	14	3	14
EXP1	EXP1	EXP1	EXP1

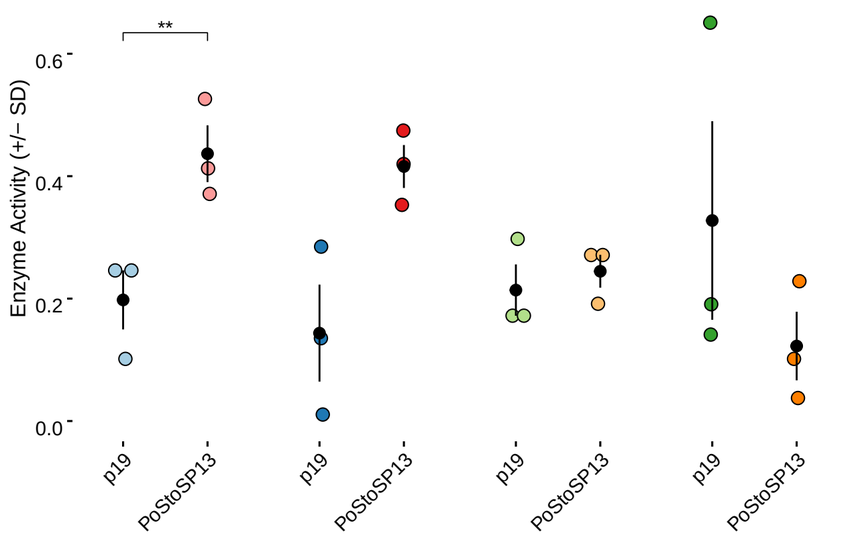

PoStoSP28 vs p19 (EXP1)

Agroinfiltrated		Systemic	
3	14	3	14
EXP1	EXP1	EXP1	EXP1

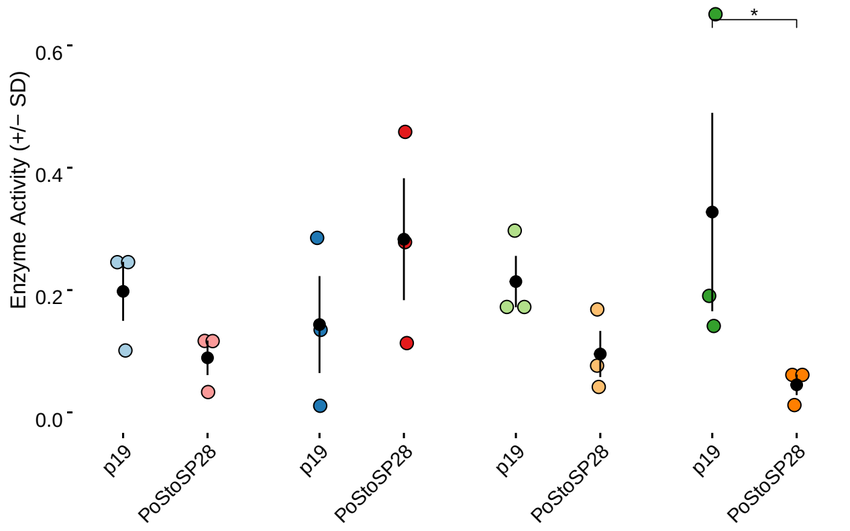

Monodehydroascorbate reductase

PoStoSP04 vs p19 (EXP1)

Agroinfiltrated		Systemic	
3	14	3	14
EXP1	EXP1	EXP1	EXP1

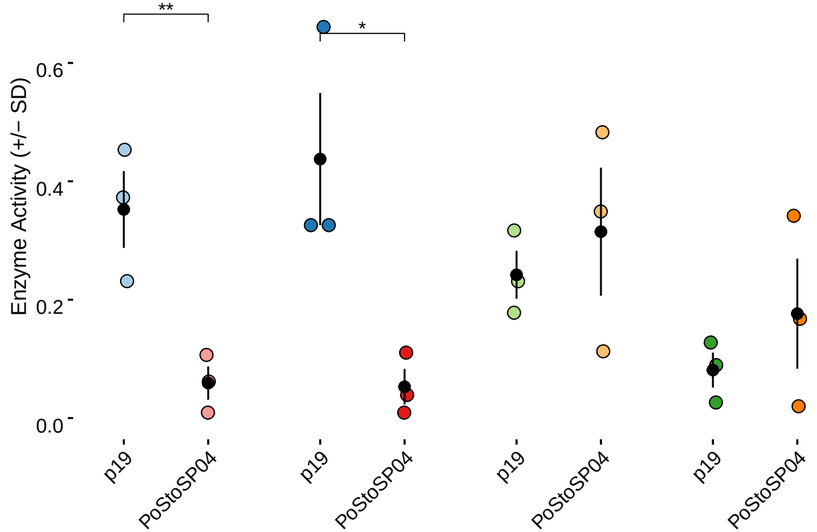

PoStoSP06 vs p19 (EXP1)

Agroinfiltrated		Systemic	
3	14	3	14
EXP1	EXP1	EXP1	EXP1

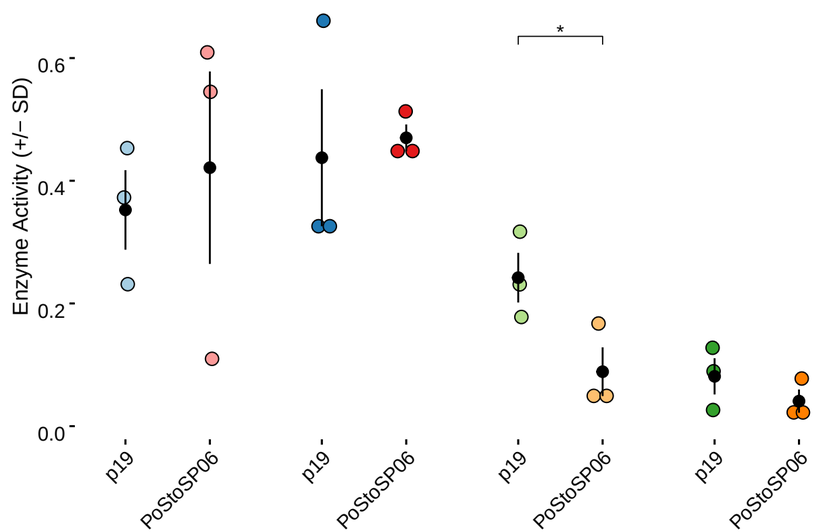

PoStoSP13 vs p19 (EXP1)

Agroinfiltrated		Systemic	
3	14	3	14
EXP1	EXP1	EXP1	EXP1

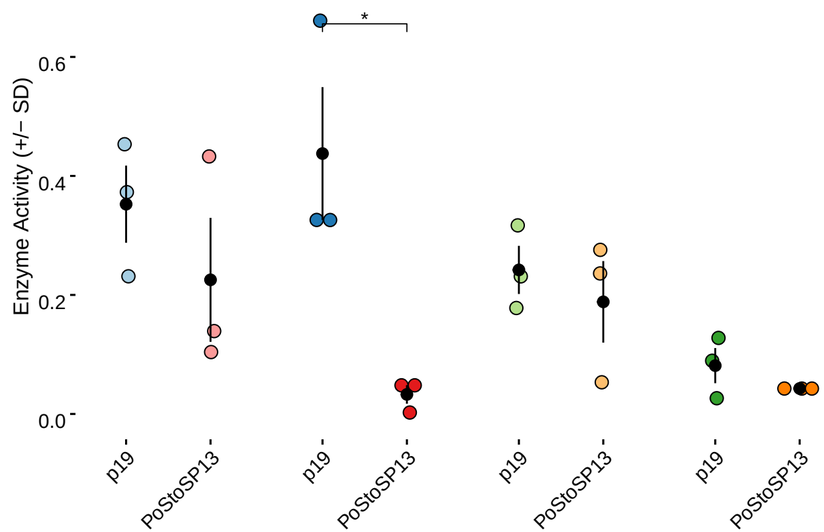

PoStoSP14 vs p19 (EXP1)

Agroinfiltrated		Systemic	
3	14	3	14
EXP1	EXP1	EXP1	EXP1

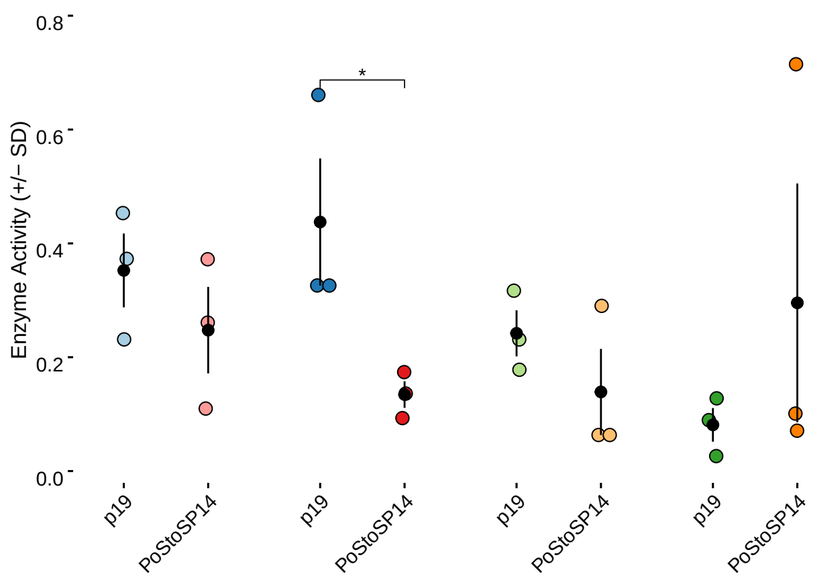

PoStoSP28 vs p19 (EXP1)

Agroinfiltrated		Systemic	
3	14	3	14
EXP1	EXP1	EXP1	EXP1

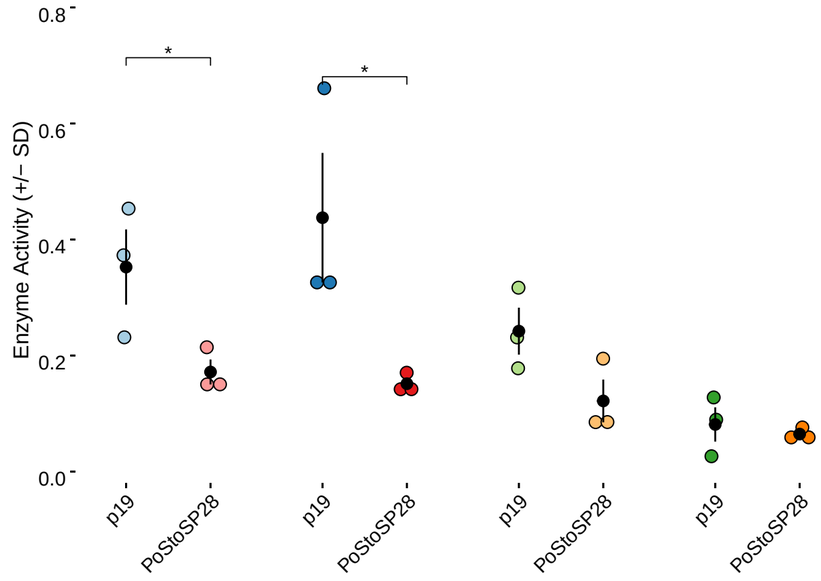

Peroxidase

PoStoSP04 vs p19 (EXP1)

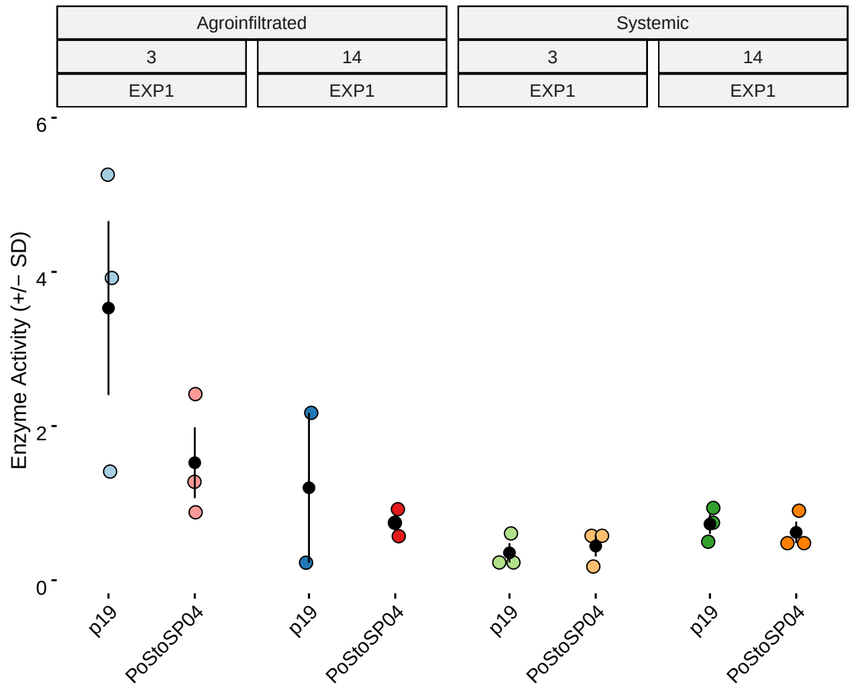

PoStoSP06 vs p19 (EXP2)

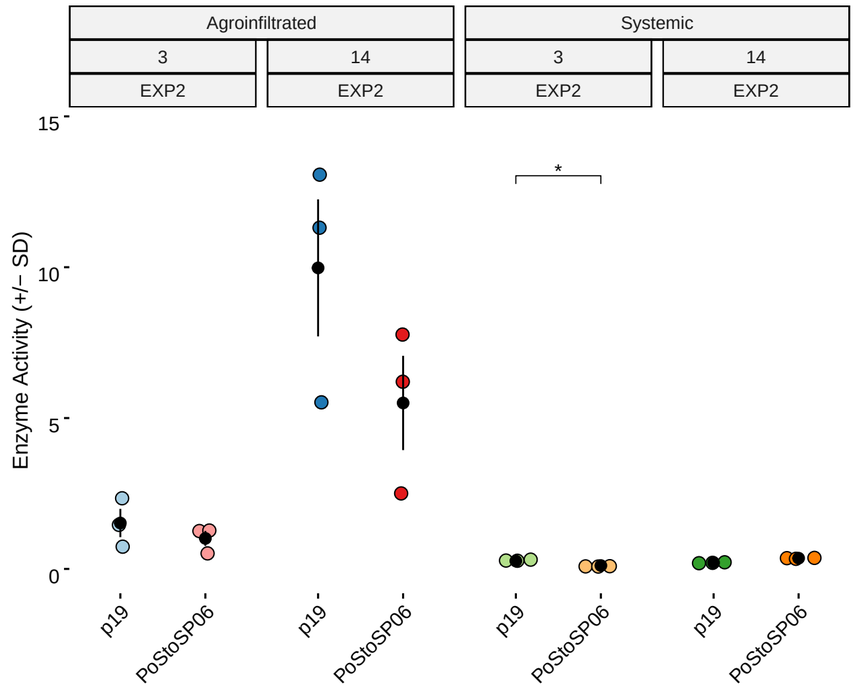

PoStoSP13 vs p19 (EXP1, EXP2)

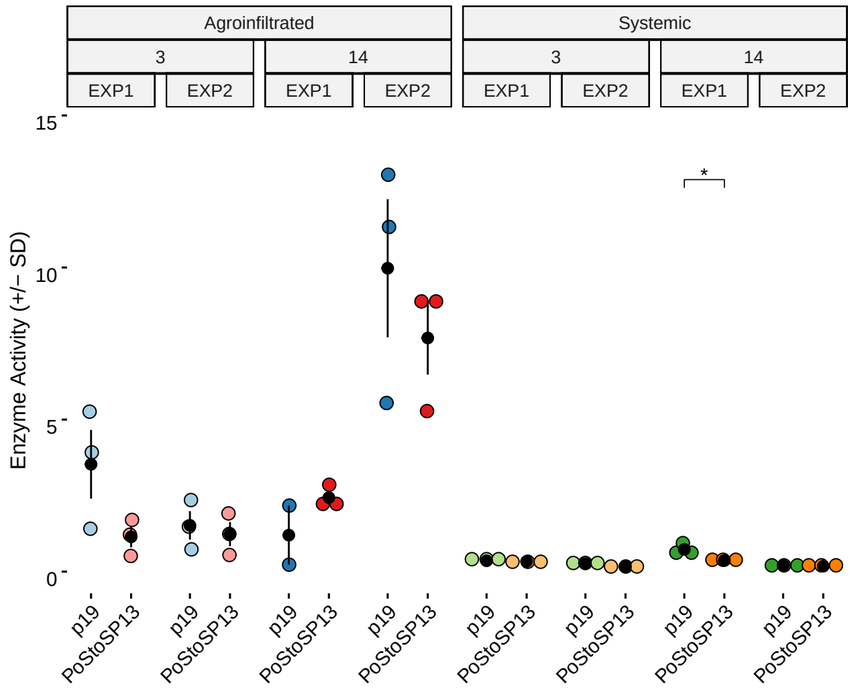

PoStoSP14 vs p19 (EXP1)

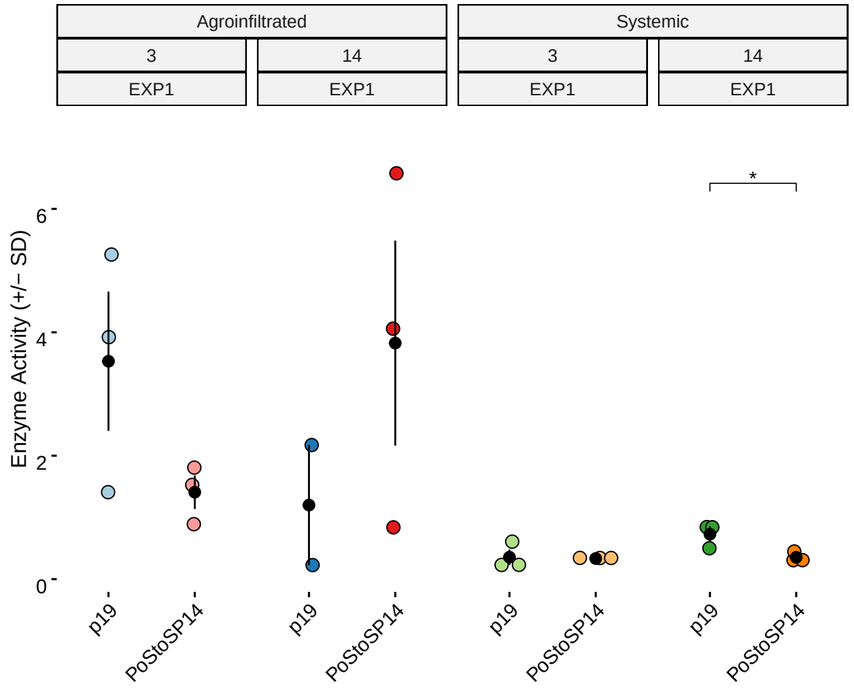

PoStoSP18 vs p19 (EXP1)

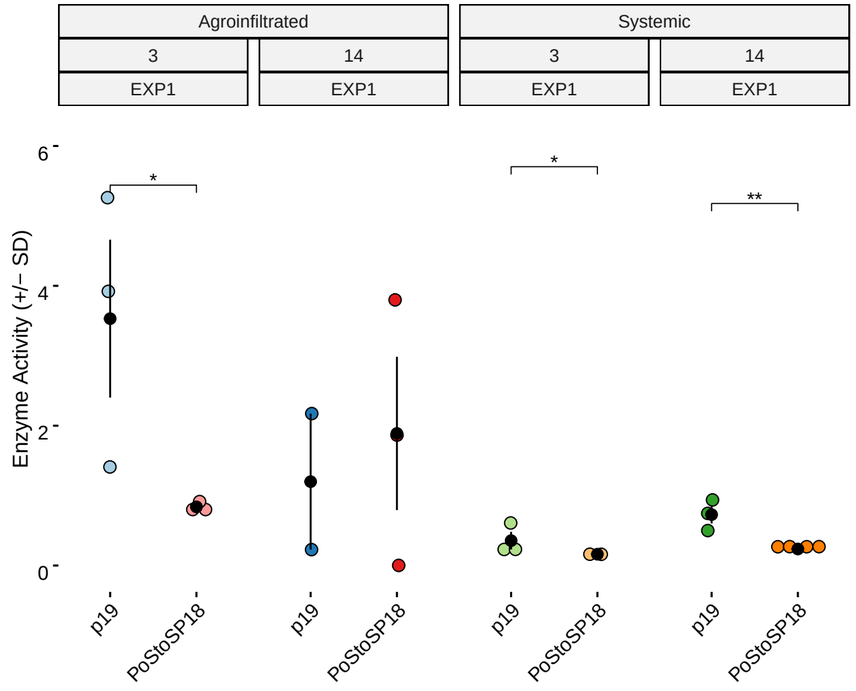

PoStoSP28 vs p19 (EXP1, EXP2)

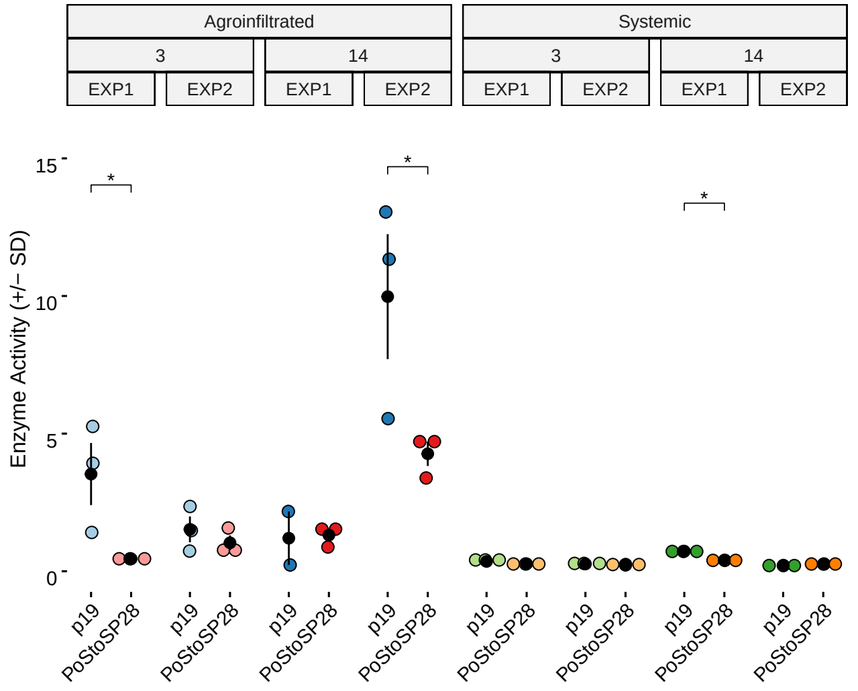

Superoxide dismutase

PoStoSP04 vs p19 (EXP1)

Agroinfiltrated		Systemic	
3	14	3	14
EXP1	EXP1	EXP1	EXP1

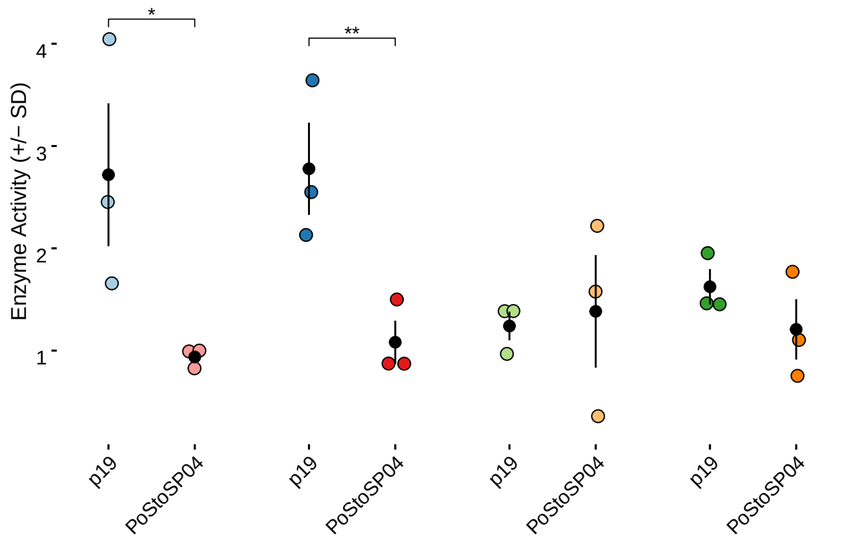

PoStoSP06 vs p19 (EXP1)

Agroinfiltrated		Systemic	
3	14	3	14
EXP1	EXP1	EXP1	EXP1

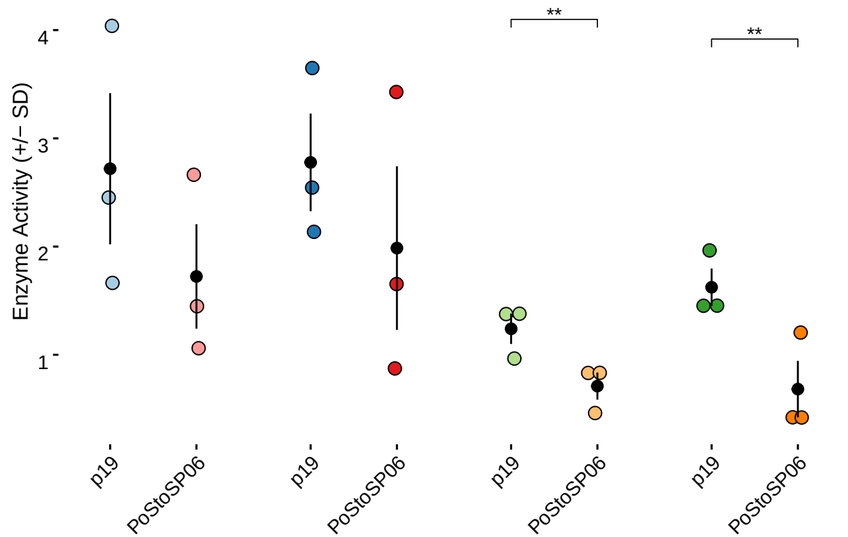

PoStoSP13 vs p19 (EXP1)

Agroinfiltrated		Systemic	
3	14	3	14
EXP1	EXP1	EXP1	EXP1

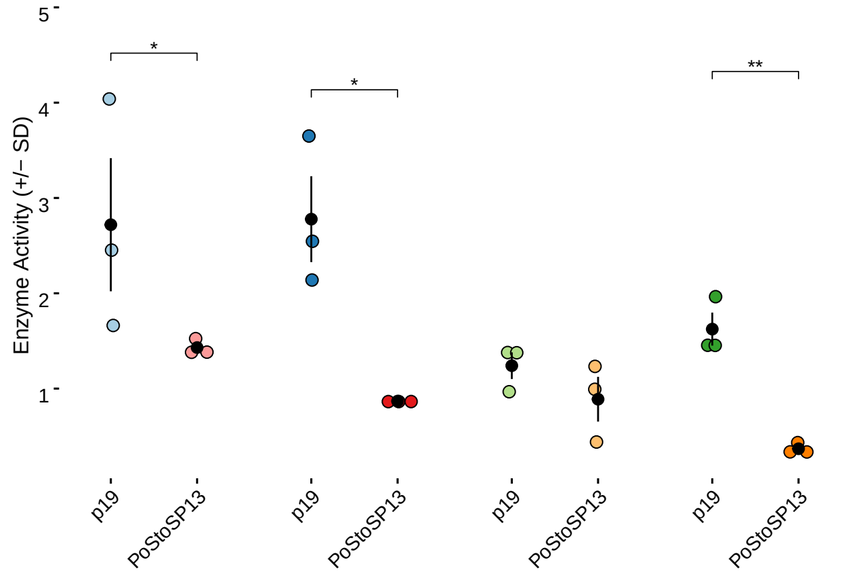

PoStoSP14 vs p19 (EXP1)

Agroinfiltrated		Systemic	
3	14	3	14
EXP1	EXP1	EXP1	EXP1

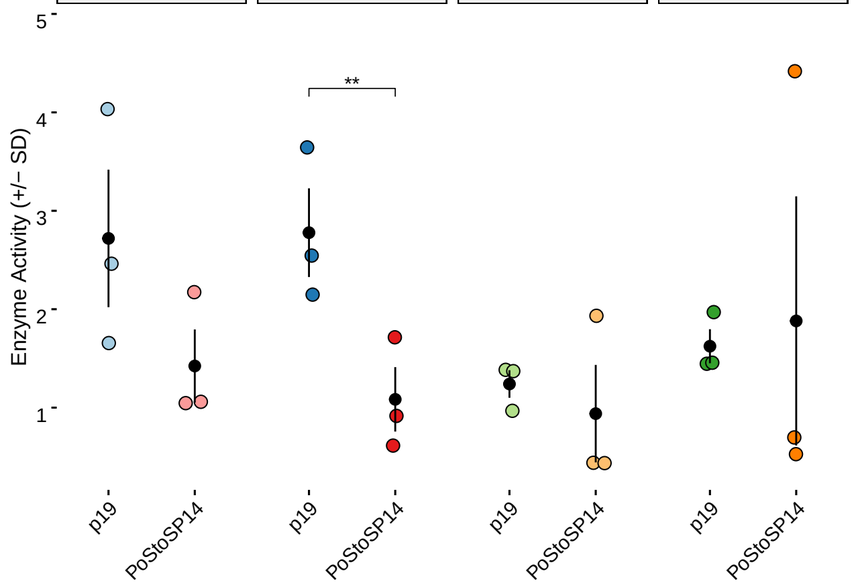

PoStoSP18 vs p19 (EXP1)

Agroinfiltrated		Systemic	
3	14	3	14
EXP1	EXP1	EXP1	EXP1

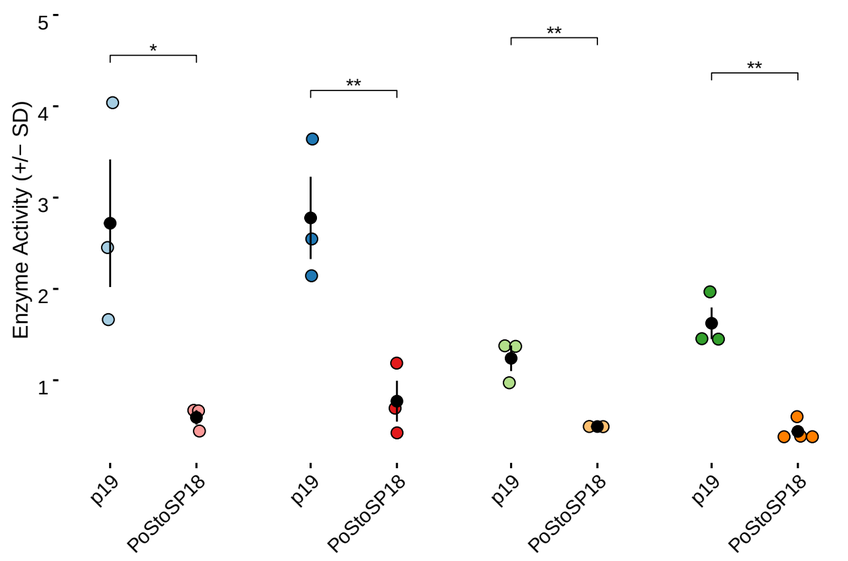

PoStoSP28 vs p19 (EXP1)

Agroinfiltrated		Systemic	
3	14	3	14
EXP1	EXP1	EXP1	EXP1

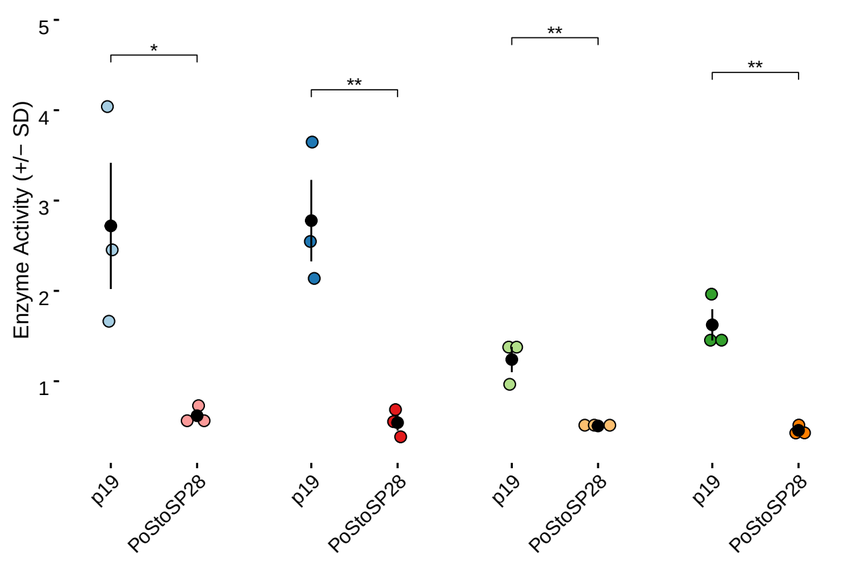

Supplement: Supplementary file 1 [file DataSheet_1.pdf]
