# Supplementary material for: Candidate pathogenicity factor/effector proteins of ‘Candidatus Phytoplasma solani’ modulate plant carbohydrate metabolism, accelerate the ascorbate–glutathione cycle, and induce autophagosomes
Source: Front Plant Sci. 2023 Aug 18;14:1232367. doi: 10.3389/fpls.2023.1232367 (PMC10471893; doi:10.3389/fpls.2023.1232367)
Supplement: Supplementary file 2 [file DataSheet_2.pdf]

Supplemental Information

Title: Candidate pathogenicity factor/effector proteins of ‘*Candidatus* Phytoplasma solani’ modulate plant carbohydrate metabolism, accelerate the ascorbate-glutathione cycle and induce autophagosomes

Authors: Marina Dermastia*, Špela Tomaž, Rebeka Strah, Tjaša Lukan, Anna Coll, Barbara Dušak, Barbara Anžič, Timotej Čepin, Stefanie Wienkoop, Aleš Kladnik, Maja Zagorščak, Monika Riedle-Bauer, Christina Schönhuber, Wolfram Weckwerth, Kristina Gruden, Thomas Roitsch, Maruša Pompe Novak, Günter Brader

* Correspondence: marina.dermastia@nib.si

Supplemental Figure S2. Tukey's test

Vitis vinifera cv. ‘Zweigelt’ infected with ‘*Ca*. P. solani’

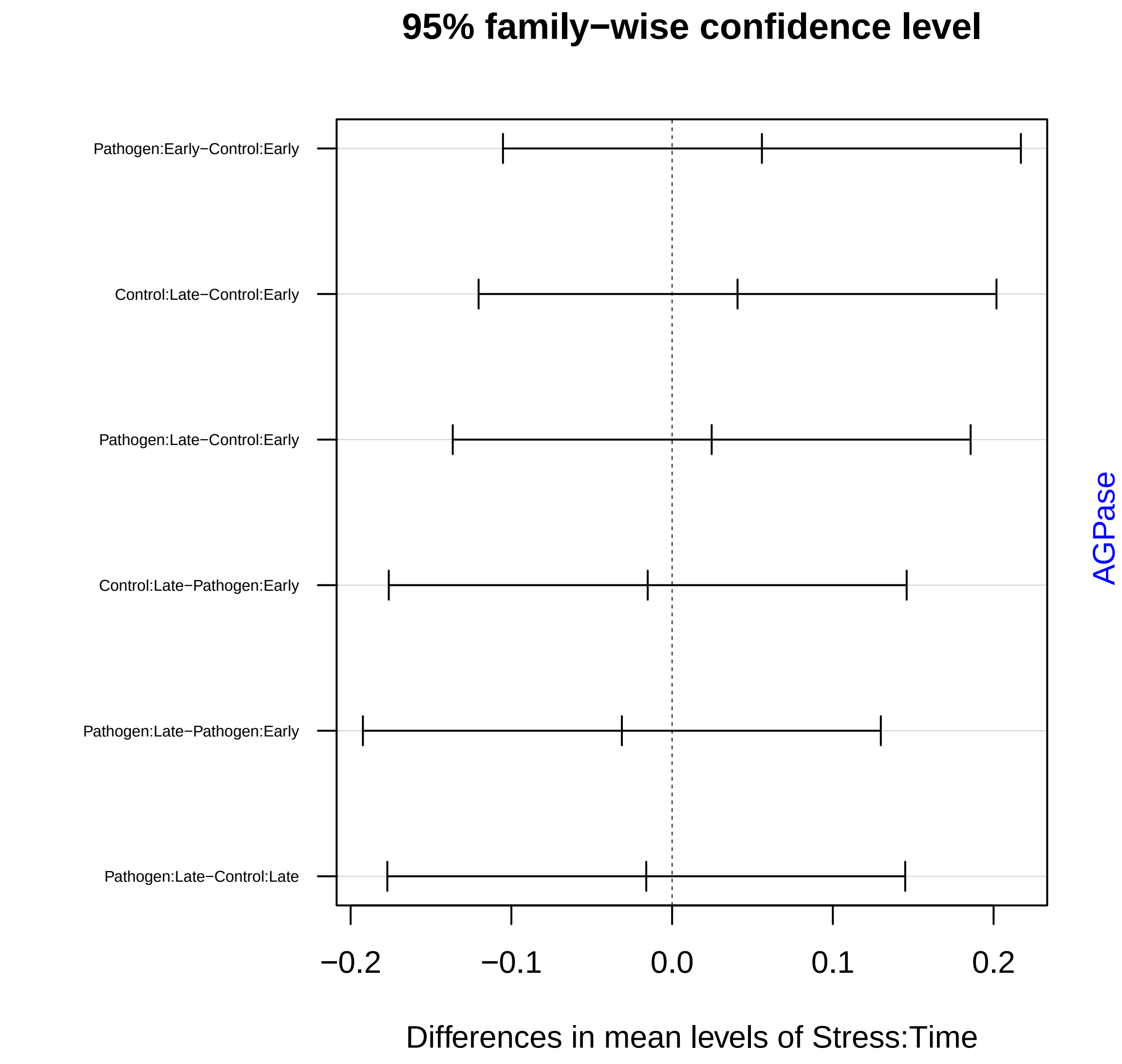
95% family−wise confidence level
Pathogen:Early−Control:Early
Control:Late−Control:Early
Pathogen:Late−Control:Early
Control:Late−Pathogen:Early
Pathogen:Late−Pathogen:Early
Pathogen:Late−Control:Late
−0.2
−0.1
0.0
0.1
0.2
Differences in mean levels of Stress:Time
AGPase

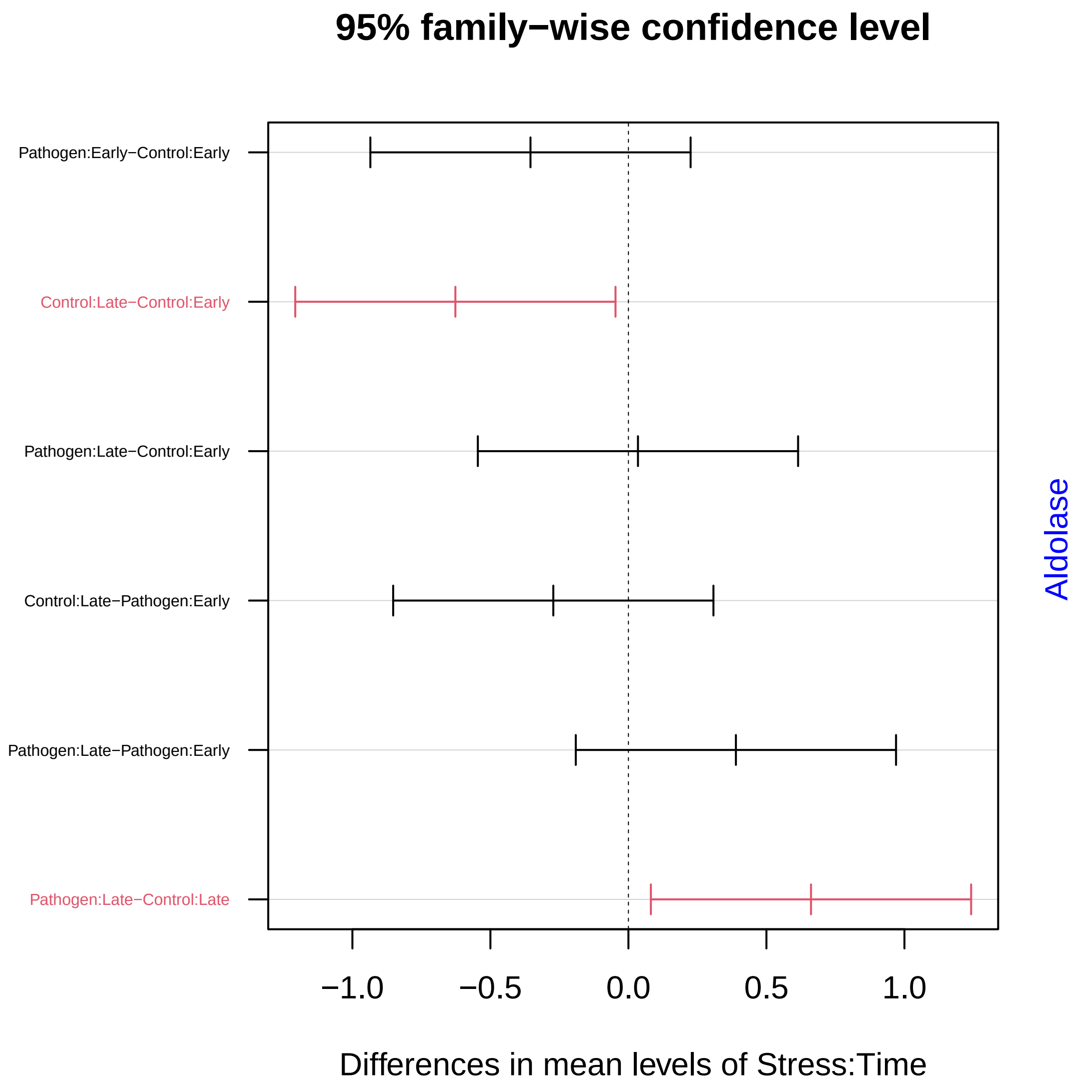
95% family−wise confidence level
Pathogen:Early−Control:Early
Control:Late−Control:Early
Pathogen:Late−Control:Early
Control:Late−Pathogen:Early
Pathogen:Late−Pathogen:Early
Pathogen:Late−Control:Late
−1.0
−0.5
0.0
0.5
1.0
Differences in mean levels of Stress:Time
Aldolase

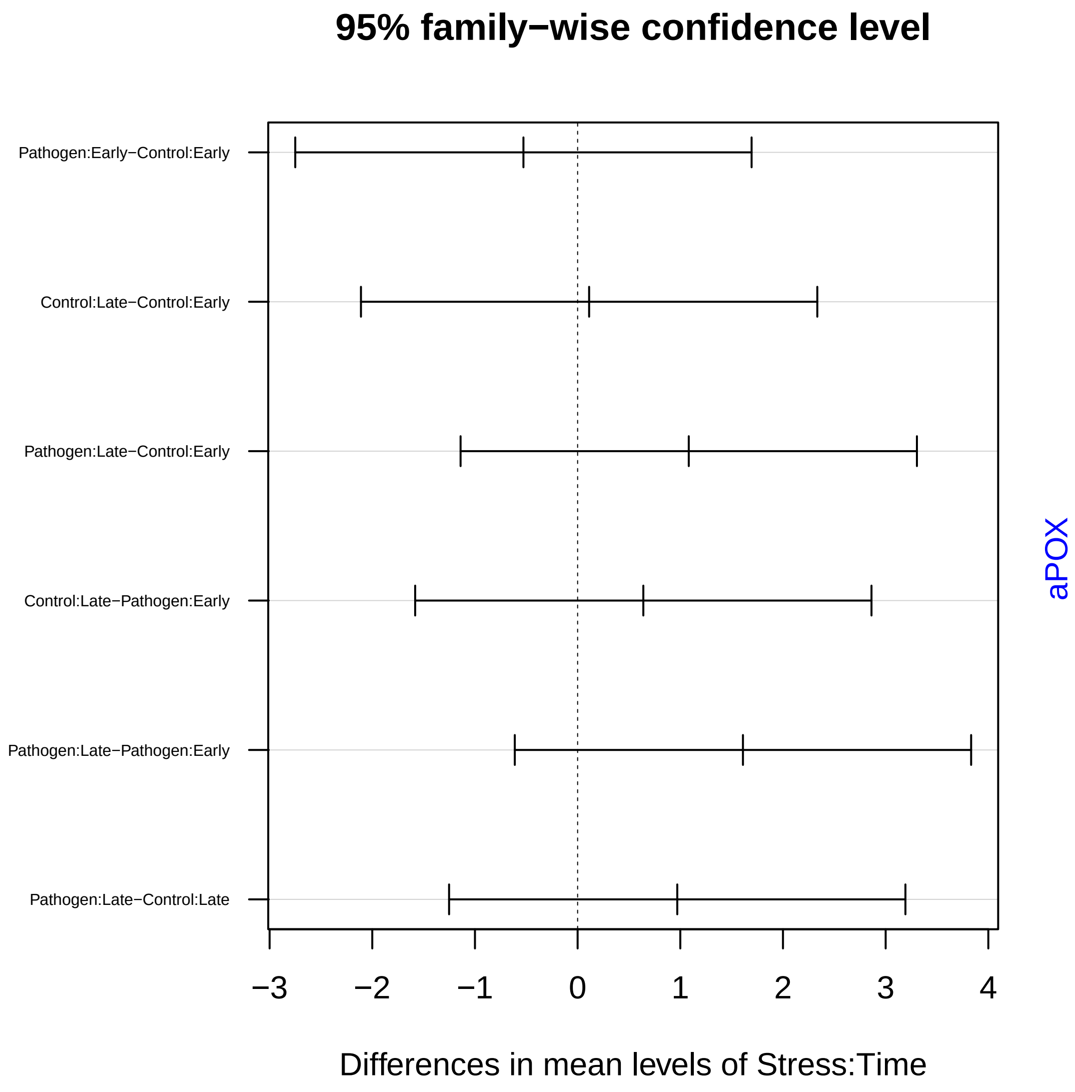

95% family−wise confidence level
Pathogen:Early−Control:Early
Control:Late−Control:Early
Pathogen:Late−Control:Early
Control:Late−Pathogen:Early
Pathogen:Late−Pathogen:Early
Pathogen:Late−Control:Late
−3
−2
−1
0
1
2
3
4
Differences in mean levels of Stress:Time
aPOX

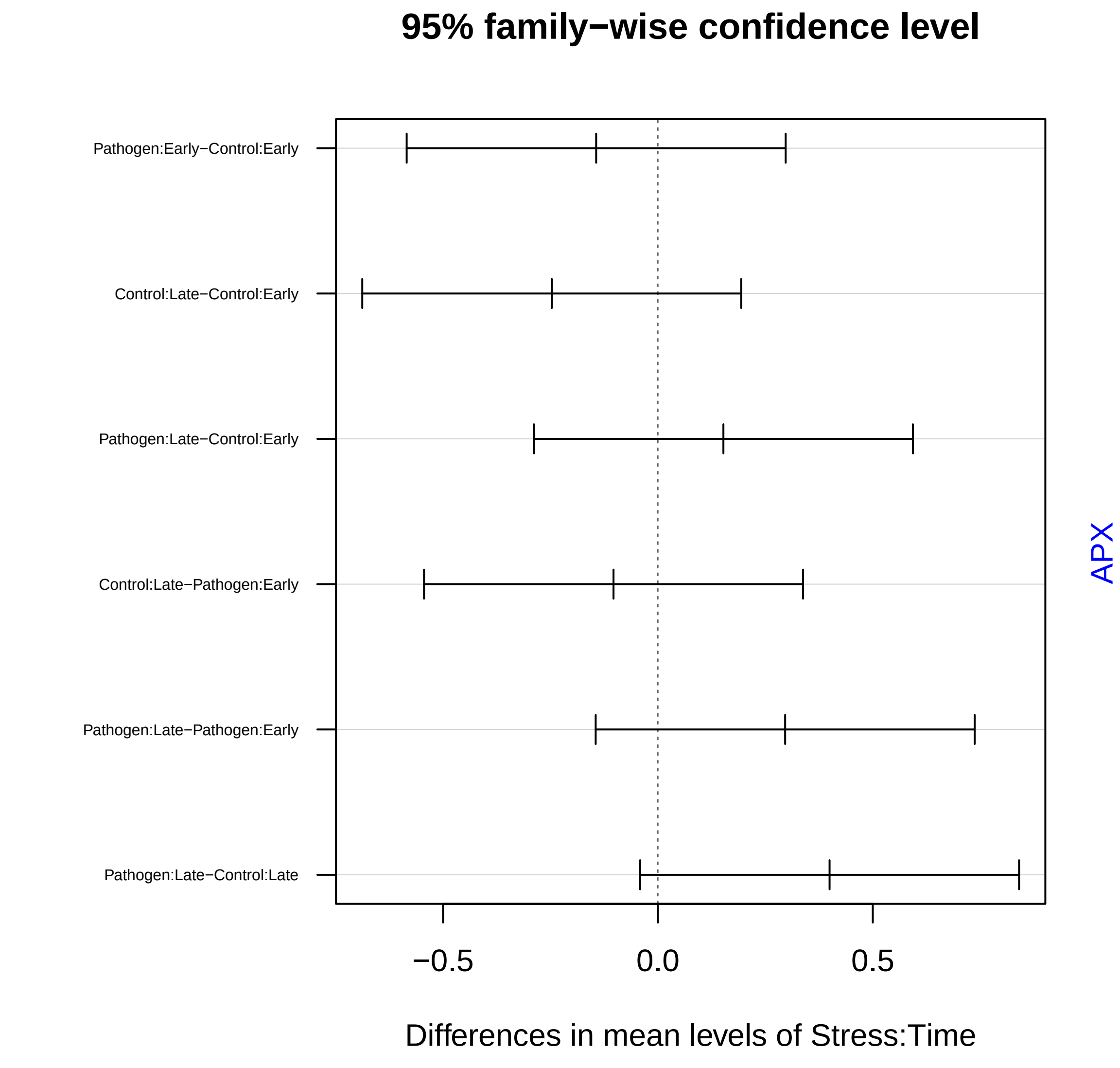
95% family−wise confidence level
Pathogen:Early−Control:Early
Control:Late−Control:Early
Pathogen:Late−Control:Early
Control:Late−Pathogen:Early
Pathogen:Late−Pathogen:Early
Pathogen:Late−Control:Late
−0.5
0.0
0.5
Differences in mean levels of Stress:Time
APX

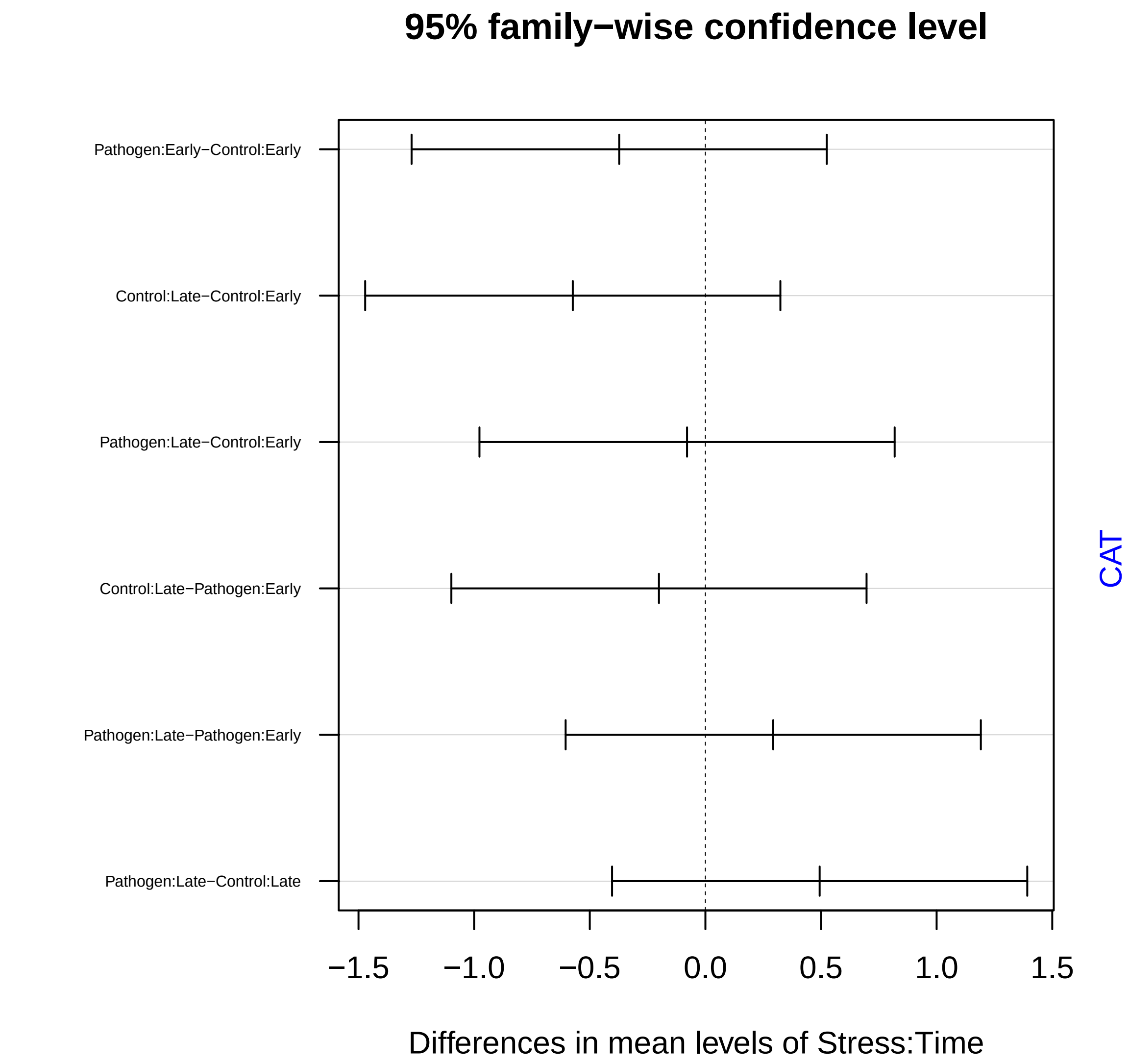

95% family−wise confidence level
Pathogen:Early−Control:Early
Control:Late−Control:Early
Pathogen:Late−Control:Early
Control:Late−Pathogen:Early
Pathogen:Late−Pathogen:Early
Pathogen:Late−Control:Late
−1.5
−1.0
−0.5
0.0
0.5
1.0
1.5
Differences in mean levels of Stress:Time
CAT

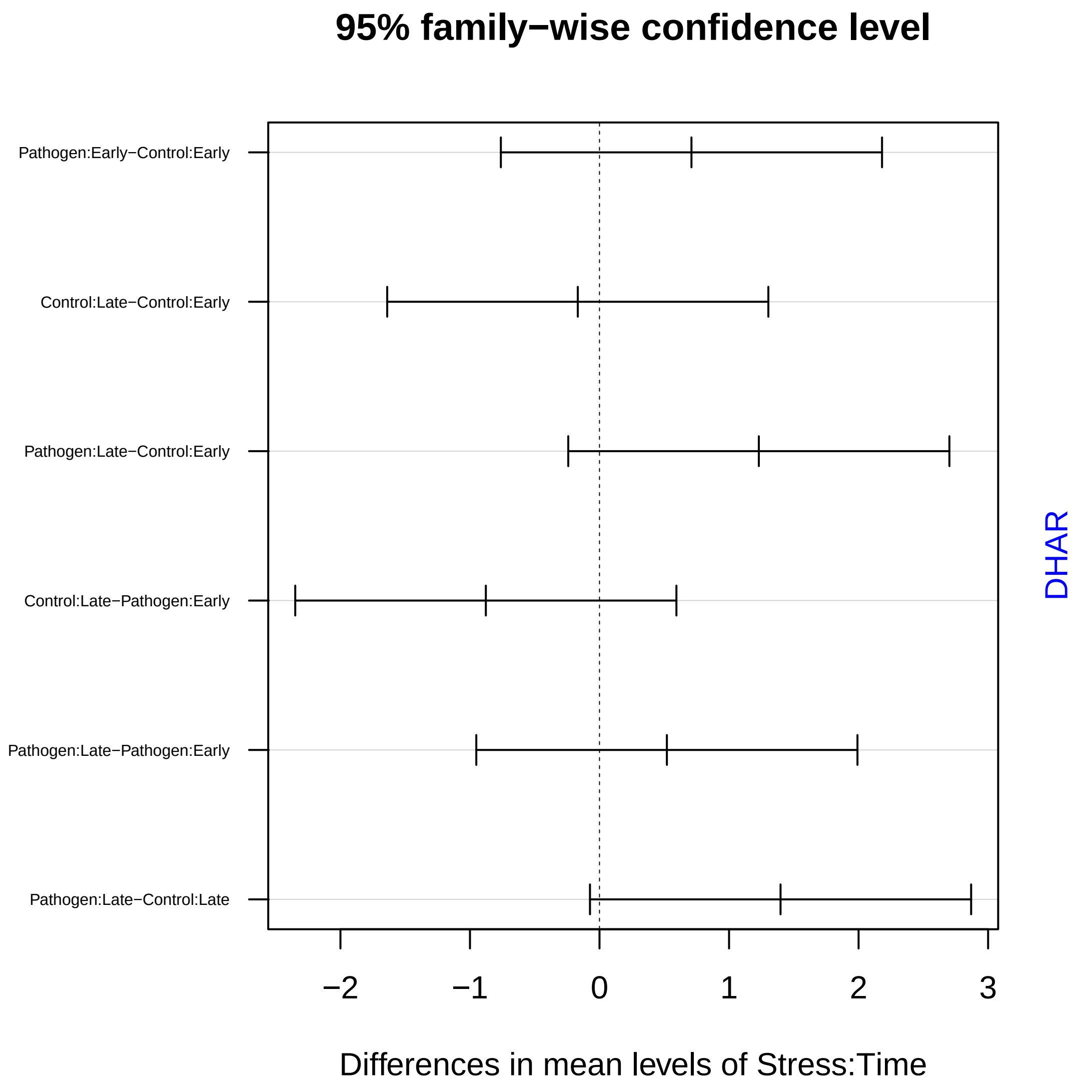
95% family−wise confidence level
Pathogen:Early−Control:Early
Control:Late−Control:Early
Pathogen:Late−Control:Early
Control:Late−Pathogen:Early
Pathogen:Late−Pathogen:Early
Pathogen:Late−Control:Late
−2
−1
0
1
2
3
Differences in mean levels of Stress:Time
DHAR

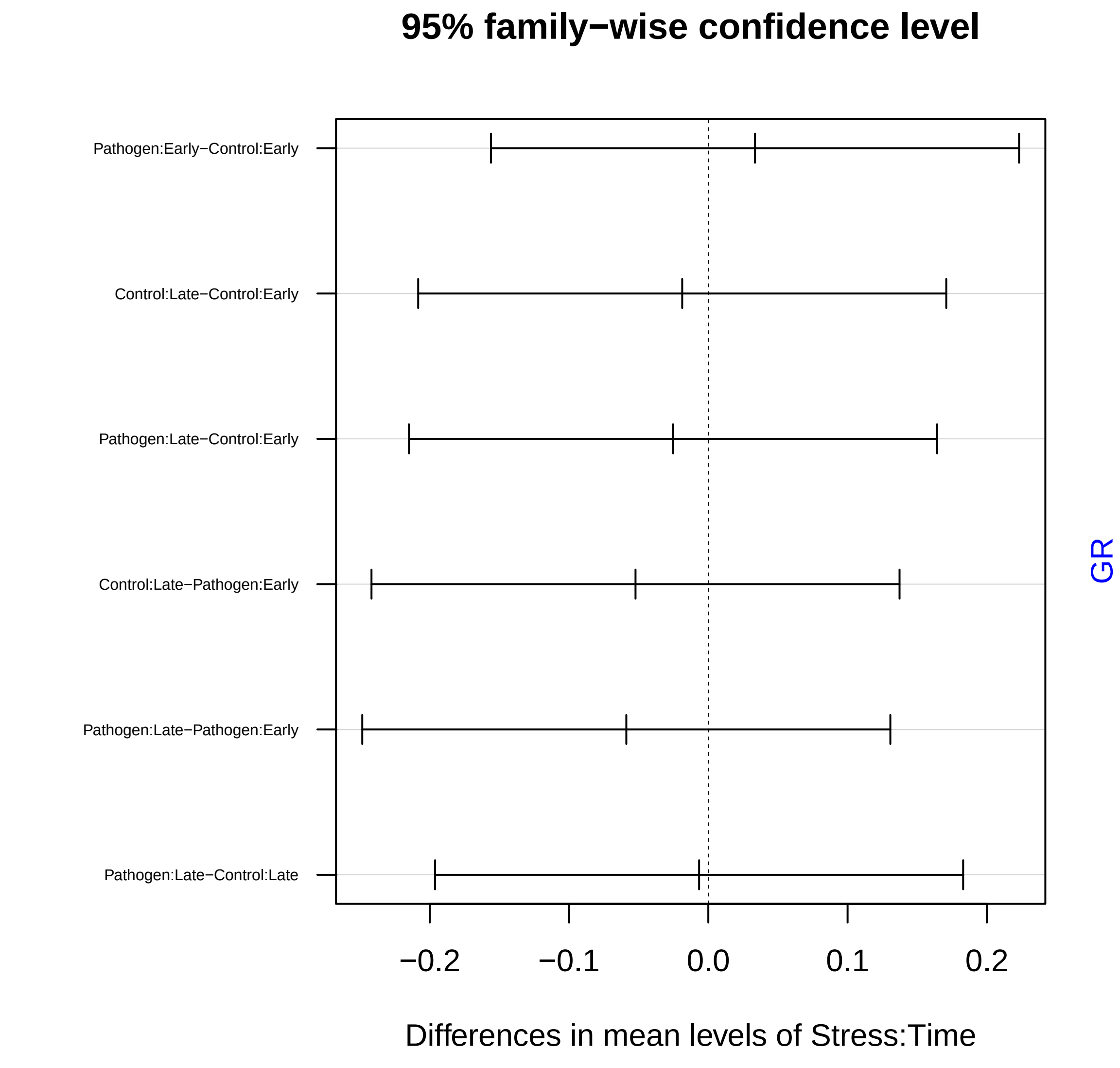

95% family−wise confidence level
Pathogen:Early−Control:Early
Control:Late−Control:Early
Pathogen:Late−Control:Early
Control:Late−Pathogen:Early
Pathogen:Late−Pathogen:Early
Pathogen:Late−Control:Late
−0.2
−0.1
0.0
0.1
0.2
Differences in mean levels of Stress:Time
GR

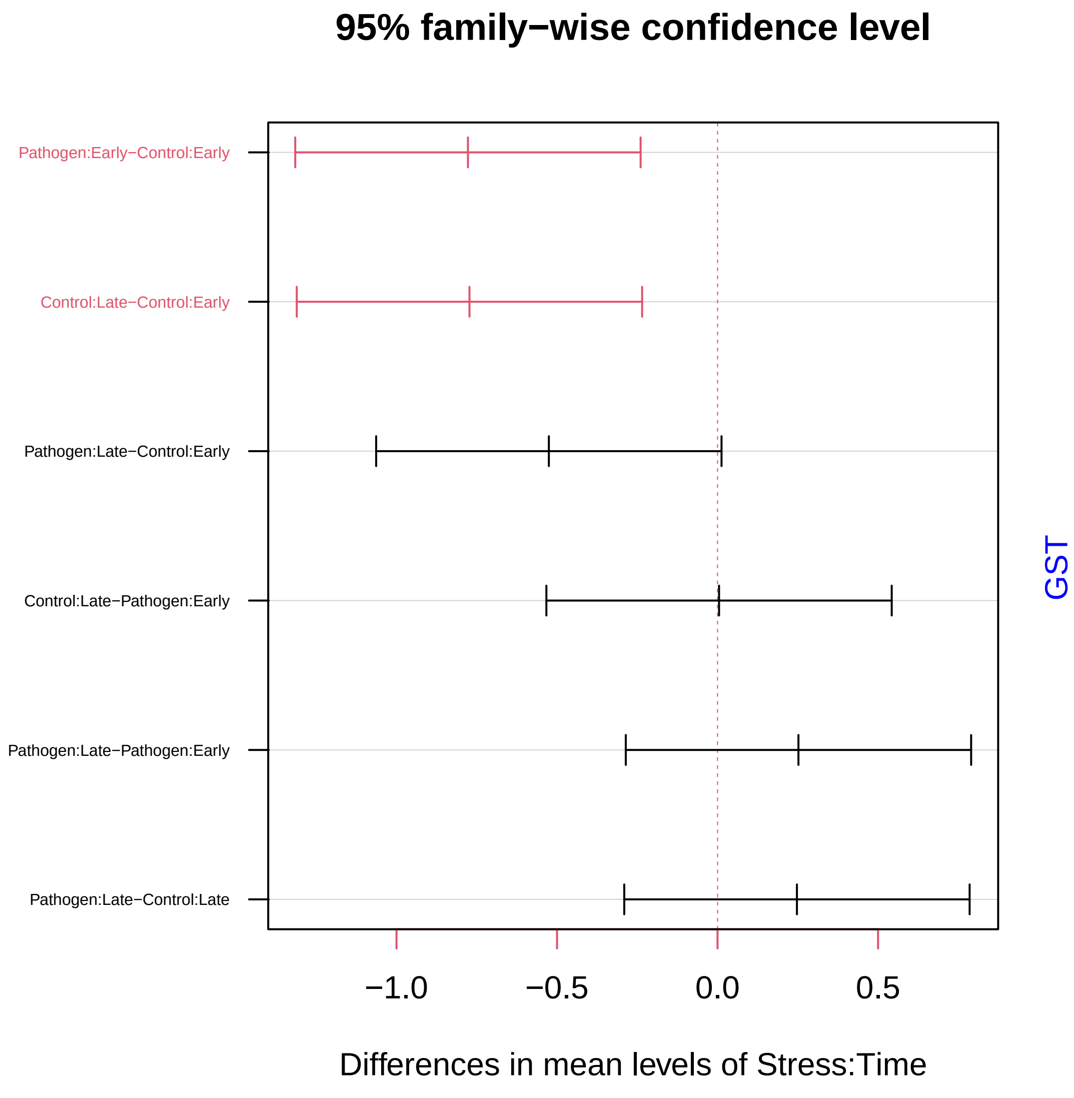

95% family−wise confidence level
Pathogen:Early−Control:Early
Control:Late−Control:Early
Pathogen:Late−Control:Early
Control:Late−Pathogen:Early
Pathogen:Late−Pathogen:Early
Pathogen:Late−Control:Late
−1.0
−0.5
0.0
0.5
Differences in mean levels of Stress:Time
GST

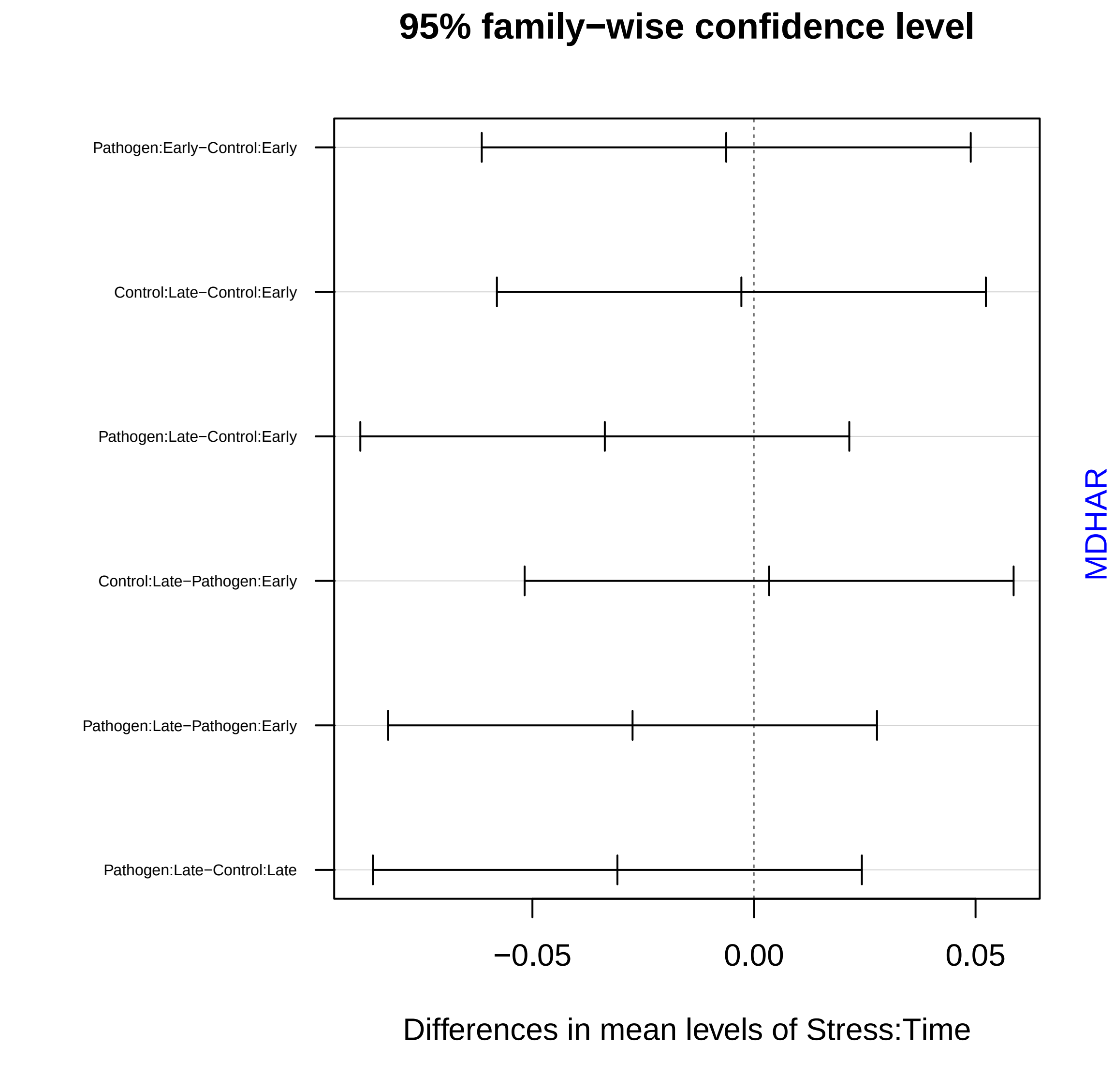

95% family−wise confidence level
Pathogen:Early−Control:Early
Control:Late−Control:Early
Pathogen:Late−Control:Early
Control:Late−Pathogen:Early
Pathogen:Late−Pathogen:Early
Pathogen:Late−Control:Late
−0.05
0.00
0.05
Differences in mean levels of Stress:Time
MDHAR

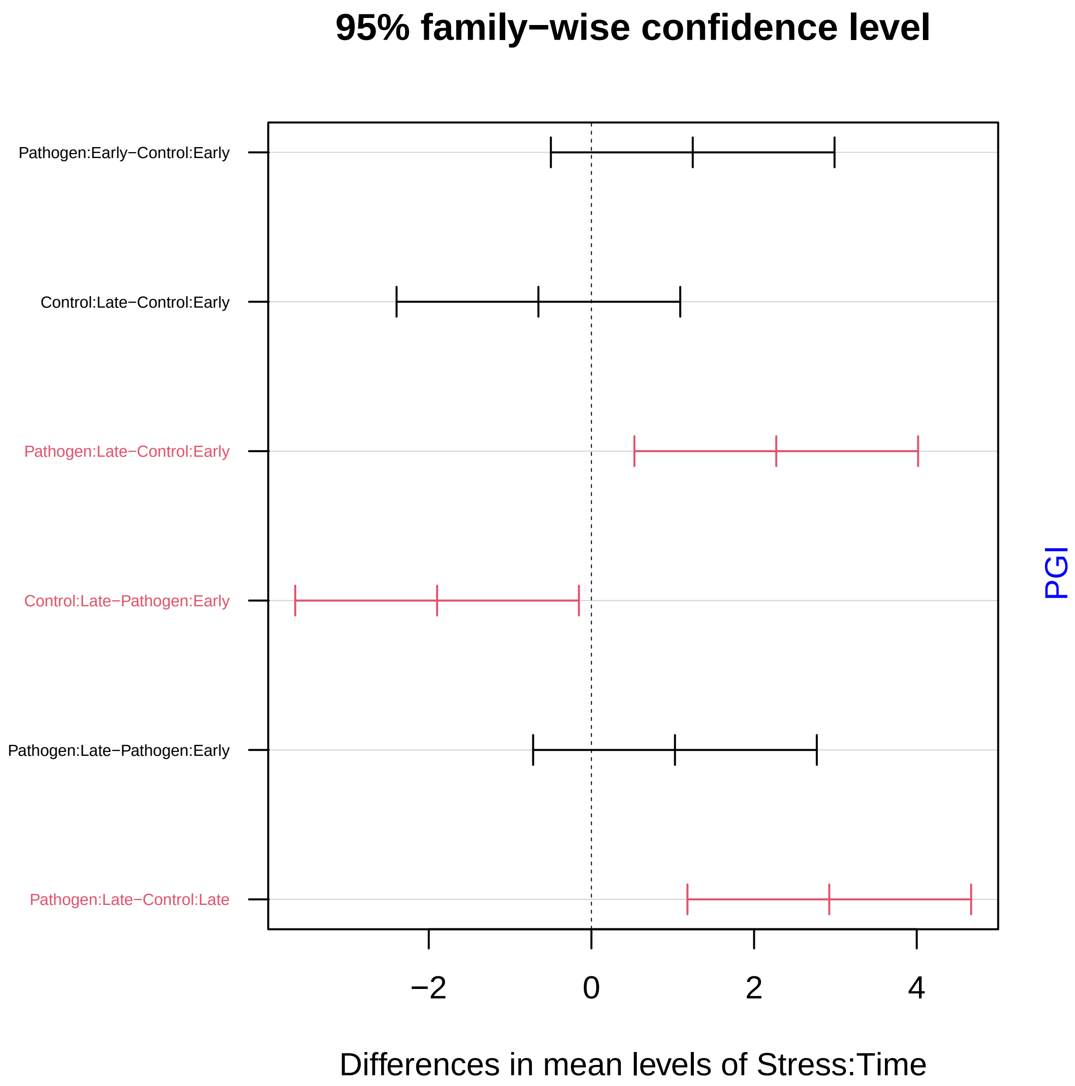
95% family−wise confidence level
Pathogen:Early−Control:Early
Control:Late−Control:Early
Pathogen:Late−Control:Early
Control:Late−Pathogen:Early
Pathogen:Late−Pathogen:Early
Pathogen:Late−Control:Late
−2
0
2
4
Differences in mean levels of Stress:Time
PGI

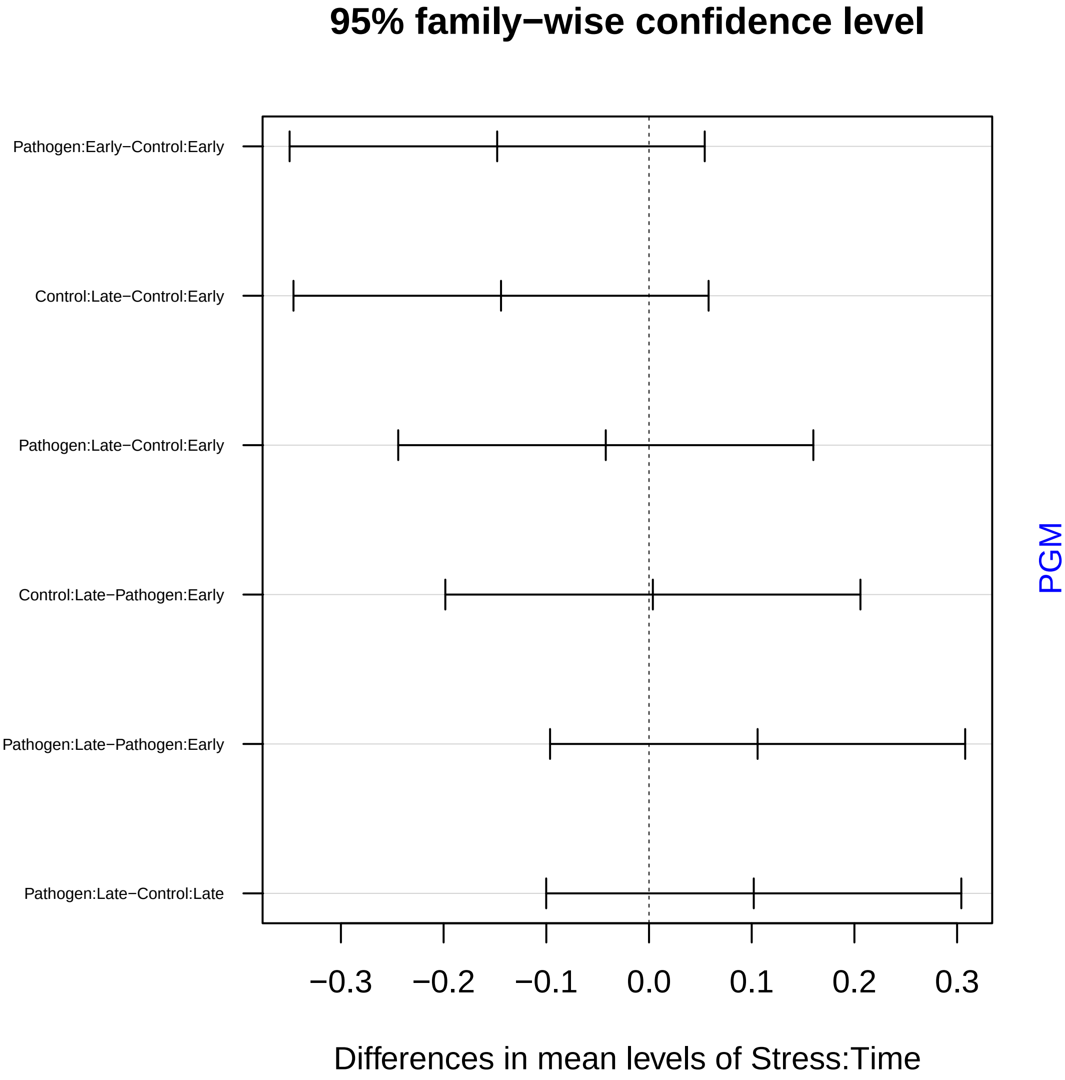

95% family−wise confidence level
Pathogen:Early−Control:Early
Control:Late−Control:Early
Pathogen:Late−Control:Early
Control:Late−Pathogen:Early
Pathogen:Late−Pathogen:Early
Pathogen:Late−Control:Late
−0.3
−0.2
−0.1
0.0
0.1
0.2
0.3
Differences in mean levels of Stress:Time
PGM

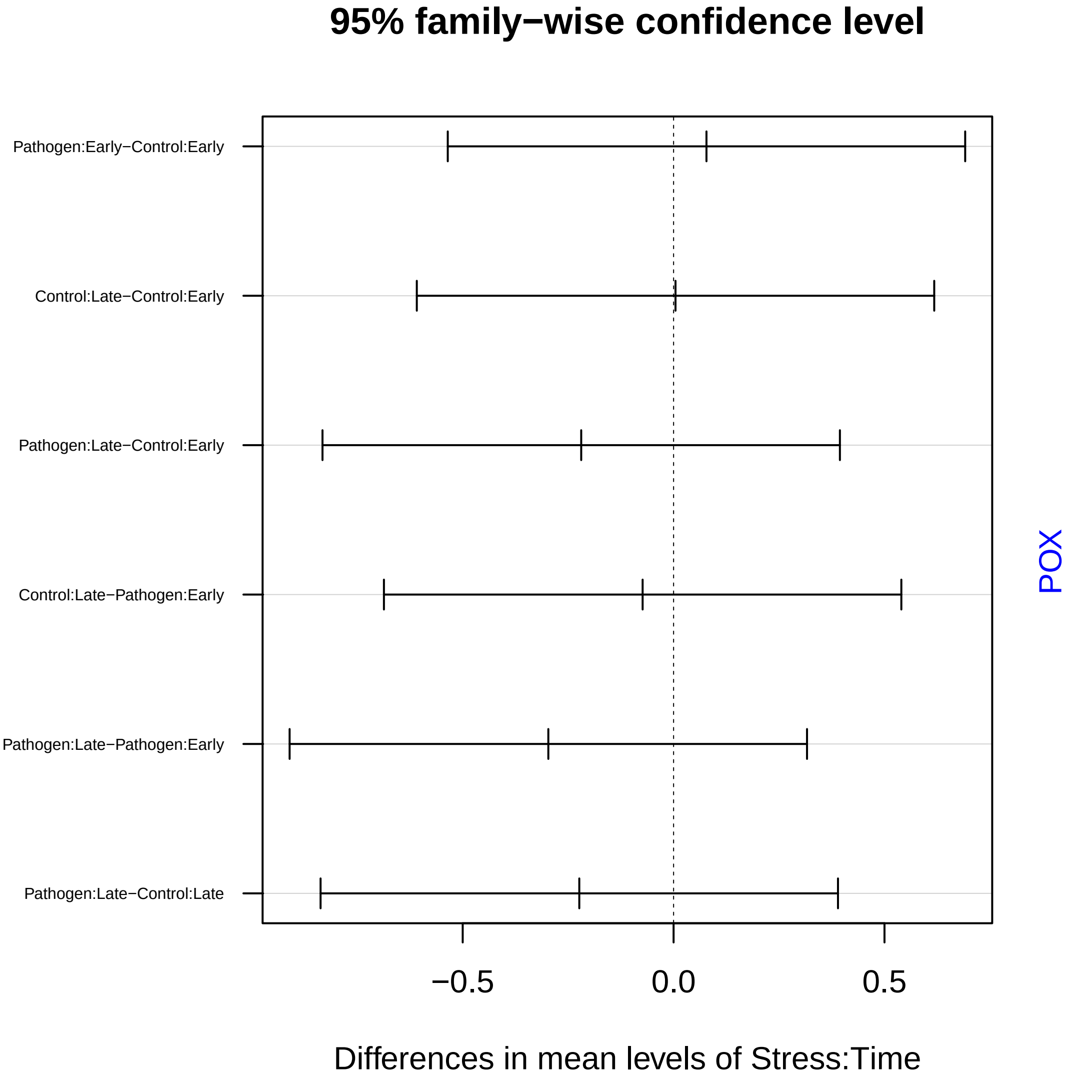

95% family−wise confidence level
Pathogen:Early−Control:Early
Control:Late−Control:Early
Pathogen:Late−Control:Early
Control:Late−Pathogen:Early
Pathogen:Late−Pathogen:Early
Pathogen:Late−Control:Late
−0.5
0.0
0.5
Differences in mean levels of Stress:Time
POX

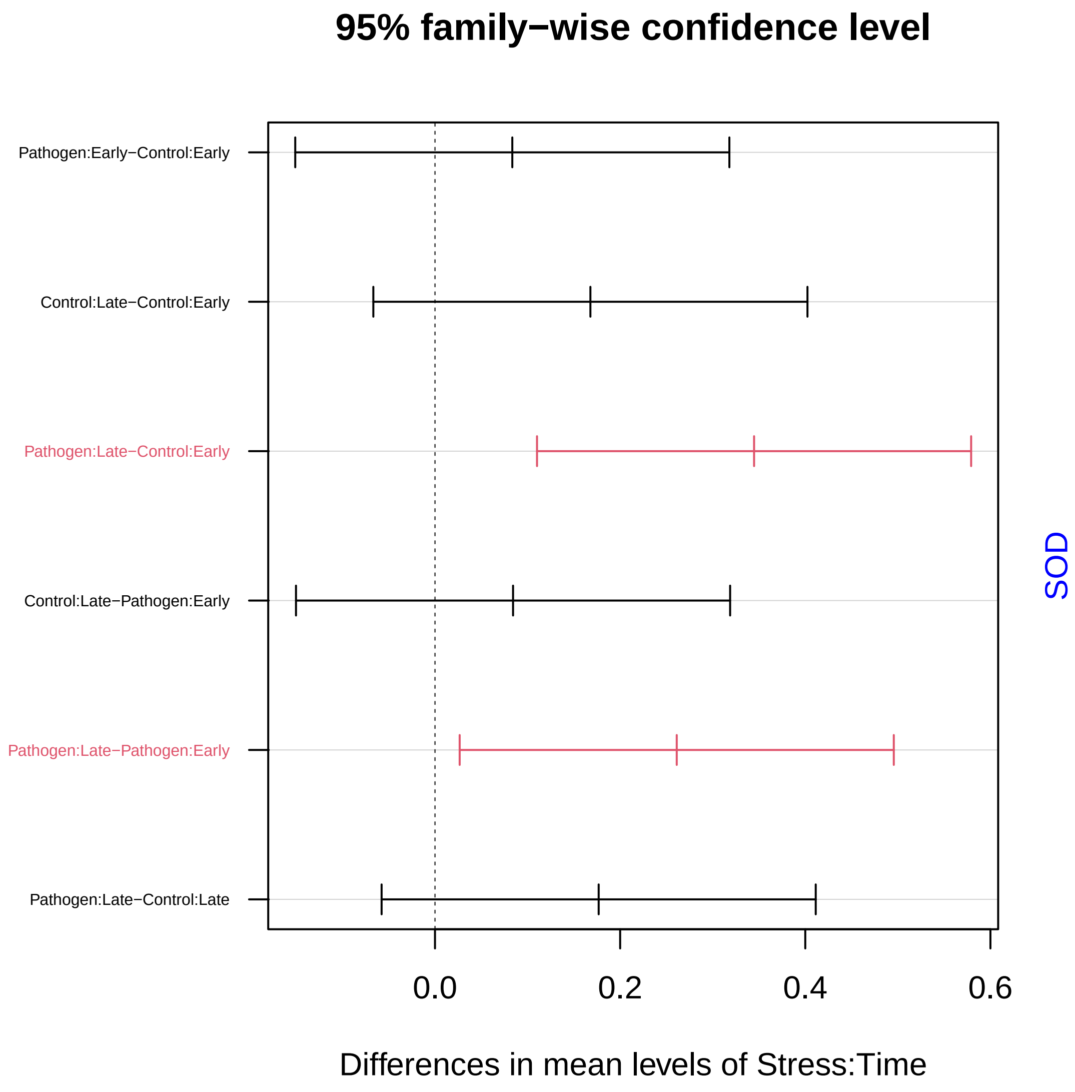
95% family−wise confidence level
Pathogen:Early−Control:Early
Control:Late−Control:Early
Pathogen:Late−Control:Early
Control:Late−Pathogen:Early
Pathogen:Late−Pathogen:Early
Pathogen:Late−Control:Late
0.0
0.2
0.4
0.6
Differences in mean levels of Stress:Time
SOD

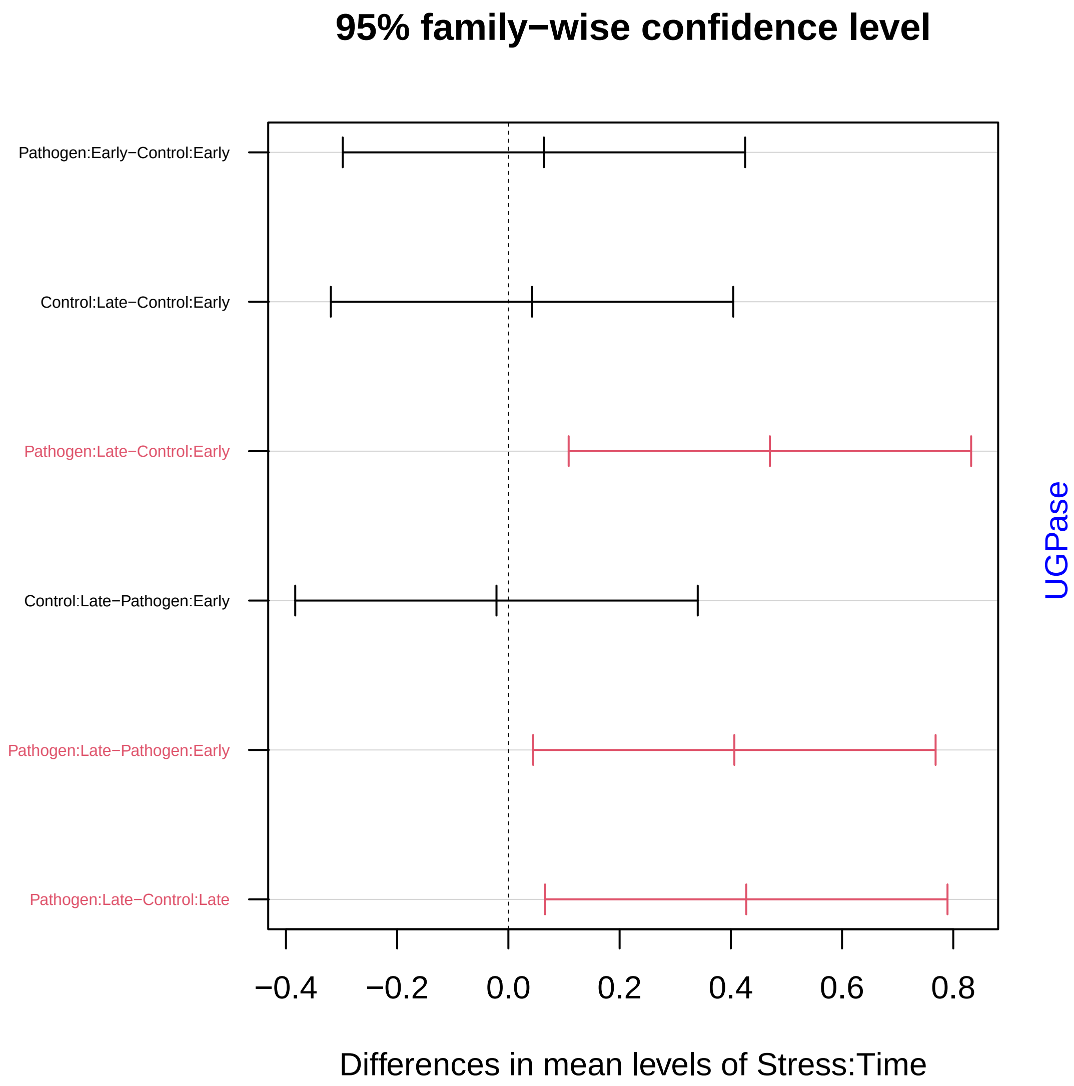
95% family−wise confidence level
Pathogen:Early−Control:Early
Control:Late−Control:Early
Pathogen:Late−Control:Early
Control:Late−Pathogen:Early
Pathogen:Late−Pathogen:Early
Pathogen:Late−Control:Late
−0.4
−0.2
0.0
0.2
0.4
0.6
0.8
Differences in mean levels of Stress:Time
UGPase
