# Supplementary material for: Candidate pathogenicity factor/effector proteins of ‘Candidatus Phytoplasma solani’ modulate plant carbohydrate metabolism, accelerate the ascorbate–glutathione cycle, and induce autophagosomes
Source: Front Plant Sci. 2023 Aug 18;14:1232367. doi: 10.3389/fpls.2023.1232367 (PMC10471893; doi:10.3389/fpls.2023.1232367)
Supplement: Supplementary file 3 [file DataSheet_3.pdf]

Supplemental Information

Title: Candidate pathogenicity factor/effector proteins of '*Candidatus* Phytoplasma solani' modulate plant carbohydrate metabolism, accelerate the ascorbate-glutathione cycle and induce autophagosomes

Authors: Marina Dermastia*, Špela Tomaž, Rebeka Strah, Tjaša Lukan, Anna Coll, Barbara Dušak, Barbara Anžič, Timotej Čepin, Stefanie Wienkoop, Aleš Kladnik, Maja Zagorščak, Monika Riedle-Bauer, Christina Schönhuber, Wolfram Weckwerth, Kristina Gruden, Thomas Roitsch, Maruša Pompe Novak, Günter Brader

* Correspondence: marina.dermastia@nib.si

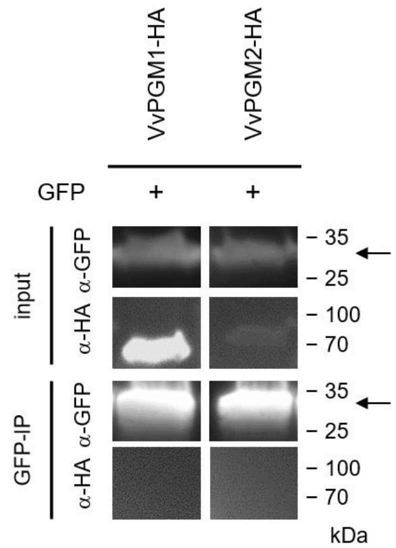

Supplemental Fig. S3. Co-immunoprecipitation assay negative controls. The HA-labeled grapevine phosphoglucomutases (VvPGMs) were co-expressed with GFP (+), to show they do not interact with fluorescent proteins after immunoprecipitation (GFP-IP). Detection of proteins in leaf protein extracts is shown as control (input). Arrows indicate expected bands.
