# Supplementary material for: Candidate pathogenicity factor/effector proteins of ‘Candidatus Phytoplasma solani’ modulate plant carbohydrate metabolism, accelerate the ascorbate–glutathione cycle, and induce autophagosomes
Source: Front Plant Sci. 2023 Aug 18;14:1232367. doi: 10.3389/fpls.2023.1232367 (PMC10471893; doi:10.3389/fpls.2023.1232367)
Supplement: Supplementary file 4 [file DataSheet_4.pdf]

Supplemental Information

Title: Candidate pathogenicity factor/effector proteins of '*Candidatus* Phytoplasma solani' modulate plant carbohydrate metabolism, accelerate the ascorbate-glutathione cycle and induce autophagosomes

Authors: Marina Dermastia*, Špela Tomaž, Rebeka Strah, Tjaša Lukan, Anna Coll, Barbara Dušak, Barbara Anžič, Timotej Čepin, Stefanie Wienkoop, Aleš Kladnik, Maja Zagorščak, Monika Riedle-Bauer, Christina Schönhuber, Wolfram Weckwerth, Kristina Gruden, Thomas Roitsch, Maruša Pompe Novak, Günter Brader

* Correspondence: marina.dermastia@nib.si

Catalase

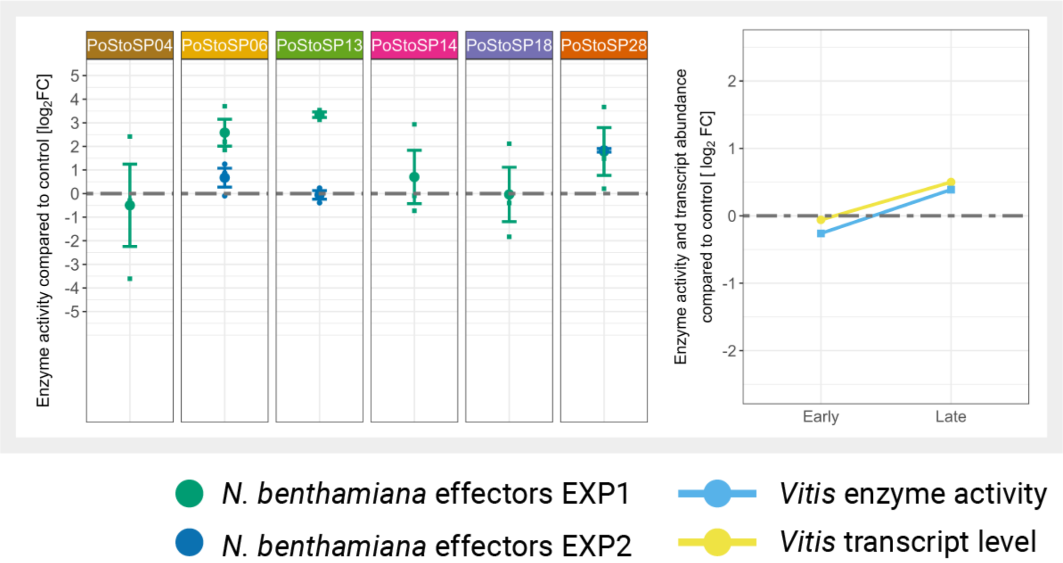

Supplemental Fig. S4. Catalase activity. Enzyme activities after transient transformation of *N. benthamiana* with different effector constructs in inoculated leaves 14 days after transformation compared to control are shown in the left subpanels. The grapevine enzyme activities together with the transcript abundance from samples infected with '*Ca*. P. solani' before (early) and after (late) symptom development compared to control are shown in the right subpanels. Transcription values correspond to *Vitvi*18g00095 (Dermastia *et al*., 2021).
