# Supplementary material for: Candidate pathogenicity factor/effector proteins of ‘Candidatus Phytoplasma solani’ modulate plant carbohydrate metabolism, accelerate the ascorbate–glutathione cycle, and induce autophagosomes
Source: Front Plant Sci. 2023 Aug 18;14:1232367. doi: 10.3389/fpls.2023.1232367 (PMC10471893; doi:10.3389/fpls.2023.1232367)
Supplement: Supplementary file 6 [file DataSheet_6.pdf]

Supplemental Information

Title: Candidate effector proteins of '*Candidatus* Phytoplasma solani' modulate plant carbohydrate metabolism, accelerate ascorbate-glutathione cycle and induce autophagosomes

Authors: Marina Dermastia*, Špela Tomaž, Rebeka Strah, Tjaša Lukan, Anna Coll, Barbara Dušak, Barbara Anžič, Timotej Čepin, Stefanie Wienkoop, Aleš Kladnik, Maja Zagorščak, Monika Riedle-Bauer, Christina Schönhuber, Wolfram Weckwerth, Kristina Gruden, Thomas Roitsch, Maruša Pompe Novak, Günter Brader

* Correspondence: marina.dermastia@nib.si

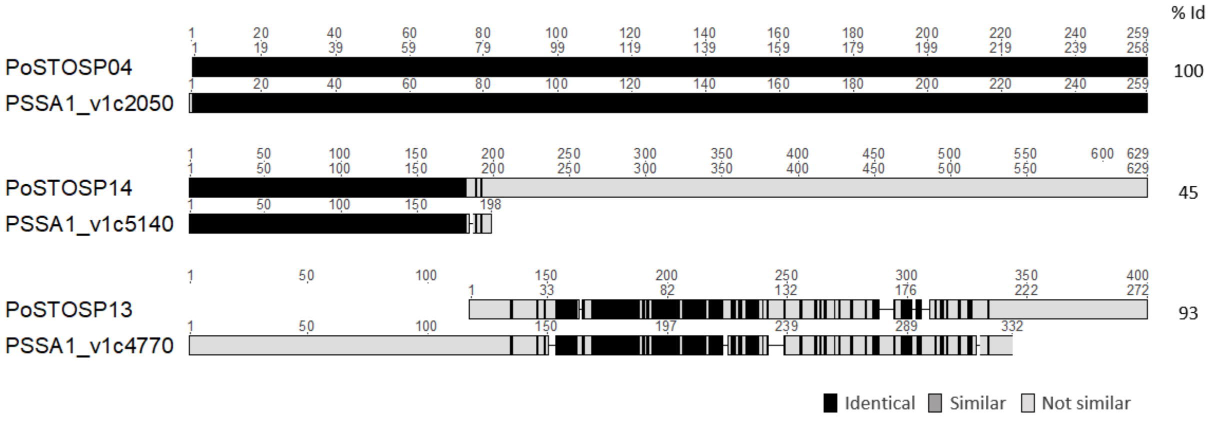

Supplemental Fig. S6. Pairwise sequence alignments of candidate effector proteins. PoSTOSP04, PoSTOSP13 and PoSTOSP14 align or partially align with previously identified '*Ca*. P. solani' strain SA-1 effector sequences (Music Seruga et al. 2019). The alignments are colored with the Geneious Prime (https://www.geneious.com) sequence similarity color scheme, based on the identity score matrix. Pairwise sequence identity (% ID) of the aligned regions is specified for each alignment. Sequence numbering (amino acid) is shown above each alignment.
