# Supplementary material for: Candidate pathogenicity factor/effector proteins of ‘Candidatus Phytoplasma solani’ modulate plant carbohydrate metabolism, accelerate the ascorbate–glutathione cycle, and induce autophagosomes
Source: Front Plant Sci. 2023 Aug 18;14:1232367. doi: 10.3389/fpls.2023.1232367 (PMC10471893; doi:10.3389/fpls.2023.1232367)
Supplement: Supplementary file 7 [file DataSheet_7.pdf]

Supplemental Information

Title: Candidate effector proteins of '*Candidatus* Phytoplasma solani' modulate plant carbohydrate metabolism, accelerate ascorbate-glutathione cycle and induce autophagosomes

Authors: Marina Dermastia*, Špela Tomaž, Rebeka Strah, Tjaša Lukan, Anna Coll, Barbara Dušak, Barbara Anžič, Timotej Čepin, Stefanie Wienkoop, Aleš Kladnik, Maja Zagorščak, Monika Riedle-Bauer, Christina Schönhuber, Wolfram Weckwerth, Kristina Gruden, Thomas Roitsch, Maruša Pompe Novak, Günter Brader

* Correspondence: marina.dermastia@nib.si

Supplemental Table S1. List of primers

Target	Primer name	Primer sequence 5'-3'	Purpose
PoStoSP04	PoStoSP04_ FP	CACCATGAGAAATAACAAAATAAAAATAAAATTTAAATTG	cloning into pENTR and colony PCR
	PoStoSP04_ RP	TTATTCTATTGTGTTTACAAAAGTACAAAGTTTTAC	
PoStoSP06	PoStoSP06_ FP	CACCATGATCTTGGATCTTAACTTTTCTAAAAGAAAAT	
	PoStoSP06_ RP	TTAGTCTTCATTTTCTTTAGGTTTATCTTGAACAT	
PoStoSP13	PoStoSP13_FP	CACCATGCATTTAAGAAAAAAACGCTTTTTT	
	PoStoSP13_ RP	TTAAAATAAAAAATTGTCTAAAAAAACGAAACAG	
PoStoSP14	PoStoSP14 _FP	CACCATGCATTTAAGAAAAAAACGCTTTTTT	
	PoStoSP14_ RP	TTATTTTTTAACTTTTTGAAAACAATAATATCCAG	
PoStoSP18	PoStoSP18_ FP	CACCATGGTATTAAAGGGTAAATTAAAAATAATATTTTTTTTTG	
	PoStoSP18_ RP	TTAAGAAAGAGCTTTTTTTTGAGCAGC	
PoStoSP28	PoStoSP28_ FP	CACCATGCAAAACACAAAAAAATCTTTAGTTATTAAATT	
	PoStoSP28_ RP	TTATTGTCTTCTTTTTTTAATAACAAAGAAAGCAAC	
Vitvi01g00455	Vv455_pENTR_Fw	CACCATGGTGATGTTCAAGGTTTCTCGAGTC	cloning into pENTR
	Vv455_pENTR_Rv_noSTOP	TGTGATAACAGTGGGAGCAGAACG	
Vitvi16g00891	Vv891_pENTR_Fw	CACCATGGATAGTGTCTTCTTTTCAACCCTCA	
	Vv891_pENTR_Rv_noSTOP	TGTGATCACTGTAGGTTTCTCCCTTC	
Vitvi01g00455	F_Vitvi01g00455_pho	ATGGTGATGTTCAAGGTTTCTCGAGTC	cloning into pJET
	R_Vitvi01g00455_pho	CTATGTGATAACAGTGGGAGCAGAAC	
Vitvi16g00891	F_Vitvi16g00891_pho	ATGGATAGTGTCTTCTTTTCAACCCTCA	
	R_Vitvi16g00891_pho	TTATGTGATCACTGTAGGTTTCTCCCTTC	
Vitvi01g00455	Vv455_middle_Fw	CTTTGAGGTCCCAACTGGCTGG	sequencing
	Vv455_middle_Rv	CGCTTTGCATAAGCTCCACCAA	
Vitvi16g00891	Vv891_middle_Fw	TTGAGGTCCCCACTGGATGGAAATTT	
	Vv891_middle_Rv	AGAAAGATCAATGTCTGGGATGTCTACC	
pENTR	M13 F	GTAAAACGACGGCCAGT	
	M13 R	CAGGAAACAGCTATGACC	
pJET1.2/blunt	Forward Sequencing Primer	CGACTCACTATAGGGAGAGCGGC	
	Reverse Sequencing Primer	AAGAACATCGATTTTCCATGGCAG	
