# Supplementary material for: Candidate pathogenicity factor/effector proteins of ‘Candidatus Phytoplasma solani’ modulate plant carbohydrate metabolism, accelerate the ascorbate–glutathione cycle, and induce autophagosomes
Source: Front Plant Sci. 2023 Aug 18;14:1232367. doi: 10.3389/fpls.2023.1232367 (PMC10471893; doi:10.3389/fpls.2023.1232367)
Supplement: Supplementary file 8 [file DataSheet_8.pdf]

Supplemental Information

Title: Candidate effector proteins of ‘Candidatus Phytoplasma solani’ modulate plant carbohydrate metabolism, accelerate ascorbate-glutathione cycle and induce autophagosomes

Authors: Marina Dermastia*, Špela Tomaž, Rebeka Strah, Tjaša Lukan, Anna Coll, Barbara Dušak, Barbara Anžič, Timotej Čepin, Stefanie Wienkoop, Aleš Kladnik, Maja Zagorščak, Monika Riedle-Bauer, Christina Schönhuber, Wolfram Weckwerth, Kristina Gruden, Thomas Roitsch, Maruša Pompe Novak, Günter Brader

Supplemental Table S3. Protein groups identified through protein pull-down and mass spectrometry analysis. A list of proteins with their UniProtKB identifiers and fasta headers, identified in the YFP-tagged candidate effector PoSTOSP28 sample, but not present in control sample after pull-down.

Protein IDs	Fasta headers	Number of proteins	Peptides
tr|Q8LSZ3|Q8LSZ3_TOBAC;tr|A0A1S4A5R2|A0A1S4A5R2_TOBAC;tr|A0A1S4B618|A0A1S4B618_TOBAC;tr|A0A1S3ZIP8|A0A1S3ZIP8_TOBAC	tr|Q8LSZ3|Q8LSZ3_TOBAC NADPH-protochlorophyllide oxidoreductase OS=Nicotiana tabacum OX=4097 GN=POR1 PE=2 SV=1;tr|A0A1S4A5R2|A0A1S4A5R2_TOBAC NADPH-protochlorophyllide oxidoreductase OS=Nicotiana tabacum OX=4097 GN=LOC107793976 PE=3 SV=1;tr|A0A1S4B618|A0A1	4	3
tr|A0A140G1T9|A0A140G1T9_TOBAC	tr|A0A140G1T9|A0A140G1T9_TOBAC ribosomal protein S18 OS=Nicotiana tabacum OX=4097 GN=rps18 PE=3 SV=1	1	2
tr|A0A1S3ZRR1|A0A1S3ZRR1_TOBAC;tr|A0A1S3YLV9|A0A1S3YLV9_TOBAC	tr|A0A1S3ZRR1|A0A1S3ZRR1_TOBAC nucleoid-associated protein At4g30620, chloroplastic-like OS=Nicotiana tabacum OX=4097 GN=LOC107789621 PE=4 SV=1;tr|A0A1S3YLV9|A0A1S3YLV9_TOBAC nucleoid-associated protein At4g30620, chloroplastic-like OS=Nicotiana tabacum OX	2	2
tr|A0A1S4B4M6|A0A1S4B4M6_TOBAC;tr|A0A1S4BHP4|A0A1S4BHP4_TOBAC;tr|A0A1S3Z6E1|A0A1S3Z6E1_TOBAC	tr|A0A1S4B4M6|A0A1S4B4M6_TOBAC 26S proteasome non-ATPase regulatory subunit 1 homolog OS=Nicotiana tabacum OX=4097 GN=LOC107804491 PE=3 SV=1;tr|A0A1S4BHP4|A0A1S4BHP4_TOBAC 26S proteasome non-ATPase regulatory subunit 1 homolog OS=Nicotiana tabacum OX=4097	3	5
tr|A0A1S4AZ72|A0A1S4AZ72_TOBAC;tr|A0A1S3YKL7|A0A1S3YKL7_TOBAC	tr|A0A1S4AZ72|A0A1S4AZ72_TOBAC thioredoxin-like 3-1, chloroplastic OS=Nicotiana tabacum OX=4097 GN=LOC107802806 PE=4 SV=1;tr|A0A1S3YKL7|A0A1S3YKL7_TOBAC thioredoxin-like 3-1, chloroplastic OS=Nicotiana tabacum OX=4097 GN=LOC107777304 PE=4 SV=1	2	2
tr|A0A1S4C4F5|A0A1S4C4F5_TOBAC	tr|A0A1S4C4F5|A0A1S4C4F5_TOBAC glutamate-1-semialdehyde 2,1-aminomutase, chloroplastic-like OS=Nicotiana tabacum OX=4097 GN=LOC107815022 PE=4 SV=1	1	3
tr|A0A1S4CKA0|A0A1S4CKA0_TOBAC	tr|A0A1S4CKA0|A0A1S4CKA0_TOBAC uncharacterized protein LOC107819940 isoform X1 OS=Nicotiana tabacum OX=4097 GN=LOC107819940 PE=4 SV=1	1	1
tr|Q9AT16|Q9AT16_TOBAC;tr|Q8H6P9|Q8H6P9_TOBAC;tr|Q84P55|Q84P55_TOBAC;tr|Q84P56|Q84P56_TOBAC	tr|Q9AT16|Q9AT16_TOBAC Ankyrin-repeat protein HBP1 OS=Nicotiana tabacum OX=4097 GN=LOC107793888 PE=2 SV=1;tr|Q8H6P9|Q8H6P9_TOBAC Ankyrin domain protein OS=Nicotiana tabacum OX=4097 GN=ANK1 PE=2 SV=1;tr|Q84P55|Q84P55_TOBAC TGB12K interacting protein 3 OS=Ni	4	4
tr|A0A1S4B341|A0A1S4B341_TOBAC;tr|A0A1S4AGR7|A0A1S4AGR7_TOBAC	tr|A0A1S4B341|A0A1S4B341_TOBAC cell division protein FtsY homolog, chloroplastic-like OS=Nicotiana tabacum OX=4097 GN=LOC107804044 PE=3 SV=1;tr|A0A1S4AGR7|A0A1S4AGR7_TOBAC cell division protein FtsY homolog, chloroplastic-like OS=Nicotiana tabacum OX=4097	2	3
tr|A0A1S4D985|A0A1S4D985_TOBAC;tr|A0A1S4ABS2|A0A1S4ABS2_TOBAC	tr|A0A1S4D985|A0A1S4D985_TOBAC serine--glyoxylate aminotransferase-like OS=Nicotiana tabacum OX=4097 GN=LOC107827351 PE=3 SV=1;tr|A0A1S4ABS2|A0A1S4ABS2_TOBAC serine--glyoxylate aminotransferase OS=Nicotiana tabacum OX=4097 GN=LOC107795801 PE=3 SV=1	2	3
tr|A0A1S4DJC0|A0A1S4DJC0_TOBAC;tr|A0A1S3Z5N4|A0A1S3Z5N4_TOBAC;sp|Q9ZS34|CHLP_TOBAC;tr|A0A1S4DJC8|A0A1S4DJC8_TOBAC;tr|A0A1S4AZJ0|A0A1S4AZJ0_TOBAC;tr|A0A1S3XXZ2|A0A1S3XXZ2_TOBAC	tr|A0A1S4DJC0|A0A1S4DJC0_TOBAC geranylgeranyl diphosphate reductase, chloroplastic-like isoform X1 OS=Nicotiana tabacum OX=4097 GN=LOC107830453 PE=4 SV=1;tr|A0A1S3Z5N4|A0A1S3Z5N4_TOBAC geranylgeranyl diphosphate reductase, chloroplastic OS=Nicotiana tabacu	6	4
tr|A0A140G1V2|A0A140G1V2_TOBAC	tr|A0A140G1V2|A0A140G1V2_TOBAC ribosomal protein S8 OS=Nicotiana tabacum OX=4097 GN=rps8 PE=3 SV=1	1	2
tr|A0A1S4A2J0|A0A1S4A2J0_TOBAC;tr|A0A1S3ZTF8|A0A1S3ZTF8_TOBAC	tr|A0A1S4A2J0|A0A1S4A2J0_TOBAC cysteine desulfurase 1, chloroplastic-like OS=Nicotiana tabacum OX=4097 GN=LOC107793129 PE=4 SV=1;tr|A0A1S3ZTF8|A0A1S3ZTF8_TOBAC cysteine desulfurase 1, chloroplastic-like OS=Nicotiana tabacum OX=4097 GN=LOC107790197 PE=4 SV=	2	2
tr|A0A1S4CZJ7|A0A1S4CZJ7_TOBAC;tr|A0A1S3YAN6|A0A1S3YAN6_TOBAC	tr|A0A1S4CZJ7|A0A1S4CZJ7_TOBAC COBW domain-containing protein 1-like OS=Nicotiana tabacum OX=4097 GN=LOC107824330 PE=4 SV=1;tr|A0A1S3YAN6|A0A1S3YAN6_TOBAC COBW domain-containing protein 1-like OS=Nicotiana tabacum OX=4097 GN=LOC107774293 PE=4 SV=1	2	2
tr|A0A1S3Y035|A0A1S3Y035_TOBAC;tr|A0A1S4A0C9|A0A1S4A0C9_TOBAC	tr|A0A1S3Y035|A0A1S3Y035_TOBAC ribulose bisphosphate carboxylase/oxygenase activase, chloroplastic-like OS=Nicotiana tabacum OX=4097 GN=LOC107770767 PE=4 SV=1;tr|A0A1S4A0C9|A0A1S4A0C9_TOBAC ribulose bisphosphate carboxylase/oxygenase activase, chloroplasti	2	3
tr|Q9FES7|Q9FES7_TOBAC;tr|A0A1S3XEV7|A0A1S3XEV7_TOBAC;sp|O22436|CHLI_TOBAC;tr|A0A1S3Z7S1|A0A1S3Z7S1_TOBAC	tr|Q9FES7|Q9FES7_TOBAC Mg-protoporphyrin IX chelatase OS=Nicotiana tabacum OX=4097 GN=Su-s PE=2 SV=1;tr|A0A1S3XEV7|A0A1S3XEV7_TOBAC Mg-protoporphyrin IX chelatase OS=Nicotiana tabacum OX=4097 GN=LOC107764312 PE=3 SV=1;sp|O22436|CHLI_TOBAC Magnesium-chelata	4	3
tr|A0A1S4DKC4|A0A1S4DKC4_TOBAC;tr|A0A1S3YLS7|A0A1S3YLS7_TOBAC;tr|A0A1S4CLW9|A0A1S4CLW9_TOBAC;tr|A0A1S4AFU6|A0A1S4AFU6_TOBAC	tr|A0A1S4DKC4|A0A1S4DKC4_TOBAC chaperone protein dnaJ A6, chloroplastic-like OS=Nicotiana tabacum OX=4097 GN=LOC107830729 PE=4 SV=1;tr|A0A1S3YLS7|A0A1S3YLS7_TOBAC chaperone protein dnaJ A6, chloroplastic-like OS=Nicotiana tabacum OX=4097 GN=LOC107777513 PE	4	2
tr|A0A1S4BJ77|A0A1S4BJ77_TOBAC	tr|A0A1S4BJ77|A0A1S4BJ77_TOBAC Tryptophan synthase OS=Nicotiana tabacum OX=4097 GN=LOC107808897 PE=3 SV=1	1	2
tr|A0A1S4CMV5|A0A1S4CMV5_TOBAC;tr|A0A1S4BWP8|A0A1S4BWP8_TOBAC;tr|A0A1S3ZRW6|A0A1S3ZRW6_TOBAC;tr|A0A1S4A6I2|A0A1S4A6I2_TOBAC;tr|A0A1S3ZTA5|A0A1S3ZTA5_TOBAC	tr|A0A1S4CMV5|A0A1S4CMV5_TOBAC 26S proteasome non-ATPase regulatory subunit 2 homolog OS=Nicotiana tabacum OX=4097 GN=LOC107820776 PE=3 SV=1;tr|A0A1S4BWP8|A0A1S4BWP8_TOBAC 26S proteasome non-ATPase regulatory subunit 2 homolog A-like OS=Nicotiana tabacum O	5	4
tr|A0A1S4AZ18|A0A1S4AZ18_TOBAC;tr|A0A1S3YZJ0|A0A1S3YZJ0_TOBAC	tr|A0A1S4AZ18|A0A1S4AZ18_TOBAC ATP sulfurylase 1, chloroplastic-like OS=Nicotiana tabacum OX=4097 GN=LOC107802851 PE=4 SV=1;tr|A0A1S3YZJ0|A0A1S3YZJ0_TOBAC ATP sulfurylase 1, chloroplastic-like OS=Nicotiana tabacum OX=4097 GN=LOC107781534 PE=4 SV=1	2	5
tr|A0A076L2E2|A0A076L2E2_TOBAC;tr|A0A1S3ZK73|A0A1S3ZK73_TOBAC	tr|A0A076L2E2|A0A076L2E2_TOBAC 40S ribosomal protein S25 OS=Nicotiana tabacum OX=4097 GN=LOC107768022 PE=2 SV=1;tr|A0A1S3ZK73|A0A1S3ZK73_TOBAC 40S ribosomal protein S25-like OS=Nicotiana tabacum OX=4097 GN=LOC107787568 PE=4 SV=1	2	2
tr|A0A1S4CGP3|A0A1S4CGP3_TOBAC;tr|A0A1S3X430|A0A1S3X430_TOBAC;tr|A0A1S4A807|A0A1S4A807_TOBAC;tr|A0A1S4AP69|A0A1S4AP69_TOBAC;tr|A0A1S4B9L9|A0A1S4B9L9_TOBAC	tr|A0A1S4CGP3|A0A1S4CGP3_TOBAC 60S ribosomal protein L14-2-like OS=Nicotiana tabacum OX=4097 GN=LOC107818765 PE=4 SV=1;tr|A0A1S3X430|A0A1S3X430_TOBAC probable 60S ribosomal protein L14 OS=Nicotiana tabacum OX=4097 GN=LOC107760861 PE=4 SV=1;tr|A0A1S4A807|A0	5	4
tr|A0A1S3ZEQ4|A0A1S3ZEQ4_TOBAC	tr|A0A1S3ZEQ4|A0A1S3ZEQ4_TOBAC GDSL esterase/lipase At1g29670-like OS=Nicotiana tabacum OX=4097 GN=LOC107785876 PE=4 SV=1	1	1
tr|A0A1S4AJL5|A0A1S4AJL5_TOBAC;tr|A0A1S4CRI6|A0A1S4CRI6_TOBAC;tr|A0A1S4AJC6|A0A1S4AJC6_TOBAC;tr|A0A1S4CRF2|A0A1S4CRF2_TOBAC;tr|A0A1S4BGN4|A0A1S4BGN4_TOBAC;tr|A0A1S3XYY2|A0A1S3XYY2_TOBAC	tr|A0A1S4AJL5|A0A1S4AJL5_TOBAC 26S proteasome non-ATPase regulatory subunit 4 homolog isoform X1 OS=Nicotiana tabacum OX=4097 GN=LOC107798338 PE=4 SV=1;tr|A0A1S4CRI6|A0A1S4CRI6_TOBAC 26S proteasome non-ATPase regulatory subunit 4 homolog isoform X2 OS=Nico	6	3
tr|A0A1S4CQ71|A0A1S4CQ71_TOBAC;tr|A0A1S4AA87|A0A1S4AA87_TOBAC;sp|Q05214|ACT1_TOBAC;tr|A0A1S4BAV7|A0A1S4BAV7_TOBAC;tr|A0A1S4C557|A0A1S4C557_TOBAC;tr|A0A1S4C1C0|A0A1S4C1C0_TOBAC;tr|A0A1S3ZT51|A0A1S3ZT51_TOBAC;sp|P93371|ACT5_TOBAC;tr|A0A1S3X7J8|A0A1S3X7J8_TOBAC;tr|A0A1S3YSA4|A0A1S3YSA4_TOBAC	tr|A0A1S4CQ71|A0A1S4CQ71_TOBAC actin-like OS=Nicotiana tabacum OX=4097 GN=LOC107821481 PE=3 SV=1;tr|A0A1S4AA87|A0A1S4AA87_TOBAC actin OS=Nicotiana tabacum OX=4097 GN=LOC107795436 PE=3 SV=1;sp|Q05214|ACT1_TOBAC Actin OS=Nicotiana tabacum OX=4097 PE=3 SV=1;t	10	11
tr|A0A1S3XE72|A0A1S3XE72_TOBAC;tr|A0A1S4DPW9|A0A1S4DPW9_TOBAC	tr|A0A1S3XE72|A0A1S3XE72_TOBAC 60S ribosomal protein L22-3 OS=Nicotiana tabacum OX=4097 GN=LOC107764146 PE=4 SV=1;tr|A0A1S4DPW9|A0A1S4DPW9_TOBAC 60S ribosomal protein L22-3-like OS=Nicotiana tabacum OX=4097 GN=LOC107832173 PE=4 SV=1	2	1
tr|A0A1S3YFA7|A0A1S3YFA7_TOBAC;tr|A0A1S3YF92|A0A1S3YF92_TOBAC;tr|A0A1S3ZYZ3|A0A1S3ZYZ3_TOBAC	tr|A0A1S3YFA7|A0A1S3YFA7_TOBAC flagellar radial spoke protein 5-like isoform X2 OS=Nicotiana tabacum OX=4097 GN=LOC107775472 PE=4 SV=1;tr|A0A1S3YF92|A0A1S3YF92_TOBAC flagellar radial spoke protein 5-like isoform X1 OS=Nicotiana tabacum OX=4097 GN=LOC107775	3	3
tr|Q6QND3|Q6QND3_TOBAC;tr|A0A1S4B745|A0A1S4B745_TOBAC;tr|A0A1S4CJY5|A0A1S4CJY5_TOBAC;tr|A0A1S3XXA7|A0A1S3XXA7_TOBAC	tr|Q6QND3|Q6QND3_TOBAC Putative pyridoxine biosynthesis protein isoform A OS=Nicotiana tabacum OX=4097 GN=Pdx1-B PE=3 SV=1;tr|A0A1S4B745|A0A1S4B745_TOBAC pyridoxal 5-phosphate synthase subunit PDX1 OS=Nicotiana tabacum OX=4097 GN=LOC107805188 PE=3 SV=1	4	3
tr|A0A1S4DD77|A0A1S4DD77_TOBAC;tr|A0A1S4CCG1|A0A1S4CCG1_TOBAC;sp|O80361|RK4_TOBAC	tr|A0A1S4DD77|A0A1S4DD77_TOBAC 50S ribosomal protein L4, chloroplastic-like OS=Nicotiana tabacum OX=4097 GN=LOC107828567 PE=3 SV=1;tr|A0A1S4CCG1|A0A1S4CCG1_TOBAC 50S ribosomal protein L4, chloroplastic OS=Nicotiana tabacum OX=4097 GN=LOC107817573 PE=3 SV=1	3	2
tr|A0A1S4D6G4|A0A1S4D6G4_TOBAC;tr|A0A1S3XGJ0|A0A1S3XGJ0_TOBAC	tr|A0A1S4D6G4|A0A1S4D6G4_TOBAC 40S ribosomal protein S30 OS=Nicotiana tabacum OX=4097 GN=LOC107826518 PE=3 SV=1;tr|A0A1S3XGJ0|A0A1S3XGJ0_TOBAC 40S ribosomal protein S30 OS=Nicotiana tabacum OX=4097 GN=LOC107764876 PE=3 SV=1	2	1
tr|A0A1S4CLB7|A0A1S4CLB7_TOBAC	tr|A0A1S4CLB7|A0A1S4CLB7_TOBAC aspartate-semialdehyde dehydrogenase-like OS=Nicotiana tabacum OX=4097 GN=LOC107820046 PE=3 SV=1	1	1
tr|A0A1S4A4D2|A0A1S4A4D2_TOBAC;tr|A0A1S4ATT2|A0A1S4ATT2_TOBAC;tr|A0A1S4A4N5|A0A1S4A4N5_TOBAC	tr|A0A1S4A4D2|A0A1S4A4D2_TOBAC fe-S cluster assembly factor HCF101, chloroplastic-like isoform X2 OS=Nicotiana tabacum OX=4097 GN=LOC107793652 PE=3 SV=1;tr|A0A1S4ATT2|A0A1S4ATT2_TOBAC fe-S cluster assembly factor HCF101, chloroplastic-like OS=Nicotiana tab	3	2
tr|A0A1S3XBY2|A0A1S3XBY2_TOBAC;tr|A0A1S3X8Q5|A0A1S3X8Q5_TOBAC;sp|O80362|RK10_TOBAC	tr|A0A1S3XBY2|A0A1S3XBY2_TOBAC 50S ribosomal protein L10, chloroplastic OS=Nicotiana tabacum OX=4097 GN=LOC107763454 PE=3 SV=1;tr|A0A1S3X8Q5|A0A1S3X8Q5_TOBAC 50S ribosomal protein L10, chloroplastic-like OS=Nicotiana tabacum OX=4097 GN=LOC107762422 PE=3 SV	3	2
tr|A0A1S3ZVR6|A0A1S3ZVR6_TOBAC;sp|P27494|CB23_TOBAC;tr|A0A1S3YPE9|A0A1S3YPE9_TOBAC;tr|A0A1S3ZSU2|A0A1S3ZSU2_TOBAC;tr|A0A1S3ZVQ8|A0A1S3ZVQ8_TOBAC;tr|A0A1S4BMB0|A0A1S4BMB0_TOBAC;tr|A0A1S3ZYV8|A0A1S3ZYV8_TOBAC;tr|A0A1S3XEU1|A0A1S3XEU1_TOBAC;tr|A0A1S3YWG7|A0A1S3YWG7_TOBAC;tr|A0A1S3Y974|A0A1S3Y974_TOBAC;sp|P27491|CB27_TOBAC;tr|A0A1S3Y8B9|A0A1S3Y8B9_TOBAC;tr|A0A1S3Y8Q2|A0A1S3Y8Q2_TOBAC;tr|A0A1S4BC47|A0A1S4BC47_TOBAC;sp|P27495|CB24_TOBAC;sp|P27496|CB25_TOBAC;tr|A0A1S3Y100|A0A1S3Y100_TOBAC;sp|P27492|CB21_TOBAC;tr|A0A1S3YD73|A0A1S3YD73_TOBAC;tr|A0A1S4BG28|A0A1S4BG28_TOBAC;tr|A0A1S4BG47|A0A1S4BG47_TOBAC;tr|A0A1S4BPJ2|A0A1S4BPJ2_TOBAC;tr|A0A1S4A8I3|A0A1S4A8I3_TOBAC;tr|A0A1S4BG25|A0A1S4BG25_TOBAC;tr|A0A1S4BPJ4|A0A1S4BPJ4_TOBAC;sp|P27493|CB22_TOBAC;tr|A0A1S4BPM1|A0A1S4BPM1_TOBAC;tr|A0A1S3Y6H9|A0A1S3Y6H9_TOBAC;tr|A0A1S3Y8R2|A0A1S3Y8R2_TOBAC;tr|A0A1S3Y8G4|A0A1S3Y8G4_TOBAC;tr|A0A1S3Y820|A0A1S3Y820_TOBAC	tr|A0A1S3ZVR6|A0A1S3ZVR6_TOBAC Chlorophyll a-b binding protein, chloroplastic OS=Nicotiana tabacum OX=4097 GN=LOC107790955 PE=3 SV=1;sp|P27494|CB23_TOBAC Chlorophyll a-b binding protein 36, chloroplastic OS=Nicotiana tabacum OX=4097 GN=CAB36 PE=3 SV=1;tr|A	31	2
tr|A0A1S4D708|A0A1S4D708_TOBAC;tr|A0A1S4D6S4|A0A1S4D6S4_TOBAC;tr|A0A1S4D2I0|A0A1S4D2I0_TOBAC;tr|A0A1S4D2Z3|A0A1S4D2Z3_TOBAC	tr|A0A1S4D708|A0A1S4D708_TOBAC uncharacterized protein ycf39-like isoform X2 OS=Nicotiana tabacum OX=4097 GN=LOC107826653 PE=4 SV=1;tr|A0A1S4D6S4|A0A1S4D6S4_TOBAC uncharacterized protein ycf39-like isoform X1 OS=Nicotiana tabacum OX=4097 GN=LOC107826653 PE	4	2
tr|A0A1S4CJ62|A0A1S4CJ62_TOBAC;tr|A0A1S4C068|A0A1S4C068_TOBAC;tr|A0A1S4AYN5|A0A1S4AYN5_TOBAC;tr|A0A1S3XV78|A0A1S3XV78_TOBAC	tr|A0A1S4CJ62|A0A1S4CJ62_TOBAC 60S ribosomal protein L9-1-like OS=Nicotiana tabacum OX=4097 GN=LOC107819630 PE=4 SV=1;tr|A0A1S4C068|A0A1S4C068_TOBAC 60S ribosomal protein L9-1-like OS=Nicotiana tabacum OX=4097 GN=LOC107813756 PE=4 SV=1;tr|A0A1S4AYN5|A0A1S4	4	2
tr|A0A1S4D9E4|A0A1S4D9E4_TOBAC;tr|A0A1S4CYL2|A0A1S4CYL2_TOBAC	tr|A0A1S4D9E4|A0A1S4D9E4_TOBAC pheophytinase, chloroplastic-like OS=Nicotiana tabacum OX=4097 GN=LOC107827246 PE=4 SV=1;tr|A0A1S4CYL2|A0A1S4CYL2_TOBAC pheophytinase, chloroplastic-like OS=Nicotiana tabacum OX=4097 GN=LOC107824051 PE=4 SV=1	2	2
tr|A0A1S3ZX13|A0A1S3ZX13_TOBAC;tr|A0A1S3WYR8|A0A1S3WYR8_TOBAC	tr|A0A1S3ZX13|A0A1S3ZX13_TOBAC Fructose-bisphosphate aldolase OS=Nicotiana tabacum OX=4097 GN=LOC107791431 PE=3 SV=1;tr|A0A1S3WYR8|A0A1S3WYR8_TOBAC Fructose-bisphosphate aldolase OS=Nicotiana tabacum OX=4097 GN=LOC107759369 PE=3 SV=1	2	7
tr|A0A1S3XHG4|A0A1S3XHG4_TOBAC;tr|A0A1S4AIH6|A0A1S4AIH6_TOBAC;tr|A0A1S4A6S4|A0A1S4A6S4_TOBAC	tr|A0A1S3XHG4|A0A1S3XHG4_TOBAC phosphoglucomutase, cytoplasmic-like OS=Nicotiana tabacum OX=4097 GN=LOC107765291 PE=4 SV=1;tr|A0A1S4AIH6|A0A1S4AIH6_TOBAC phosphoglucomutase, cytoplasmic-like OS=Nicotiana tabacum OX=4097 GN=LOC107798006 PE=4 SV=1;tr|A0A1S4A	3	1
tr|A0A1S3X0Y5|A0A1S3X0Y5_TOBAC	tr|A0A1S3X0Y5|A0A1S3X0Y5_TOBAC Ferredoxin--NADP reductase, chloroplastic OS=Nicotiana tabacum OX=4097 GN=LOC107760150 PE=3 SV=1	1	1
tr|A0A1S3Z6E6|A0A1S3Z6E6_TOBAC;tr|A0A1S4DC16|A0A1S4DC16_TOBAC	tr|A0A1S3Z6E6|A0A1S3Z6E6_TOBAC V-type proton ATPase subunit H OS=Nicotiana tabacum OX=4097 GN=LOC107783503 PE=3 SV=1;tr|A0A1S4DC16|A0A1S4DC16_TOBAC V-type proton ATPase subunit H OS=Nicotiana tabacum OX=4097 GN=LOC107828022 PE=3 SV=1	2	2
tr|A0A1S4CW25|A0A1S4CW25_TOBAC	tr|A0A1S4CW25|A0A1S4CW25_TOBAC probable inactive shikimate kinase like 2, chloroplastic OS=Nicotiana tabacum OX=4097 GN=LOC107823221 PE=4 SV=1	1	2
tr|A0A1S4DNT1|A0A1S4DNT1_TOBAC;tr|A0A1S3ZGF1|A0A1S3ZGF1_TOBAC;tr|A0A1S4DNS6|A0A1S4DNS6_TOBAC	tr|A0A1S4DNT1|A0A1S4DNT1_TOBAC Eukaryotic translation initiation factor 3 subunit I OS=Nicotiana tabacum OX=4097 GN=LOC107831796 PE=3 SV=1;tr|A0A1S3ZGF1|A0A1S3ZGF1_TOBAC Eukaryotic translation initiation factor 3 subunit I OS=Nicotiana tabacum OX=4097 GN=L	3	2
tr|A0A140G1Q1|A0A140G1Q1_TOBAC	tr|A0A140G1Q1|A0A140G1Q1_TOBAC ribosomal protein S2 OS=Nicotiana tabacum OX=4097 GN=rps2 PE=3 SV=1	1	2
tr|A0A1S4DC28|A0A1S4DC28_TOBAC;tr|A0A1S3X053|A0A1S3X053_TOBAC;tr|A0A1S3WZM7|A0A1S3WZM7_TOBAC;tr|A0A1S4DC59|A0A1S4DC59_TOBAC	tr|A0A1S4DC28|A0A1S4DC28_TOBAC glutathionyl-hydroquinone reductase YqjG-like isoform X2 OS=Nicotiana tabacum OX=4097 GN=LOC107828221 PE=4 SV=1;tr|A0A1S3X053|A0A1S3X053_TOBAC glutathionyl-hydroquinone reductase YqjG-like isoform X2 OS=Nicotiana tabacum OX=4	4	2
tr|A0A1S3XCY9|A0A1S3XCY9_TOBAC;tr|A0A1S4C7S3|A0A1S4C7S3_TOBAC	tr|A0A1S3XCY9|A0A1S3XCY9_TOBAC Protein transport protein Sec61 subunit beta OS=Nicotiana tabacum OX=4097 GN=LOC107763814 PE=3 SV=1;tr|A0A1S4C7S3|A0A1S4C7S3_TOBAC Protein transport protein Sec61 subunit beta OS=Nicotiana tabacum OX=4097 GN=LOC107816002 PE=3	2	1
tr|A0A1S4C8W0|A0A1S4C8W0_TOBAC;tr|A0A1S4C9D8|A0A1S4C9D8_TOBAC;tr|A0A1S4CX44|A0A1S4CX44_TOBAC;tr|A0A1S4C8W4|A0A1S4C8W4_TOBAC;tr|A0A1S3Z7W7|A0A1S3Z7W7_TOBAC;tr|A0A1S4AZJ1|A0A1S4AZJ1_TOBAC;tr|A0A1S3Z2N3|A0A1S3Z2N3_TOBAC;tr|A0A1S3Z750|A0A1S3Z750_TOBAC	tr|A0A1S4C8W0|A0A1S4C8W0_TOBAC dihydrolipoyl dehydrogenase 2, chloroplastic-like isoform X3 OS=Nicotiana tabacum OX=4097 GN=LOC107816379 PE=3 SV=1;tr|A0A1S4C9D8|A0A1S4C9D8_TOBAC dihydrolipoyl dehydrogenase 2, chloroplastic-like isoform X2 OS=Nicotiana taba	8	1
tr|A0A1S4ARQ1|A0A1S4ARQ1_TOBAC;tr|A0A1S3Z100|A0A1S3Z100_TOBAC	tr|A0A1S4ARQ1|A0A1S4ARQ1_TOBAC calcium sensing receptor, chloroplastic-like OS=Nicotiana tabacum OX=4097 GN=LOC107800572 PE=4 SV=1;tr|A0A1S3Z100|A0A1S3Z100_TOBAC calcium sensing receptor, chloroplastic-like OS=Nicotiana tabacum OX=4097 GN=LOC107781793 PE=4	2	2
tr|A0A1S4A473|A0A1S4A473_TOBAC;tr|A0A1S3XMD5|A0A1S3XMD5_TOBAC;tr|A0A1S4AA32|A0A1S4AA32_TOBAC;tr|A0A1S3YRY3|A0A1S3YRY3_TOBAC;tr|A0A1S3YRG8|A0A1S3YRG8_TOBAC	tr|A0A1S4A473|A0A1S4A473_TOBAC ATP-citrate synthase beta chain protein 2-like OS=Nicotiana tabacum OX=4097 GN=LOC107793546 PE=4 SV=1;tr|A0A1S3XMD5|A0A1S3XMD5_TOBAC ATP-citrate synthase beta chain protein 2-like OS=Nicotiana tabacum OX=4097 GN=LOC107766682	5	3
tr|A0A1S3ZKZ4|A0A1S3ZKZ4_TOBAC;tr|A0A1S3Z828|A0A1S3Z828_TOBAC;sp|Q42962|PGKY_TOBAC;tr|A0A1S4CGR1|A0A1S4CGR1_TOBAC;tr|A0A1S4BTZ7|A0A1S4BTZ7_TOBAC	tr|A0A1S3ZKZ4|A0A1S3ZKZ4_TOBAC Phosphoglycerate kinase OS=Nicotiana tabacum OX=4097 GN=LOC107787830 PE=3 SV=1;tr|A0A1S3Z828|A0A1S3Z828_TOBAC Phosphoglycerate kinase OS=Nicotiana tabacum OX=4097 GN=LOC107783850 PE=3 SV=1;sp|Q42962|PGKY_TOBAC Phosphoglycerat	5	3
tr|A0A1S3Z6N1|A0A1S3Z6N1_TOBAC;tr|A0A1S3YPJ3|A0A1S3YPJ3_TOBAC	tr|A0A1S3Z6N1|A0A1S3Z6N1_TOBAC glyoxylate/succinic semialdehyde reductase 2, chloroplastic-like OS=Nicotiana tabacum OX=4097 GN=LOC107783419 PE=4 SV=1;tr|A0A1S3YPJ3|A0A1S3YPJ3_TOBAC glyoxylate/succinic semialdehyde reductase 2, chloroplastic-like OS=Nicoti	2	2
tr|A0A1S4AFK0|A0A1S4AFK0_TOBAC;tr|A0A1S4AKS8|A0A1S4AKS8_TOBAC	tr|A0A1S4AFK0|A0A1S4AFK0_TOBAC Thiamine thiazole synthase, chloroplastic OS=Nicotiana tabacum OX=4097 GN=LOC107797059 PE=3 SV=1;tr|A0A1S4AKS8|A0A1S4AKS8_TOBAC Thiamine thiazole synthase, chloroplastic OS=Nicotiana tabacum OX=4097 GN=LOC107798781 PE=3 SV=1	2	2
tr|A0A1S4DI23|A0A1S4DI23_TOBAC;tr|A0A1S4BRE4|A0A1S4BRE4_TOBAC;tr|A0A1S4BRG6|A0A1S4BRG6_TOBAC;tr|A0A1S4BRE8|A0A1S4BRE8_TOBAC;tr|A0A1S4BRE2|A0A1S4BRE2_TOBAC	tr|A0A1S4DI23|A0A1S4DI23_TOBAC glycine--tRNA ligase, chloroplastic/mitochondrial 2-like OS=Nicotiana tabacum OX=4097 GN=LOC107830076 PE=3 SV=1;tr|A0A1S4BRE4|A0A1S4BRE4_TOBAC glycine--tRNA ligase, chloroplastic/mitochondrial 2 isoform X4 OS=Nicotiana tabacu	5	2
tr|A0A1S4CNI5|A0A1S4CNI5_TOBAC;tr|A0A1S4C789|A0A1S4C789_TOBAC	tr|A0A1S4CNI5|A0A1S4CNI5_TOBAC presequence protease 1, chloroplastic/mitochondrial-like OS=Nicotiana tabacum OX=4097 GN=LOC107820951 PE=4 SV=1;tr|A0A1S4C789|A0A1S4C789_TOBAC presequence protease 1, chloroplastic/mitochondrial-like OS=Nicotiana tabacum OX=4	2	1
tr|A0A1S4DGZ9|A0A1S4DGZ9_TOBAC;tr|A0A1S3X395|A0A1S3X395_TOBAC	tr|A0A1S4DGZ9|A0A1S4DGZ9_TOBAC probable plastid-lipid-associated protein 13, chloroplastic OS=Nicotiana tabacum OX=4097 GN=LOC107829677 PE=4 SV=1;tr|A0A1S3X395|A0A1S3X395_TOBAC probable plastid-lipid-associated protein 13, chloroplastic OS=Nicotiana tabacu	2	2
tr|A0A1S3ZKM6|A0A1S3ZKM6_TOBAC;tr|A0A1S3YCR6|A0A1S3YCR6_TOBAC;tr|Q84QE8|Q84QE8_TOBAC;tr|A0A1S4CLK5|A0A1S4CLK5_TOBAC;tr|A0A1S3ZHB8|A0A1S3ZHB8_TOBAC;tr|A0A1S3XLS3|A0A1S3XLS3_TOBAC;sp|Q40459|PSBO_TOBAC;tr|A0A1S4BSA2|A0A1S4BSA2_TOBAC	tr|A0A1S3ZKM6|A0A1S3ZKM6_TOBAC oxygen-evolving enhancer protein 1, chloroplastic-like OS=Nicotiana tabacum OX=4097 GN=LOC107787712 PE=4 SV=1;tr|A0A1S3YCR6|A0A1S3YCR6_TOBAC oxygen-evolving enhancer protein 1, chloroplastic-like OS=Nicotiana tabacum OX=4097	8	1
tr|A0A1S3XIX6|A0A1S3XIX6_TOBAC;tr|A0A1S4BMB4|A0A1S4BMB4_TOBAC	tr|A0A1S3XIX6|A0A1S3XIX6_TOBAC 2,3-dimethylmalate lyase-like OS=Nicotiana tabacum OX=4097 GN=LOC107765651 PE=4 SV=1;tr|A0A1S4BMB4|A0A1S4BMB4_TOBAC 2,3-dimethylmalate lyase-like OS=Nicotiana tabacum OX=4097 GN=LOC107809819 PE=4 SV=1	2	1
tr|A0A1S4CIK7|A0A1S4CIK7_TOBAC;tr|A0A1S3YQ39|A0A1S3YQ39_TOBAC;tr|A0A1S3ZJY4|A0A1S3ZJY4_TOBAC;tr|A0A1S3Z039|A0A1S3Z039_TOBAC;tr|A0A1S4ANL0|A0A1S4ANL0_TOBAC;tr|A0A1S3YHK3|A0A1S3YHK3_TOBAC	tr|A0A1S4CIK7|A0A1S4CIK7_TOBAC ATP-citrate synthase alpha chain protein 2-like OS=Nicotiana tabacum OX=4097 GN=LOC107819406 PE=4 SV=1;tr|A0A1S3YQ39|A0A1S3YQ39_TOBAC ATP-citrate synthase alpha chain protein 2 OS=Nicotiana tabacum OX=4097 GN=LOC107778559 PE=	6	2
